# Is There a Histone Code for Cellular Quiescence?

**DOI:** 10.3389/fcell.2021.739780

**Published:** 2021-10-29

**Authors:** Kenya Bonitto, Kirthana Sarathy, Kaiser Atai, Mithun Mitra, Hilary A. Coller

**Affiliations:** ^1^Department of Molecular, Cell, and Developmental Biology, University of California, Los Angeles, Los Angeles, CA, United States; ^2^Molecular Biology Interdepartmental Doctoral Program, University of California, Los Angeles, Los Angeles, CA, United States; ^3^Department of Biological Chemistry, David Geffen School of Medicine, University of California, Los Angeles, Los Angeles, CA, United States; ^4^Molecular Biology Institute, University of California, Los Angeles, Los Angeles, CA, United States

**Keywords:** histone post translational modification, quiescence, histone methylation, histone acetylation, metabolism, histone code

## Abstract

Many of the cells in our bodies are quiescent, that is, temporarily not dividing. Under certain physiological conditions such as during tissue repair and maintenance, quiescent cells receive the appropriate stimulus and are induced to enter the cell cycle. The ability of cells to successfully transition into and out of a quiescent state is crucial for many biological processes including wound healing, stem cell maintenance, and immunological responses. Across species and tissues, transcriptional, epigenetic, and chromosomal changes associated with the transition between proliferation and quiescence have been analyzed, and some consistent changes associated with quiescence have been identified. Histone modifications have been shown to play a role in chromatin packing and accessibility, nucleosome mobility, gene expression, and chromosome arrangement. In this review, we critically evaluate the role of different histone marks in these processes during quiescence entry and exit. We consider different model systems for quiescence, each of the most frequently monitored candidate histone marks, and the role of their writers, erasers and readers. We highlight data that support these marks contributing to the changes observed with quiescence. We specifically ask whether there is a quiescence histone “code,” a mechanism whereby the language encoded by specific combinations of histone marks is read and relayed downstream to modulate cell state and function. We conclude by highlighting emerging technologies that can be applied to gain greater insight into the role of a histone code for quiescence.

## Cellular Quiescence: A State of Reversible Cell Cycle Exit

To maintain physiological homeostasis, many tissues contain a population of cells that can exit the proliferative cell cycle and enter a quiescent state of temporary cell division arrest in response to anti-proliferative cues (Li and Clevers, 2010; Cheung and Rando, 2013; Nakamura-Ishizu et al., 2014; Dhawan and Laxman, 2015; Sun and Buttitta, 2017; Sagot and Laporte, 2019a; Marescal and Cheeseman, 2020). This non-dividing state of cellular quiescence is defined by its reversibility, that is, quiescent cells can reenter the cell cycle upon receiving proliferative signals. Quiescent cells can be distinguished from other types of non-dividing cells such as senescent or terminally differentiated cells by their temporary exit from the cell cycle and high likelihood of proliferating in response to a triggering stimulus (Sang and Coller, 2009; Cheung and Rando, 2013; Terzi et al., 2016). Quiescent cells must therefore preserve the ability to proliferate at a later time, and protect themselves from entering irreversible states (Coller et al., 2006; Sang and Coller, 2009; Sang et al., 2010; Bjornson et al., 2012).

Cellular quiescence has been studied experimentally in multiple systems including yeast, cultured primary cells, and stem cells (Mitra et al., 2018a; Spain et al., 2018; Yang and Chi, 2018) (Table 1). Some of the gene expression, signaling, and functional changes observed with quiescence are likely specific for a cell type, while others are shared. Transcriptional changes with quiescence have been analyzed using cDNA libraries (Schneider et al., 1988; Coppock et al., 1993), microarrays (Venezia et al., 2004; Coller et al., 2006; Suh et al., 2012; Johnson et al., 2017), next generation sequencing (van Velthoven et al., 2017; Mitra et al., 2018b; Srivastava et al., 2018), and single-cell RNA sequencing methods (Kalakonda et al., 2008; Coller, 2019a). These studies demonstrated widespread gene expression changes with quiescence, some of which are functionally important for the quiescent state (Suh et al., 2012; Johnson et al., 2017; Lee H.N. et al., 2018; Mitra et al., 2018b). These gene expression changes include downregulation of genes involved in cell cycle progression and upregulation of stress response genes (Lemons et al., 2010; Legesse-Miller et al., 2012; Valcourt et al., 2012; Coller, 2019b). Other gene expression changes allow the cell to re-organize metabolic pathways in quiescent cells to better match the availability of nutrients and the metabolic needs of the cell (Lemons et al., 2010; Valcourt et al., 2012; Coller, 2019b). Disruption of these cellular mechanisms can contribute to the occurrence and progression of pathologies related to aging, developmental defects, and cancer (Tumpel and Rudolph, 2019).

**TABLE 1:** List of *in vitro* and *in vivo* quiescence models.

Quiescence model	Type	Model conditions	References
Yeast	Cell culture	Stationary phase isolation	Allen et al., 2006
Fission yeast	Cell culture	Nitrogen-induced starvation; Glucose deprivation	Hayashi et al., 2018; Zahedi et al., 2020
Human dermal fibroblasts	Cell culture	Serum-starvation; Contact-inhibition	Evertts et al., 2013a; Mitra et al., 2018a
Human lung fibroblasts	Cell culture	Mitogen withdrawal; Contact inhibition; Loss of adhesion	Coller et al., 2006; Dai et al., 2015
Bovine fibroblasts	Cell culture	Serum starvation	Meng et al., 2020; Kallingappa et al., 2016
Human/mouse embryonic stem cells	Cell culture	Isolation from inner cell mass of blastocyst	Khoa et al., 2020
Mouse hematopoietic stem cells	Tissue	Isolation from fetal liver, bone marrow, cord blood	Vizán et al., 2020; Tie et al., 2020
Mouse neural stem cells	Tissue	Isolation from ventricular-subventricular zone of brain	Kalamakis et al., 2019; Obernier et al., 2018
Mouse muscle skeletal cells	Tissue	Isolation from muscle of 2 month-old mice	Boonsanay et al., 2016; Ryall et al., 2015
Human primary myoblasts	Cell culture	Methylcellulose culture medium	Cheedipudi et al., 2015
Mouse hair follicle stem cells	Tissue	Isolation from back, belly, or scalpskin	Kang et al., 2020; Lee et al., 2016; Lien et al., 2011
Human Breast cancer MCF-7 cells	Cell culture	Hormone starvation; Serum starvation	Liu et al., 2017; Bierhoff et al., 2014
Mouse T cells	Tissue	Isolation from spleen	Rawlings et al., 2011
Mouse Fibroblasts	Cell culture	Serum deprivation	Grigoryev et al., 2004

In addition to gene expression changes, quiescence is also associated with changes in the packaging of DNA into chromatin. Eukaryotic chromatin can take on two forms—a more condensed and transcriptionally silent form called heterochromatin and a less condensed and more transcriptionally active form called euchromatin (DesJarlais and Tummino, 2016). Within these states, the extent of compaction can vary, for instance, mitotic chromosomes are extremely condensed. Studies using imaging, flow cytometry, Hi-C, and other methods have shown that entry to a quiescent state involves changes in nuclear size, chromatin compaction and 3D genome architecture (Bridger et al., 2000; Evertts et al., 2013a; Guidi et al., 2015; Criscione et al., 2016; Swygert et al., 2019, 2021). In yeast, the transition from exponential phase growth to stationary phase, a quiescent state achieved when yeast deplete their nutrients, is associated with downregulation of gene expression, a more condensed chromatin state (Martinez et al., 2004; Schafer et al., 2008), and more long-range chromosomal interactions (Swygert et al., 2019). In mammals, activation of quiescent lymphocytes is associated with an unpacking of condensed chromatin in a process that can be visualized with electron microscopy (Tokuyasu et al., 1968; Dardick et al., 1983; Setterfield et al., 1983; Grigoryev et al., 2004). Using circular dichroism, Chiu and Baserga reported a likely change to a more open chromatin structure as quiescent fibroblasts re-enter the cell cycle (Chiu and Baserga, 1975). In contrast, in one study, bovine fibroblasts were reported to have a more relaxed chromatin state in G_0_ (quiescent) compared with G_1_ cells (Kallingappa et al., 2016).

In addition to changes in gene expression and chromatin compaction, quiescence is also associated with a change in the positioning of chromosomes within the nucleus. In yeast, hyperclustering of telomeres has been reported with quiescence (Guidi et al., 2015; Laporte et al., 2016). When serum was removed from the culture medium of human fibroblasts, chromosomes were repositioned within 15 min in a process that required ATP, actin polymerization, and myosin (Mehta et al., 2010). In another study in human dermal fibroblasts, gene-poor chromosome 18 was found near the edge of the nucleus and gene-rich chromosome 19 was found in the center of the nucleus in proliferating cells. In serum-starved, quiescent fibroblasts, chromosome 18 shifted to a more central location in the nucleus, and there was no longer a difference in the positioning of chromosomes 18 and 19 (Bridger et al., 2000). Taken together, these findings demonstrate changes in gene expression, chromatin compaction and chromosome positioning within the nucleus in quiescent cells.

## Histone Post-Translational Modifications as a Possible Biological Code

### Nucleosome Structure and Histone Marks

Eukaryotic genomic DNA is tightly packed inside the nucleus. For mammalian chromosomes, this tight packing results in a 10,000-fold reduction in length (Kornberg and Lorch, 2020). The DNA in chromatin forms complexes with histone proteins that assemble the DNA strands into nucleosomes in a structure that resembles “beads on a string” with the nucleosomes (beads) representing the basic repeating unit of chromatin (Cutter and Hayes, 2015; Zhou et al., 2019; Ghoneim et al., 2021). Each core nucleosome consists of ∼147 base pairs (bp) of DNA in a left-handed super-helical conformation wrapped around an octamer of histone proteins (Zhou et al., 2019). The octamer consists of two copies each of histone proteins H2A, H2B, H3, and H4 with each of the two dimers of H2A-H2B interacting with either end of a (H3-H4)_2_ tetramer (H4-H3:H3-H4). The core nucleosome is flanked by 10-70 bp of linker DNA and usually a linker histone (H1) (Cutter and Hayes, 2015). The disordered N-terminal tails of all four histone proteins as well as the C-terminal tail of H2A protrude out from the nucleosome core and are sites of diverse post translational modifications (PTMs) or marks such as lysine and arginine methylation, lysine acetylation, and serine and threonine phosphorylation (Bannister and Kouzarides, 2011; Greer and Shi, 2012; Cutter and Hayes, 2015). These histone tails modulate charge, hydrophobicity, and steric access to chromatin (Ghoneim et al., 2021). Histone PTMs are added and removed by enzymatic proteins referred to as “writers” and “erasers,” respectively (Soshnev et al., 2016; Hyun et al., 2017; Husmann and Gozani, 2019). Histone PTMs serve as recognition sites for proteins (“readers”) that site-specifically bind to chromatin. In some cases, a single protein contains multiple domains and can act as both a reader for one type of PTM and a writer for another PTM (Smeenk and Mailand, 2016). The amino acid residues in the histone globular core can also be post-translationally modified and these core PTMs likely modulate interactions between histones and between histones and DNA (Tessarz and Kouzarides, 2014).

### What Are the Properties or Functions of Histone Marks?

Histone H3K4me3, H3K36me3, and H3K79me3 and ubiquitylation of H2B marks are often associated with active transcription (Black et al., 2012; Hyun et al., 2017), whereas H3K9me3, H3K27me3, H2A ubiquitylation on lysine 119, and H4K20 methylation are indicators of a silenced chromatin state with reduced gene expression (Black et al., 2012; Hyun et al., 2017). These properties of histone marks are related to the way they interact with chromatin and chromatin binding proteins. Histone PTMs can be envisioned to function by at least two broad categories of mechanisms (Bannister and Kouzarides, 2011). The first involves direct structural effects on the biomechanical properties of DNA (Bannister and Kouzarides, 2011). In this role, histone PTMs can affect the accessibility of DNA, and thus the binding of transcription factors or other proteins that bind enhancers and affect transcription (Bannister and Kouzarides, 2011). Such effects can occur, for instance, when histone PTMs disrupt electrostatic interactions between histones and DNA. Nucleosome core particle has an overall charge of −150 electrons that is contributed by DNA (−294 electrons) and histones (+ 144 electrons) (Norton et al., 1989; Cortini, 2016; Prakash and Fournier, 2018). Some histone PTMs that are associated with a more open chromatin state and increased gene expression reduce the positive charges on histones thereby leading to less effective screening of the negative charges on DNA (Prakash and Fournier, 2018). Acetylation, in particular, impairs the affinity of histones for DNA by neutralizing the positive charges and disrupting the ionic interactions between histones and DNA. This results in increased histone mobility and a more open chromatin conformation (Allfrey et al., 1964; Cosgrove et al., 2004). An open chromatin conformation facilitates access to transcription factors and other chromatin binding proteins (Bannister and Kouzarides, 2011). While some modifications such as acetylation may be expected to alter the ionic charge and thus chromatin compaction, others, including methylation, may have more modest impacts on charge and chromatin structure (Bannister and Kouzarides, 2011).

The second way in which histone PTMs can exert a functional effect is by regulating the binding of different chromatin factors. As one example, proteins with PHD fingers and Tudor family of domains can bind lysine methylations (Maurer-Stroh et al., 2003; Champagne and Kutateladze, 2009; Bannister and Kouzarides, 2011). In some cases, multiple different domains can recognize the same lysine methylation (Bannister and Kouzarides, 2011). Another example of histone PTM recognition is the binding of dimeric Heterochromatin Protein 1 (HP1) to the H3K9me3 mark via the chromodomain. This is associated with repressive architecture and chromatin compaction (Bannister et al., 2001; Lachner et al., 2001; Bannister and Kouzarides, 2011).

Genome-wide studies of histone marks have revealed that combinations of histone marks can be used to classify chromatin into different states (Black et al., 2012). In *Arabidopsis*, four chromatin states were identified (Roudier et al., 2011); in *Drosophila*, 5-9 states have been reported (Kharchenko et al., 2011; Riddle et al., 2011); while in human cells, up to 51 chromatin states have been defined (Ernst and Kellis, 2010). In human cells, chromatin states include promoter-associated, transcription-associated, active intergenic, large-scale repressed, and repeat-associated states, each of which have distinct histone marks and biological roles (Ernst and Kellis, 2010). Different promoter states were defined by patterns of H3K4 methylation, H3K79 methylation, H4K20 methylation, and acetylation (Black et al., 2012). One particular chromatin state that involves a specific combination of histone marks is the bivalent mark (Bernhart et al., 2016). Bivalent marks are often found in the promoters of developmentally regulated genes (Bernstein et al., 2006; Lesch et al., 2013), and are defined by the simultaneous presence of activating marks such as H3K4me1 or H3K4me3, and the repressive chromatin mark H3K27me3 (Voigt et al., 2013). Genes with bivalent marks are repressed, but pre-loaded with RNA polymerase that is “poised” for rapid expression in response to a relevant trigger (Mikkelsen et al., 2007; Margaritis and Holstege, 2008; Gaertner et al., 2012). More recent studies have identified combinatorial marks that establish zones within the nucleus that can be identified by combinations of proteins and histone marks (Takei et al., 2021). These nuclear zones include nuclear speckles, active chromatin, heterochromatin zones and zones within the nucleolus (Takei et al., 2021). The active chromatin zone, for example, was characterized by histone H3K9ac, H3K27ac, H4K16ac, RNA polymerase II serine 5 phosphorylation, and SF3A66 (Takei et al., 2021).

Consistent with the concept of nuclear zones, histone marks may allow for the patterning of chromatin into regions of approximately 0.5-1 megabases with similar properties, termed topological domains as identified by the Hi-C technique (Prakash and Fournier, 2018). Histone modifications have been found to cluster at the genome scale as DNA tends to fold into domains in which the all of the DNA in that domain is labeled with similar histone marks (Dixon et al., 2012; Rao et al., 2014; Barbieri et al., 2017). For instance, H3K4me3-rich, H3K27me3-rich and H3K9me3 rich regions have been found to segregate from each other, and to mark active genes, repressed genes and inactive chromatin, respectively (Prakash and Fournier, 2018). Thus, histone PTMs may be associated with multiple aspects of chromatin including the extent of local compaction, the extent of gene expression and the formation of chromatin domains.

### Do Histone Marks Create a Histone Code?

Biological codes that have been previously described include an input system that is translated into an output via adaptors (Prakash and Fournier, 2018). As one example, the genetic code translates sequences of nucleotide codons (input) into a sequence of amino acids (output) using the protein translation apparatus (adapter) (Prakash and Fournier, 2018). Histone PTMs have also been suggested to establish a biological code (Strahl and Allis, 2000; Allis and Jenuwein, 2016). According to the histone code hypothesis, the presence of specific histone marks, and in some cases, possible combinations of histone marks (inputs), provides information to reader proteins (adapters) that interpret the marks or combinations of marks to produce outputs such as gene activation or silencing, chromatin compaction, repair of DNA damage, cell division or differentiation (Strahl and Allis, 2000; Jenuwein and Allis, 2001; Turner, 2002; Smeenk and Mailand, 2016). Given that histone marks tend to be rapidly re-established after cell division (Evertts et al., 2013a, b), information about a cell’s state can be transmitted to descendant cells. Misreading of histone marks has been associated with cancer and developmental defects (Wang and Allis, 2009; Chi et al., 2010; Hyun et al., 2017).

One potential advantage of a histone code would be that combinations of histone marks could provide increased robustness to a system in which different inputs result in specific outcomes (Prakash and Fournier, 2018). Robustness can be achieved with cooperation and redundancy (Prakash and Fournier, 2018). A histone code has been hypothesized to provide a level of proofreading needed so that genes are not turned on or off inappropriately (Prakash and Fournier, 2018). If there are multiple independent histone marks that work in concert to achieve an outcome, then loss of one mark would have only a modest effect on the associated phenotypes (Prakash and Fournier, 2018). Further, comparing the use of histone marks in different species shows that histone modifications have evolutionarily conserved functions and play a similar functional role across eukaryotes (Ho et al., 2014; Prakash and Fournier, 2018).

**FIGURE 1:**
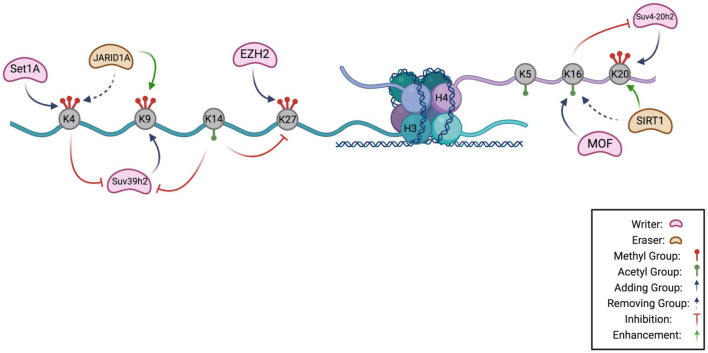
Histone Code Hypothesis. A schematic highlighting the known interactions between chromatin modifying enzymes on the H3 and H4 tail as a potential hypothesis of the histone code in quiescence. Lysines that can be methylated or acetylated are gray, and are indicated to have methyl groups (red) or acetyl groups (green). H3K4 can be methylated by methyltransferase, Set1A, and demethylated by JARID1A, a demethylase. Methylation of lysine 4 prevents the methylation of H3K9 by inhibiting Suv39h2, the methyltransferase of lysine 9. Suv39h2 activity can also be prevented by acetylation of H3K14. Removal of H3K4 methylation by JARID1A enhances H3K9 methylation. On the H4 tail, MOF acetylates lysine 16 which inhibits activity of Suv4-20h2 in methylating H4K20. Removal of an acetyl group from H4K16 by SIRT1 enhances H4K20 methylation (Figure made in BioRender).

Generating chromatin states with combinations of histone marks may reflect instances in which the presence of one histone PTM affects the recruitment of enzymes that create another PTM in the same or different histone tails resulting in reproducible output patterns (Figure 1). This can be achieved by multi-domain proteins that recognize the histone PTM through the reader domain and utilize a different domain for recruiting a histone writer. As one example, double-stranded DNA breaks serve as a signal to the ATM kinase, which leads to phosphorylation of the H2A variant H2A.X on its C terminal tail (Rogakou et al., 1998; Smeenk and Mailand, 2016). The presence of this mark, called γ-H2AX, creates binding sites for a reader for this mark, Mediator of DNA Damage Checkpoint Protein 1 (MDC1), a protein that recruits factors to the DNA damage site (Stucki et al., 2005), including E3 ligases that ubiquitinate histones (Huen et al., 2007; Kolas et al., 2007; Mailand et al., 2007; Doil et al., 2009). Histone ubiquitin modifications create recruitment platforms for DNA repair factors (Huen et al., 2007; Kolas et al., 2007; Mailand et al., 2007; Doil et al., 2009). Thus, histone modifications can transmit information about the site-specific presence of double strand breaks to affect an outcome, in this case, DNA repair.

Another example in which the presence of a histone mark affects the likelihood of other marks being added occurs during the mitosis phase of the cell cycle. Phosphorylation of Ser10 of histone H3 regulates transcription during interphase (Shimada et al., 2008) and chromosome condensation during mitosis (Wei et al., 1999). Histone H3S10 phosphorylation prevents phosphorylation of Thr6 and Thr11 on the same histone (Cosgrove, 2012; Liokatis et al., 2012). This hierarchy may ensure that phosphorylation of Ser10 during mitosis, which is required for chromosome condensation and separation, does not lead to subsequent formation of dually labeled histones with H3S10 phosphorylation and Thr6 or Thr11 phosphorylation during mitosis (Cosgrove, 2012; Liokatis et al., 2012). This example provides an instance in which histone marks antagonize each other, and combinations of histone marks are required to ensure a robust functional response.

Histone codes that lead to biologically significant outcomes have been suggested to play a role in neural plasticity (Farrelly and Maze, 2019), genome structure (Prakash and Fournier, 2018), and cancer (Godley and Le Beau, 2012). In neurons, serotonylation of histone H3 glutamine 5 works in conjunction with nearby H3K4me3 marks to regulate transcription (Farrelly et al., 2019; Farrelly and Maze, 2019). The dual H3K4(me3)(Q5serotonin) mark was found to enhance binding of interacting proteins, including the transcription factor complex TFIID (Lauberth et al., 2013; Farrelly et al., 2019; Farrelly and Maze, 2019). In this way, the combination of histone marks results in increased transcription of specific nearby genes.

On the other hand, it is important to note that the model that specific combinations of histone tail PTMs lead to defined biological outcomes has been challenged (Henikoff and Shilatifard, 2011; Morgan and Shilatifard, 2020). One central argument is whether histone modifications are the cause of different transcriptional states or, instead, are formed as a consequence of transcription and other dynamic processes. It has been difficult to resolve this controversy because studying the direct role of a histone PTM is challenging. Traditional genetic tools such as knockdown or overexpression are not sufficient to differentiate the direct versus indirect effects of targeting a histone modifying enzyme that adds or removes a PTM because the enzyme may also act on non-histone substrates (Henikoff and Shilatifard, 2011; Cornett et al., 2019; Corvalan and Coller, 2021). An alternative strategy to directly assess the role of a histone PTM is to mutate the amino acid residue that bears the PTM. While this strategy can be effective in some organisms such as yeast, it is not practical for higher eukaryotes due to the presence of multiple copies of the genes encoding the most frequently modified histones (Tripputi et al., 1986; Henikoff and Shilatifard, 2011; Soshnev et al., 2016; Corvalan and Coller, 2021).

Debate about the existence of a histone code has also centered on the nature of the histone code. The original paper describing a histone code suggested a code that has been described as hardwired and deterministic (Jenuwein and Allis, 2001), like the genetic code (Morgan and Shilatifard, 2020). With time, an alternative and more complex relationship between histone marks and functional outcomes has been described (Morgan and Shilatifard, 2020). In this potential representation, histone PTMs convey information in a context-dependent manner (Morgan and Shilatifard, 2020). A histone mark can have multiple potential outcomes, and the specific path would depend on multiple factors including the three-dimensional folding of the genome, the local chromatin environment, and the concentrations of the possible downstream effector molecules (Morgan and Shilatifard, 2020).

### Is There a Histone Post-translational Modification Code for Quiescence?

Does a histone code exist for quiescence? Are there specific patterns of histone tail PTMs that dictate or are associated with entry, exit, maintenance, or depth of a quiescent state? If so, do these histone PTMs modulate the physical properties of the DNA? Do these histone marks directly alter the chromatin accessibility of gene promoters and enhancers to induce molecular and phenotypic changes with quiescence? Alternatively, do these histone PTMs serve as binding sites for readers that recognize the PTMs and effect cellular changes during quiescence? If the histone marks serve as recognition sites, what are the most important effectors and what aspects of quiescence do they control? In this review, we address these questions and compare the findings from multiple experimental models of quiescence (Table 1). Advances in ChIP-seq technology (O’Geen et al., 2011) such as CUT&RUN (Hainer and Fazzio, 2019), CUT&Tag (Kaya-Okur et al., 2019), and HiChIP (Yan et al., 2014) have enabled fine resolution mapping of the genomic position of different histone tail marks (Table 2). Mass spectrometry can be used to measure histone epigenetic mark abundance and dynamics in a multiplexed, parallel manner (Volker-Albert et al., 2018) (Table 2). Further, advances in imaging such as combining immunofluorescence with directly labeled histone modification-specific antibodies to monitor histone levels in single cells (Hayashi-Takanaka et al., 2020), sequential fluorescence *in situ* hybridization analysis (Takei et al., 2021), and improved imaging with Stochastic Optical Reconstruction Microscopy (STORM) (Xu et al., 2018), have permitted detailed analysis of the global and site-specific organization of histone marks (Takei et al., 2021). It is important to note that in this review we focus on the most intensively studied histone PTMs, but these are not the only possible candidates by which histones or a histone code could contribute to the changes observed with quiescence. For instance, there may be new histone marks that are specific for quiescence and have not yet been observed, despite mass spectrometry-based histone tail analysis (Evertts et al., 2013a). There are also linker histones and variants of core histones that affect nucleosome structure and function, and consequently chromatin architecture (Kurumizaka et al., 2021). Changes in the compositions of histones and histone linkers could potentially contribute to the functional attributes of quiescent cells, and these are not reviewed here. We conclude by identifying areas for future studies and methodologies that can be used to address existing gaps in our knowledge.

**TABLE 2:** Methods to study histone marks.

Approach	Method(s)	Description	Amount of material	Bulk or single-cell	Global pattern?	References
PCR	ChIP-qPCR	Chromatin is cross-linked, fragmented, and immunoprecipitated (ChIP). DNA is isolated and purified and undergoes PCR	5 × 10^5^ – 5 × 10^6^ cells	Bulk	No	Milne et al., 2009
High-throughput sequencing	ChIP-seq	Following ChIP, Next-generation sequencing (NGS) is used to identify DNA fragments and map them against entire genome	10^5^ – 5 × 10^5^ cells per antibody	Bulk	Yes	O’Geen et al., 2011
	CUT&RUN	Recombinant protein A-micrococcal nuclease fusion recruited to the antibody targeting chromatin protein of interest; DNA fragments near antibody sites are cleaved, released, and sequenced	5 × 10^5^ cells	Bulk	Yes	Hainer and Fazzio, 2019
	CUT&Tag	A-Tn5 transposase fusion protein bound to antibody; transposase generates fragment libraries for sequencing	100,000 – 500,000 cells	Bulk	Yes	Kaya-Okur et al., 2019
	Joint RNA-seq and CUT&Tag (Paired-Tag)	CUT&Tag followed by RNA-seq: profiling of histone modifications and transcripts in single cells; generates maps of chromatin state and transcript in various tissues by cell type	∼10,000 cells per antibody	Single-cell	Yes	Zhu et al., 2021
	HiCHIP	Comprehensive analysis of single-end and paired-end ChIP-seq reads for protein-DNA interactions	10^6^ – 15 × 10^6^ cells	Bulk	Yes	Yan et al., 2014
	RNA-seq	RNA is isolated from sample and converted into cDNA libraries which undergo NGS.	5 × 10^4^ – 5 × 10^6^ cells	Bulk and single-cell	Yes	Wang et al., 2009; Svensson et al., 2018
Imaging	Multicolor IF-based single cell analysis	Using directly labeled histone modification-specific antibodies to monitor histone levels in single cells			No	Hayashi-Takanaka et al., 2020
	Stochastic Optical Reconstruction Microscopy (STORM)	Single fluorophores blink individually and randomly, enabling precise location of photons, eventually forming full images			No	Xu et al., 2018
Mass spectrometry	LC-MS/MS	Allows for quantification of histone modifications and combinations of modifications	10^6^ – 10^7^ cells	single-cell	Yes	Volker-Albert et al., 2018
Flow cytometry	FACS	Cells are prepared accordingly for high-throughput flow cytometry, and gated based on phenotype of interest; allows for investigation of multiple phenotypes in complex samples	10^5^ – 10^6^ cells	single-cell	Yes	Zahedi et al., 2020
Western blot		Protein is isolated from sample, separated by weight and probed for on gel with specific antibodies			No	Egelhofer et al., 2011

## Different Quiescence Model Systems for Studying Histone Marks

### Yeast Models of Quiescence

Multiple model systems have been used to study the molecular mechanisms of quiescence including yeast, mouse and human cells (Table 1), all of which have different genomes, limiting our ability to make direct comparisons about histone modifications in specific genomic regions. Further, the signals that induce quiescence, and the quiescent state achieved in these model systems differs, which may contribute to differences in the levels of specific histone modifications (Valcourt et al., 2012; Coller, 2019a). Among these model systems, each has advantages and disadvantages, for instance, budding yeast in a haploid state contain only one copy each of the major core histone genes (Eriksson et al., 2012). In haploid yeast, it is possible to alter a single amino acid to test the importance of a specific histone PTM, thus making yeast a particularly attractive model system for such studies.

Budding yeast, such as the well-studied strain *Saccharomyces cerevisiae (S. cerevisiae)*, participate in both symmetric mitotic cell divisions during the budding process, and meiotic cell divisions during yeast sporulation (Neiman, 2011). All microorganisms, including yeast, spend a majority of their life-cycle in a quiescent state due to a lack of resources in their natural environment (De Virgilio, 2012; Sagot and Laporte, 2019b). Diploid yeast cells can enter a quiescent state in response to nutrient depletion, stress, and even cell wall damage (Miles et al., 2019). *Saccharomyces cerevisiae* initiate quiescence following the exhaustion of nutrients, especially glucose, and have been widely used to study quiescence. Quiescent yeast share some similarities to the quiescent state of mammals (Gray et al., 2004; Dhawan and Laxman, 2015; Miles et al., 2021). When grown in the laboratory, yeast consume glucose present in their growth medium and when available nutrients have been depleted, the yeast undergo a change termed diauxic shift (Chu and Barnes, 2016) as their metabolic profiles transition from fermentation to respiration, resulting in a decreased growth rate. When no other carbon sources are readily available, the yeast enter stationary phase or quiescence (Galdieri et al., 2010). By fractionating the cells based on differing densities, the non-proliferating stationary phase yeast have been separated into a denser population that is long-lived, and a less dense subpopulation that has been termed “non-quiescent” (Allen et al., 2006). Differences in the accumulation of trehalose and lipids may contribute to the different densities of these populations (Sagot and Laporte, 2019b). Fractionation protocols that purify quiescent yeast have allowed for comparisons of histone modifications in quiescent and proliferative yeast samples (Mews et al., 2014). In budding yeast that initiate quiescence following nutrient exhaustion, there is a genomewide shift in gene expression, with transcriptional repression of genes involved in growth and proliferation including ribosomal genes (Werner-Washburne et al., 1996; Gray et al., 2004; Radonjic et al., 2005; McKnight et al., 2015).

Yeast can also form spores, which can serve as another model for quiescence. When diploid yeast cells undergo meiosis, the meiotic products can differentiate into dormant spores during the process of sporogenesis (Greig, 2009; Duina et al., 2014). The state achieved in dormant microbial spores shares some similarities to the quiescent state achieved by nutrient depletion in *S. cerevisiae* (Greig, 2009; Duina et al., 2014). For instance, spore formation, like quiescence, is reversible as spores germinate to form haploid cells when exposed to nutrients. Spores are distinguished from a quiescent state because quiescent cells maintain some metabolic capacity, maintain membrane potential and do not undergo a morphological differentiation (Rittershaus et al., 2013). Spore formation is characterized by a dramatic decrease in global transcription levels (Xu et al., 2012). As described below, both nutrient limitation and spore formation have been used as models to probe the role of histone PTMs in quiescence in *S. cerevisiae*.

Fission yeast like *Schizosaccharomyces pombe (S. pombe)* are also an excellent model for quiescence (Su et al., 1996). During meiosis in fission yeast, asymmetric division takes place in which inheritance of the mother cell’s components give rise to four different, unique daughter cells (Higuchi-Sanabria et al., 2014). Removing nitrogen from *S. pombe* causes the yeast to mate with yeast of the opposite mating type followed by replication through meiosis (Freese et al., 1982). However, if there is only one mating type of yeast in the population, the fission yeast arrest in G1-phase and enter quiescence (Nurse and Bissett, 1981; Gangloff et al., 2017). These nitrogen-deprived fission yeast can remain viable for months. Quiescent fission yeast cells are metabolically active, engage stress-responsive signaling and are efficient in DNA damage repair (Su et al., 1996; Mochida and Yanagida, 2006; Ben Hassine and Arcangioli, 2009; Marguerat et al., 2012; Gangloff and Arcangioli, 2017). When fission yeast enter a state of quiescence as non-dividing spores, a gene regulatory program is activated that includes upregulation of genes needed to adapt to the quiescent state (Su et al., 1996; Sajiki et al., 2009; Takeda and Yanagida, 2010), the nucleus undergoes changes in chromatin compaction, and histone modifications are altered (Neiman, 2011). For all of these reasons, fission yeast represents a valuable model system for studying epigenetic changes with quiescence.

### Fibroblast Models of Quiescence

In multicellular organisms, there are multiple different types of quiescent cells, such as quiescent fibroblasts, immune cells, and stem cells, that serve as model systems for the study of quiescence at the molecular level (Mitra et al., 2018a). Fibroblasts, which are normally quiescent *in vivo*, contribute to the physical form and biomechanics of tissue by secreting growth factors and extracellular matrix proteins. Fibroblasts are organizers of the wound healing process as they can proliferate and replenish dead cells at the wound site and secrete extracellular matrix proteins that contribute to the formation of granulation tissue and scars (Lynch and Watt, 2018). Fibroblasts isolated from different tissues such as skin or lung are relatively easy to culture, and quiescence can easily be achieved by serum starvation, contact inhibition, or loss of adhesion (Coller et al., 2006; Mitra et al., 2018a). When fibroblasts enter a quiescent state, there is a dramatic change in gene expression in which a large fraction of the genome is differentially regulated (Coller et al., 2006; Suh et al., 2012; Mitra et al., 2018b). This change in gene expression is accompanied by significant changes in the abundance and activity of microRNAs (Suh et al., 2012; Johnson et al., 2017), transcript decay rates (Johnson et al., 2017; Mitra et al., 2018b), splicing (Mitra et al., 2018b), and the use of proximal versus distal polyadenylation sites (Mitra et al., 2018b). Fibroblasts are a heterogeneous population of cells. They can be isolated from different locations within the skin including hair follicles, and locations within the dermal layer, such as the papillary and reticular dermis (Sorrell and Caplan, 2004), and they differ based on their location within tissue (Sorrell and Caplan, 2004; Lynch and Watt, 2018). Further, fibroblasts isolated from skin from different anatomical sites have distinct and characteristic transcriptional programs that include extracellular matrix synthesis, lipid metabolism and signaling pathways (Chang et al., 2002). Single-cell sequencing data has shed light on the heterogeneity of fibroblasts (Muhl et al., 2020). While the tissue of origin represents an important contributing factor to the differences among fibroblasts, single cell sequencing has also shown intra-organ heterogeneity (Muhl et al., 2020). Different fibroblast subpopulations have distinct characteristics and the contributions of each fibroblast population to physiology is being actively elucidated (Muhl et al., 2020).

### Stem Cell Models of Quiescence

Adult stem cells are another widely used quiescence model. Stem cells have been used to study quiescence in the context of the tissue-specific niche in which they are located. Many types of stem cells are largely quiescent unless activated to proliferate and differentiate in order to maintain tissue homeostasis and tissue regeneration (Li and Bhatia, 2011; Cheung and Rando, 2013; Coller, 2019b; Urbán and Cheung, 2021). Dysregulation or loss of stem cell quiescence can result in depletion of a stem cell pool, which can impede tissue regeneration (Cheung and Rando, 2013). Cellular quiescence has been studied in different types of adult stem cells such as hair follicle stem cells (HFSCs) within the skin (Lien et al., 2011; Lee et al., 2016; Rodriguez and Nguyen, 2018), hematopoietic stem cells (HSCs) from bone marrow (Nakamura-Ishizu et al., 2014), neural stem cells (NSCs) in the brain (Basak et al., 2018), and skeletal muscle stem cells (MuSCs) (Fukada et al., 2007). In the skin, the hair follicles that anchor hair to the skin progress through a cycle. During the anagen phase, there is rapid cell proliferation and growth of a new hair follicle. In the catagen phase that follows, the hair stops growing and detaches from the blood supply. Finally, in the telogen phase or resting phase, a new hair grows beneath the existing hair. Quiescent, non-proliferative hair follicle stem cells, which can be identified with cell surface markers including CD34 and CD49 (Garza et al., 2011), reside within a portion of the hair follicle called the bulge during the hair follicle’s resting stage, the telogen phase. During the transition from telogen to anagen, HFSCs are activated, exit the bulge and proliferate downward, creating a trail of rapidly proliferating cells (Nowak et al., 2008). These proliferating cells terminally differentiate to give rise to cells that form the new hair shaft and its channel. Hair follicle stem cells have been an important model system for understanding quiescence, including the epigenetics of quiescence.

Another important model system for understanding quiescence is the hematopoietic stem cell (HSC) compartment. HSCs are the stem cells that give rise to other blood cells including both myeloid and lymphoid lineages. In adult animals, hematopoiesis occurs in the bone marrow and the stem cells are only a small fraction of all of the cells present. The HSCs with the greatest capacity for self-renewal in the mouse bone marrow are quiescent HSCs (Wilson et al., 2008; Tesio et al., 2015) which are long-term label retaining and are in a deeply quiescent state (Foudi et al., 2009; Tesio et al., 2015). They are reported to divide only 5 times per lifetime (Foudi et al., 2009; Tesio et al., 2015). In response to infection or chemotherapy, these cells enter the cell cycle and start to proliferate to replenish damaged or lost cells (Wilson et al., 2008; Tesio et al., 2015). HSCs can be identified and isolated from surrounding cells by combinations of cell surface markers, including the presence of CD34 and an absence of markers for specific cell lineages (Sieburg et al., 2006; Dykstra et al., 2007; Kent et al., 2007).

Neural stem cells (NSCs) are present in the developing brain where they generate neurons (Ma et al., 2009). In adult animals, specific regions in the brain contain NSCs that have the capacity to proliferate and generate new neurons, thereby allowing adults to learn and acquire new skills, for instance, the ability to smell new odors (Ma et al., 2009; Obernier et al., 2018; Kalamakis et al., 2019). Like quiescent HFSCs, NSCs *in vivo* are thought to be slowly dividing and can be identified based on their label retention (Ma et al., 2009). These NSCs are depleted as organisms age (Obernier et al., 2018), and recent studies in single cells have shown an increase in quiescent NSCs in older mice compared with younger mice (Kalamakis et al., 2019). Markers for NSCs include expression of glial fibrillary acidic protein and glycoprotein CD133, along with an absence of differentiated cell markers (Ma et al., 2009).

Finally, muscle stem cells (MuSCs), or muscle satellite cells, represent another valuable quiescence model as MuSCs are mostly quiescent in uninjured tissue (Cheung et al., 2012). Muscle stem cells are crucial for the process of regenerating skeletal muscle (Boonsanay et al., 2016). Upon muscle injury, quiescent satellite cells are activated to re-enter the cell cycle and proliferate (Yin et al., 2013). The proliferating progeny of the muscle satellite cells can differentiate into myotubes that form muscle fibers (Dumont et al., 2015; Boonsanay et al., 2016). Other MuSCs return to quiescence and are available to assist in the repair of subsequent muscle injury events (Yin et al., 2013). *In vivo* analysis of MuSCs has revealed that MuSCs are primed for activation (van Velthoven et al., 2017). The quiescent state of MuSCs has been reported to be an “idling” state for stem cells because widespread, low-level transcription was observed and hypothesized to serve as a means to ensure that the transcription machinery is ready to respond when required (van Velthoven et al., 2017).

Stem cells in these different models are normally found in a quiescent state that is maintained by specific niches that are tightly regulated by intrinsic and extrinsic factors (So and Cheung, 2018). Within these complex niches and diverse tissue compartments, quiescent stem cells are identified by their low RNA content and lack of proliferative markers (Fukada et al., 2007). In many cases, quiescent stem cells can be isolated from the tissue of interest by monitoring the presence and levels of cell-surface markers with fluorescence-activated cell sorting (FACS), and label-incorporation assays that identify cells that haven’t divided for an extended time (Cheung and Rando, 2013; Nakamura-Ishizu et al., 2014). While this general pattern of quiescent stem cells is reported in multiple tissues, it is important to note that not all stem cells are quiescent, and in some tissues, stem cells are proliferative (Barker et al., 2007).

## H3 Methylation With Quiescence

Histone H3 has more potential methylation sites than any other histone in the histone octamer. Lysine residues 4, 9, 27, and 36 within the tail region of H3 are the most frequently methylated amino acids (Hyun et al., 2017; Jambhekar et al., 2019). In addition, H3K79, a lysine located within the globular domain of the histone, rather than on the tail, can also be methylated (Farooq et al., 2016). Each of the H3 lysines can be mono- (me), di- (me2), or tri-methylated (me3) by histone methyltransferases (writers) (Husmann and Gozani, 2019), and the methylation marks can be removed by histone demethylases (erasers). Histone methyltransferases, with the exception of DOT1L, contain a Suppressor of variegation, Enhancer of zeste, and Trithorax (SET) domain that catalyzes methylation of the lysine ε-amino group (DesJarlais and Tummino, 2016). Methylations of different histone lysines have characteristic deposition patterns that support a possible role for them in establishing different types of chromatin either by modulating the accessibility of the DNA to proteins, or by serving as a binding site for readers that act as effectors. Below we discuss the evidence that each of these marks plays a role individually and in combination in quiescence. Many of these marks have been investigated individually, but not in combination, and thus in many instances, our understanding of how they contribute to a histone code is limited.

### H3K4 Methylation

H3K4me1/me2/me3 are usually associated with gene activation (Jambhekar et al., 2019). H3K4me1, me2, and me3 methylation marks are enriched in enhancers, the 5’ ends of genes, and promoters, respectively (Hyun et al., 2017; Jambhekar et al., 2019). Methylation of H3K4 is associated with gene activation and the presence and absence of H3K4 methylation marks provides insight into the genome-wide patterns of active and inactive genes, respectively. Changes in genome-wide H3K4 methylation patterns with quiescence provide insights into gene activation and repression with quiescence. Whether these changes are functionally important for the changes in gene expression with quiescence, or are only correlated with quiescence, and whether H3K4 marks act alone or in combination with other marks or effector proteins, is the subject of active investigation in multiple model systems.

Young and colleagues investigated these questions in budding yeast *S. cerevisiae* by inducing the yeast into quiescence by glucose depletion (diauxic shift) and analyzing the yeast at 7 and 14 days. Overall levels of H3K4me2 were similar between log (proliferating cells) and stationary phase cells containing a mixture of quiescent and non-quiescent cells (Table 3) (Young et al., 2017). Levels of H3K4me3 decreased about 50% in quiescent and non-quiescent stationary phase cells compared with proliferating cells in these studies (Young et al., 2017). In another study focused on cell cycle entry from quiescence, *S. cerevisiae* were maintained in a nutrient depleted environment and then restimulated with complete medium (Mews et al., 2014). In this study, Mews and colleagues found that overall levels of H3K4me1, H3K4me2 and H3K4me3 were similar in log and a mixture of quiescent and non-quiescent stationary phase cells (Mews et al., 2014).

**TABLE 3:** Levels of histone marks in different quiescence systems.

Histone mark	General location	Generally associated with gene activation or repression?	Present within yeast	Up with quiescence	Down with quiescence
H3K4me2	Euchromatin	Gene activation	*Saccharomyces cerevisiae (S. cerevisiae) Schizosaccharomyces pombe (S. pombe)*		Decreased in sporulating yeast (Xu et al., 2012), mouse skin and HFSCs (Lee et al., 2016), and mouse B lymphocytes (Baxter et al., 2004)
H3K4me3	Euchromatin	Gene activation	*S. cerevisiae S. pombe*	Increase in adult mouse muscle stem cells (Liu et al., 2013)	Decrease in quiescent yeast (Xu et al., 2012; Young et al., 2017), sporulating yeast (Xu et al., 2012), bovine fibroblasts (Kallingappa et al., 2016), and in HFSCs (Kang et al., 2020)
H3K9me2	Heterochromatin	Gene repression	*S. pombe*	increased in muscle quiescent stem cells (Cheedipudi et al., 2015)	reduced in yeast (Oya et al., 2019) and bovine fibroblasts (Kallingappa et al., 2016)
H3K9me3	Heterochromatin	Gene repression	*S. pombe*	increased in MuSCs (Boonsanay et al., 2016)	reduced in mouse skin and HFSCs (Lee et al., 2016), yeast (Oya et al., 2019), and bovine fibroblasts (Kallingappa et al., 2016)
H3K9ac	Euchromatin	Gene activation	*S. cerevisiae S. pombe*		Global decrease in yeast (Mews et al., 2014)
H3K14ac		Gene activation	*S. cerevisiae S. pombe*		Decreased in muscle quiescent stem cells and yeast (Cheedipudi et al., 2015; Young et al., 2017)
H3K27me3	Heterochromatin	Gene repression			Decrease in skin and hair stem cells for growth (Kang et al., 2020), in mouse skin and HFSCs (Lee et al., 2016), and in bovine fibroblasts (Kallingappa et al., 2016)
H3K27ac	Euchromatin	Gene activation	*S. cerevisiae S. pombe*	Increase in neural stem cells (Martynoga et al., 2013)	
H3K36me1/me2/me3	Euchromatin	Gene activation	*S. cerevisiae S. pombe*	me3 increase in mouse bone marrow cells (Zhou et al., 2018)	reduced in bovine fibroblasts (Meng et al., 2020)
H3K79me1/me2	Heterochromatin		*S. cerevisiae S. pombe*		loss in yeast (Young et al., 2017)
H4K5ac	Euchromatin	Gene activation	*S. cerevisiae S. pombe*		Global decrease in yeast (Mews et al., 2014)
H4K16ac	Euchromatin	Gene activation	*S. cerevisiae S. pombe*		decreased in skeletal muscle stem cells and ESCs (Ryall et al., 2015; Kang et al., 2020)
H4K20me3	Heterochromatin	Gene repression	*S. pombe*	increased in primary human dermal fibroblasts (Evertts et al., 2013a), in MuSCs (Boonsanay et al., 2016)	

While H3K4me3 levels were similar or changed by 50% in the two studies comparing proliferating and stationary phase budding yeast, there was an observed difference in transcription rate between these two populations of cells. Levels of RNA Pol II CTD residues phospho-Ser5 and phospho-Ser2, which are indicators of transcriptional initiation and elongation, respectively, were high in day 3 post-diauxic cells, and subsequently decreased in day 5 post-diauxic cells (Young et al., 2017). In both proliferating and quiescent cells, H3K4me3 and RNA polymerase II were found at gene promoters, but the distribution of the H3K4me3 mark among promoters shifted with quiescence (Young et al., 2017). H3K4me3 was more abundant at the promoters of growth-associated genes in log phase yeast (Mews et al., 2014). Genes that retained the H3K4me3 mark and RNA Pol II at their promoters in quiescent yeast included genes responsible for stress response, protein catabolism, and energy production (Mews et al., 2014; Young et al., 2017). Thus, an association was observed between the presence of the H4K3me3 histone mark and activation of genes with quiescence, but further studies would be needed to determine whether this constitutes a “code” and if so, what the functional consequences of this mark are for quiescence.

Further studies have evaluated the functional importance of H3K4 methylation for quiescence in strains of *S. cerevisiae*. *S. cerevisiae* were genetically engineered so that lysine 4 of histone H3 was mutated to alanine. These mutant strains lost reproductive capacity over time, after being introduced into stationary phase, to a greater extent than wild-type yeast (Walter et al., 2014; Young et al., 2017). Further, in this mutant strain, the proportion of non-quiescent cells in the stationary phase increased (Walter et al., 2014; Young et al., 2017). These results indicate that methylation of H3K4 is required for the establishment or maintenance of a quiescent state initiated in response to nutrient depletion.

The effects of removal of H3K4 methylation marks (demethylation) have also been characterized in *S. cerevisiae* during spore production. In *S. cerevisiae* spores, which have very low transcription rates, there is an accumulation of the highly conserved H3K4 demethylase JARID1-family histone 2 (JHD2) during sporulation (Xu et al., 2012) (Figure 1). The spores of JHD2 mutant yeast strain, *jhd2*Δ, show a ∼2-fold increase in H3K4me3 and reduced levels of H3K4me1/me2 compared to wild-type spores, suggesting that absence of JHD2 leads to the conversion of H3K4me1/me2 to H3K4me3 (Xu et al., 2012). Studies with wild-type and *jhd2*Δ mutant strains indicated that JHD2 demethylases reduce intergenic transcription induced by H3K4me3 during spore differentiation, promoted protein coding gene transcription, and repressed nucleosome accumulation at transcription start sites (TSSs) of a large subset of ribosomal protein-coding genes. Mutants of *jhd2* exhibited precocious differentiation and the spores formed were sensitive to stress. Since JHD2 needs alpha-ketoglutarate from the TCA cycle for enzymatic function, it is possible that this family of proteins can sense carbon metabolism activity, which in turn regulates transcription in response to nutrient availability (Xu et al., 2012). These findings, taken together, suggest that H3K4me3 may be a mark that can transform information about nutrient availability into a complex pattern of gene expression that has distinct effects on protein coding genes, ribosomal genes and non-coding RNAs.

The role of H3K4 methylation has been studied in the context of quiescent mammalian cells as well. Kallingappa and colleagues compared chromatin states of proliferating bovine adult ear skin fibroblasts in the G1 phase of the cell cycle with chromatin composition of serum-starved, quiescent (G_0_) fibroblasts (Kallingappa et al., 2016). With fluorescence microscopy, quiescent nuclei were found to have a more relaxed or less compact chromatin state, and half the levels of H3K4me3 compared to G1 nuclei (Kallingappa et al., 2016). In mouse B cells, there was a dramatic shift in histone H3K4 methylation with quiescence, with much lower H3K4me2 levels in quiescent mouse B cells compared to cycling mouse B cells (Baxter et al., 2004). Reduction of H3K4me3 levels was also observed in quiescent mouse HSCs in late catagen stage in comparison to proliferating HSCs in early anagen stage (Lee et al., 2016; Kang et al., 2020). Based on ChIP-seq, the H3K4me3 signal generally decreased at transcription start sites and overall in quiescent compared with proliferating HSCs, although there was no clear correlation between H3K4me3 marks and gene expression changes with proliferation or quiescence (Lee et al., 2016). These findings suggest that H3K4 methylation may have other roles in addition to transcriptional regulation. Indeed, a recent article re-evaluating the role of histone-modifying enzymes argues that histone H3K4 methylation only has a minor role in transcriptional regulation (Rickels et al., 2017; Morgan and Shilatifard, 2020), but that H3K4 methylation has been associated with DNA recombination, repair, and replication (Daniel and Nussenzweig, 2012; Acquaviva et al., 2013; Kantidakis et al., 2016).

Similar results were observed in MuSCs where neither the number nor the identify of genes marked by H3K4me3 at their transcription start site changed in activated compared with quiescent stem cells, and quiescence-specific genes retained their H3K4me3 mark at their transcription start site even upon activation (Liu et al., 2013). The authors conclude that H3K4me3 marks genes for transcriptional activation but its presence is not sufficient to determine whether a gene will be expressed (Liu et al., 2013).

Evidence for the importance of H3K4 demethylation as a regulator of quiescence is derived from studies of Retinoblastoma binding protein 2 (RBP2), which can demethylate all the methylation states of H3K4 *in vivo* (Klose et al., 2007). *In vitro*, RBP2 can processively remove the me3 and me2 marks on H3K4 to return the histone to a singly methylated state, but it cannot demethylate the me1 mark (Klose et al., 2007). Hematopoietic stem cells (HSCs) and myeloid progenitors isolated from *Rbp2* knockout mice (*Rbp2*^–/–^) contain a higher proportion of cells exiting quiescence compared to WT. Further, in *Rbp2*^–^*^/^*^–^ cells, genes encoding cytokines were marked with higher levels of H3K4me3 and were expressed at higher levels, which could promote proliferation of the HSCs (Klose et al., 2007). In addition to the JmjC domain that is responsible for the demethylase activity, KDM5A/RBP2 also contains 2-3 plant homeodomain (PHD) domains (Klose et al., 2007). Binding of the PHD domain to unmodified H3 peptide activates the KDM5A/RBP2 catalytic activity and results in removal of methyl marks from histone H3K4me3 from a nearby nucleosome (Torres et al., 2015). By coupling the KDM5A/RBP2’s ability to read unmodified H3K4 with demethylation of nearby H3K4me3, results in a positive feedback loop that allows the spreading of a chromatin state of demethylated histones (Torres et al., 2015).

The data that are currently available suggest that methylation of H3K4 may play a role in aspects of quiescence, including potentially transcription, but whether this mark is a determinant of gene expression or simply associated with an activated promoter is not yet clear. H3K4 methylation could also contribute to quiescence through its roles in replication, repair or recombination. Establishing whether H3K4 methylation affects quiescence entry or maintenance through direct effects on the biomechanical properties of the DNA or through readers, whether it is part of a histone code, and how this affects functional aspects of quiescence will require additional studies.

### H3K9 Methylation

H3K9 methylation marks are primarily associated with gene repression in heterochromatin regions (Saksouk et al., 2015). H3K9me3 associates with regions of constitutive heterochromatin such as repeat regions of telomeres and centromeres (Saksouk et al., 2015). H3K9me3 is also deposited at some genomic regions in a tissue-specific manner and plays a role in cell identity (Ninova et al., 2019). Given the observation that in some systems, chromatin is more compact in quiescent than proliferating cells (Bridger et al., 2000; Evertts et al., 2013a; Guidi et al., 2015; Criscione et al., 2016; Swygert et al., 2019, 2021), H3K9 methylation is of particular interest as a potential regulator of chromatin compaction with quiescence.

In fission yeast, there is only one H3K9 methyltransferase, Clr4/Suv39H, which adds H3K9me1, me2, and me3 marks (Figure 1, Table 4). Inactivation of Clr4 resulted in viability similar to wild-type cells when nutrients were present, but reduced viability when quiescence was initiated following nitrogen starvation or glucose deprivation (Joh et al., 2016). This trend was also observed in yeast cells with a mutation that converted histone H3 lysine 9 to an alanine, suggesting that H3K9 methylation is important for survival during quiescence (Joh et al., 2016). Global ChIP-seq analysis showed that as fission yeast enter quiescence, the cells accumulate Clr4-dependent H3K9me2 and H3K9me3 marks at euchromatic genes whose transcriptional regulation has been shown to be important for establishing quiescence including genes involved in metabolism, ribosomal genes, cell cycle genes and stress response genes (Joh et al., 2016). In this study, a strong correlation was observed between genes with H3K9me2 marks and the set of genes repressed in G_0_ (Joh et al., 2016). The enrichment of H3K9 methylation marks in euchromatic gene regions upon quiescence entry required the small RNAs (sRNAs) associated with RNA interference (RNAi) factor Argonaute (Ago1). Quiescent yeast had a distinct profile of these sRNAs (Joh et al., 2016). Before H3K9 is deposited in these euchromatic regions in quiescent yeast, Ago1-associated sRNAs are expressed from these regions (Joh et al., 2016), and these sRNAs may serve as guides for the deposition of H3K9 methylation marks (Joh et al., 2016). This may reflect a mechanism for regulating the expression of specific genes as yeast enter quiescence. In contrast to euchromatic regions, the levels of H3K9me2 in constitutive heterochromatic regions decline early during quiescence (8h and 24h after starvation) (Oya et al., 2019). These findings support the possibility of a combined histone-RNA code that controls gene expression and viability during quiescence. Surprisingly, the authors observed relatively little overlap between the H3K9me3-enriched genes in quiescent cells and the genes that are repressed by Clr4 in quiescence based on RNA-seq analysis of wild-type and clr4-deleted yeast strains (Joh et al., 2016). This disconnect between the two genesets shows that additional studies will be needed to clearly determine whether H3K9 methylation plays a role in regulating transcription with quiescence, whether transcriptional regulation by H3K9 is crucial for viability in the quiescent state, and whether the effects of H3K9 in conjunction with short RNAs constitute part of a quiescence histone code.

**TABLE 4:** The relationship of histone writers with quiescence.

Histone writers (HGNC ids)	Histone mark	Model	Relationship with cell quiescence	References
SET1	H3K4me1/2/3	Fission Yeast	Downregulated in quiescence	Young et al., 2017
Clr4	H3K9me1/2/3	Fission Yeast	Upregulated in quiescence	Joh et al., 2016
Suv39h1	H3K9me2/3	Mouse Primary Keratinocytes	Upregulated in quiescence	Lee et al., 2016
Suv39h2	H3K9me2/3	Mouse Primary Keratinocytes	Upregulated in quiescence	Lee et al., 2016
PRDM2	H3K9me2	Mouse Myoblasts	Upregulated in quiescence	Cheedipudi et al., 2015
EZH2	H3K27me2/3	Mouse Primary Keratinocytes	Upregulated in quiescence	Lee et al., 2016
NSD1/2/3	H3K36me1/2	Murine Adult Hematopoietic Stem Cells	Downregulated in quiescence	Zhou et al., 2018
SETD2	H3K36me3	Murine Adult Hematopoietic Stem Cells	Upregulated in quiescence	Zhou et al., 2018
MOF	H4K16ac	Human Embryonic Stem Cells	Downregulated in quiescence	Khoa et al., 2020
Suv4-20h1	H4K20me2	Mouse Skeletal Muscle Stem Cells	Upregulated in quiescence	Boonsanay et al., 2016
		Primary Human Dermal Fibroblasts	Upregulated in quiescence	Evertts et al., 2013a
Suv4-20h2	H4K20me3	Primary Human Dermal Fibroblasts	Upregulated in quiescence	Evertts et al., 2013a

Similar to the findings in fission yeast, in hair follicles, quiescent HFSCs that were isolated from the late catagen stage of the hair follicle cycle contained considerably lower levels of the H3K9me3 mark compared to proliferating HFSCs isolated from the early anagen stage of the hair follicle cycle (Lee et al., 2016; Kang et al., 2020) (Table 3). In contrast to other studies, Boonsanay et al. found no change in the levels of H3K9me3 between proliferating and quiescent mouse MuSCs (Boonsanay et al., 2016). This study did not measure the global distribution of H3K9 methyl marks, and thus the genomic regions where H3K9 methyl marks are found or how they are redistributed in proliferating and quiescent MuSCs is not known (Boonsanay et al., 2016).

Studies of fibroblasts have also revealed differences in the levels of H3K9 methyl marks with quiescence although the changes observed varies in fibroblasts from different sources. In adult ear skin fibroblasts, overall levels of H3K9me2 and me3 were roughly halved in quiescent cells compared to the same fibroblasts in the G1 phase of the cell cycle, while the levels of H3K9me1 were slightly elevated in quiescent relative to G1 cells (Kallingappa et al., 2016). In contrast, levels of H3K9me3 were modestly elevated in quiescent human dermal fibroblasts compared to proliferating fibroblasts (Evertts et al., 2013a), while levels of H3K9me2 and H3K9me1 were similar (Evertts et al., 2013a). In mouse B lymphocytes, the levels of H3K9me2 and me3 were lower in quiescent cells compared to activated cells (Baxter et al., 2004). Thus, in different quiescence model systems, changes in the levels of H3K9 methylation states are altered, but the specific changes reported have been different depending on the model system, and thus a consistent quiescence program of H3K9 methylation changes has not been observed.

In mammals, there are multiple methyltransferases that add methyl groups to histone H3 lysine 9 including the 17 members of the PRDM family of proteins (Steele-Perkins et al., 2001; Hyun et al., 2017). One of these members, PR domain-containing-2/Rb interacting zinc finger protein (PRDM2/RIZ) is expressed at high levels in quiescent mouse MuSCs *in vivo* (Cheedipudi et al., 2015) (Table 4). Knockdown and overexpression studies of PRDM2/RIZ indicated that in quiescent MuSCs, PRDM2/RIZ prevents lineage commitment and irreversible cell cycle arrest (Cheedipudi et al., 2015). Global analysis using ChIP coupled with DNA microarray (ChIP-Chip) showed that PRDM2 was associated with >4400 gene promoters in quiescent muscle cells that initiate quiescence in suspension culture, that is, loss of adhesion. Approximately 50% of these promoters were also marked with H3K9me2 (Cheedipudi et al., 2015). The PRDM2-associated genes were enriched for differentiation, cell cycle, and developmental regulators (Cheedipudi et al., 2015). The levels of H3K9 methylation marks (me1, me2, and me3) did not change overall upon knockdown of PRDM2 (Cheedipudi et al., 2015). However, H3K9me2 levels were reduced at the MyoG promoter in G_0_ cells, while PRDM2 knockdown cells showed reduced H3K9me2 at the same locus, suggesting that PRDM2 may add methyl groups to generate H3K9me2 at MyoG (Cheedipudi et al., 2015). Further, increased H3K14Ac was observed at the MyoG promoter upon PRDM2 knockdown, supporting a role for PRDM2 in regulating the expression of MyoG, a critical molecule for muscle differentiation (Cheedipudi et al., 2015). These findings support a model in which PRDM2 activation in G_0_ ensures that two distinct possible outcomes—myogenesis and cell cycle progression—are poised for reactivation. The findings implicate H3K9 methylation, possibly in combination with other histone marks, in muscle stem cell quiescence and renewal (Cheedipudi et al., 2015).

Thus, while quiescence is generally associated with a reduction in transcription and number of active genes based on H3K4 methylation status as well as other markers, surprisingly in most, but not all, studies, there was not an increase in the H3K9me2 and H3K9me3 marks that might be expected. H3K9me3 is associated with constitutive heterochromatin (Saksouk et al., 2015) and it is possible that the increase in heterochromatin with quiescence reflects an increase in facultative heterochromatin. Nevertheless, even though H3K9me2/3 are not consistently observed to increase with quiescence, in yeast, there is evidence that these marks may be functionally important for the viability of quiescent cells as perturbations that reduce their levels reduce viability of quiescent cells with little effect on cells in full nutrient conditions. The data taken together support a possible role for H3K9 marks as contributors to changes that ensure the viability of quiescent cells, but these effects may be mediated through mechanisms other than transcriptional changes.

### H3K27 Methylation

**FIGURE 2:**
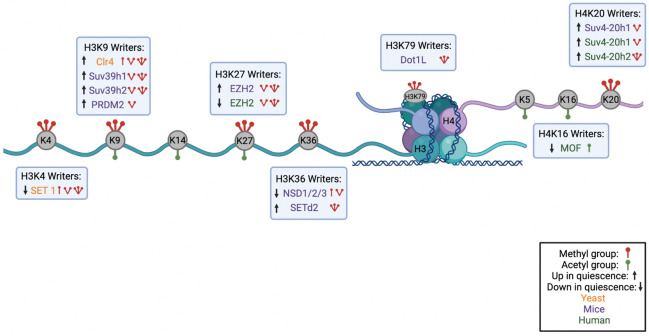
Summary of Histone Modifications on H3 and H4 Tail with their Corresponding Writer Enzymes. A schematic showing the nucleosome structure with H3 and H4 histone cores and corresponding N-terminal tails. Lysines available for methylation or acetylation are in gray with red icons indicating methyl groups and green icons indicating acetyl groups. Under or above each histone mark are the corresponding writer enzymes for each lysine with an indication of the number of methyl groups or acetyl groups each writer deposits. Writers found in yeast are shown in orange, in mouse models in purple, and in humans in green. Up or down arrows indicate whether the writer is upregulated or downregulated in quiescence (Figure made in BioRender).

The H3K27me3 mark is found in multiple model organisms including *Arabidopsis*, *Drosophila*, worms, and the filamentous fungus *Neurospora crassa*, but not yeast (Jamieson et al., 2013). H3K27me3 is a reversible mark of facultative heterochromatin, chromatin that can become compact or open depending upon the circumstance and is not repetitive (Trojer and Reinberg, 2007). H3K27me3 decorates genes that are developmentally regulated and are switched on and off depending upon the stage of development (Jambhekar et al., 2019). The Enhancer of Zeste 1 (EZH1) and EZH2 histone lysine methyltransferases trimethylate H3K27, and are constituents of Polycomb Repressive Complex 2 (PRC2) (Margueron and Reinberg, 2011) (Figures 1, 2, Table 4), while PRC1 stabilizes PRC2 binding to H3K27 and catalyzes monoubiquitination of histone H2A lysine 119 (Margueron and Reinberg, 2011). This ubiquitination mark represses transcription (Lavarone et al., 2019) and promotes chromatin compaction (Wiles and Selker, 2017; Lavarone et al., 2019). The Embryonic Ectoderm Development (EED) protein subunit of the PRC2 complex binds to H3K27me3 and this interaction increases the methyltransferase activity of the complex, resulting in a positive feedback loop that establishes zones of repressed chromatin (Margueron et al., 2009; DesJarlais and Tummino, 2016).

In some studies, H3K27me3 levels were reduced with quiescence. In bovine fibroblasts, which exhibit more open chromatin with quiescence, H3K27me3 levels were reduced by approximately a half when the fibroblasts entered quiescence in response to serum starvation (Kallingappa et al., 2016). Similarly, protein levels of the members of PRC2—EZH2, EED, and Suppressor of Zeste 12 (SUZ12)—were reduced by about half in G_0_ nuclei (Kallingappa et al., 2016). Proteins in the PRC1 polycomb complex were also reduced in quiescent bovine fibroblasts, specifically Polyhomeotic Homolog (PHC1) and Ring Finger Protein 2 (RING2) (Kallingappa et al., 2016). H3K27me3 levels are also reduced in quiescent mouse B lymphocytes compared to activated and cycling B lymphocytes (Baxter et al., 2004).

In contrast, murine chondrocytes (cartilage cells) that entered into a quiescent state by the application of physiological hydrostatic pressure had lower levels of H3K9me3 and higher levels of H3K27me3 compared to control cells that were not subjected to hydraulic pressure (Maki et al., 2021). In primary human dermal fibroblasts, a mass spectrometry-based analysis of histone post-translational modifications revealed higher levels of H3K27me3 in contact-inhibited, quiescent fibroblasts than proliferating fibroblasts (Evertts et al., 2013a). Further dissection of the H3K27me3 signal revealed that levels were particularly high when H3K27me2 was found in combination with H3K36me2 or if H3K27me3 was found on the same histone tail as H3K36me1 and H3K36me2 (Evertts et al., 2013a). This pattern of histone modifications may reflect the fact that H3K36me3 can inhibit the ability of PRC2 to methylate H3K27 (Schmitges et al., 2011; Yuan et al., 2011).

H3K27me3 levels have been observed to be reduced in multiple quiescence model systems involving stem cells. Quiescent mouse HFSCs isolated in the late catagen (quiescent) stage experienced a global decrease in the levels of H3K27me3 along with a decrease in H3K4me3 and H3K9me3, as mentioned in the sections above, compared to cycling HFSCs in early-anagen stage. This hypomethylation of quiescent HFSCs was confirmed using immunofluorescence, western blotting, and ChIP-seq methods (Lee et al., 2016). The levels of H3K27me3 were reduced in 64% of promoters with quiescence in HFSCs (Lee et al., 2016). Surprisingly, the changes in the levels of histone methylation marks, overall, did not correlate with changes in the levels of transcripts for the associated genes in quiescent versus proliferating hair follicle cells (Lee et al., 2016). There were exceptions: a larger than expected fraction of genes highly expressed at all hair cycle stages in the bulge had an increase in the levels of H3K27me3 and a set of genes defined as cell cycle regulators and tumor suppressors had almost no H3K27me3 in quiescent and proliferating HFSCs (Lee et al., 2016). To explore a possible role for decreased histone methylation levels during catagen, the bone morphogenetic protein (BMP) signal which normally maintains quiescence *in vivo* was inhibited, resulting in elevated levels of H3 K4/K9/K27 me3 in quiescent bulge HFSCs (Lee et al., 2016). These findings demonstrate that quiescent HFSCs in the bulge require active BMP signaling in order to maintain a hypomethylated H3 state (Lee et al., 2016). When keratinocytes, skin epithelial cells, were serum-starved in culture, transcript levels of several histone methyltransferases including EZH2 (forms H3K27me3), SUV39H1 and SUV39H2 (H3K9me3), decreased (Lee et al., 2016), while transcript levels of multiple histone demethylases increased (Lee et al., 2016). The latter include JMJD2a which catalyzes demethylation of histone H3 lysines 9 and 36 (Kim T.D. et al., 2012), UTX which demethylates H3K27me3 (Tang et al., 2017), and JARID1, which acts as a demethylase for H3K4me3 and H3K4me2 (Lee et al., 2016). After chemically inhibiting demethylases specific to the K4, K9, and K27 me3 marks with a cocktail applied to the mouse’s skin, cells at the catagen stage failed to generate new hair follicles in the following hair cycle. Thus, the reduction in the H3K4/K9/K27 me3 levels observed in quiescent HFSCs was necessary for the ability of quiescent cells to re-enter the cell cycle (Lee et al., 2016). While these data support the importance of histone demethylation for the quiescent state of HFSCs, it remains unclear whether one, two or all of these marks was required for a functional state, whether these marks maintain chromatin structure or serve as binding sites for effectors, and whether the absence of the methylation marks affected the activity of other marks such as acetylation marks.

Three different studies have investigated the role of H3K27 methylation in quiescent mouse MuSCs (Liu et al., 2013; Cheedipudi et al., 2015; Boonsanay et al., 2016). Liu et al. found that H3K27me3 levels were low in quiescent MuSCs and dramatically increased in activated stem cells (Liu et al., 2013) (Table 3). The transcription start sites of genes expressed at high levels in quiescent stem cells were marked with H3K4me3, but not H3K27me3 (Liu et al., 2013). The 2,019 genes that were marked by H3K27me3 at their transcription start site in quiescent stem cells displayed very low expression levels (Liu et al., 2013). Upon activation, there was a dramatic increase in H3K27me3 in the gene body and intergenic regions (Liu et al., 2013). Boonsanay and colleagues, in contrast, reported that the levels of H3K27me3 were not different between quiescent and proliferating MuSCs (Boonsanay et al., 2016).

Both Boonsanay et al. and Liu et al. discovered changes in the levels of writers and erasers of H3K27me3 as quiescent mouse MuSCs were activated. Liu and colleagues reported that the increase in H3K27me3 marks with activation was associated with higher transcript levels of Ezh2 and lower levels of the demethylase Jmjd3 (Liu et al., 2013). Boonsanay and colleagues also found higher levels of PRC2-Ezh2 in proliferating MuSCs and higher levels of PRC2-Ezh1 in the quiescent MuSCs (Boonsanay et al., 2016). These findings would be consistent with a study by Margueron and colleagues that showed that Ezh2 is more closely associated with proliferation, while Ezh1 is more abundant in non-proliferative adult tissues (Margueron et al., 2008). Margueron and colleagues discovered that PRC2-Ezh2 effectively catalyzes the formation of H3K27me2/3, while PRC2-Ezh1 directly represses transcription and compacts chromatin. Additional studies would be needed to determine whether the differences in expression of these methyltransferases results in changes in the genome-wide distribution of H3K27me2/3, whether there is a direct effect on transcription or chromosome compaction, or whether the shift in the relative abundance of these methyltransferases is functionally important for establishing, maintaining or reversing quiescence in MuSCs.

Cheedipudi and colleagues focused on H3K27me3 levels in MuSCs in the context of their co-existence with H3K4me3 and H3K9me2 and their co-regulation by the H3K9 methyltransferase PRDM2/RIZ. As described in the H3K9 methylation section above, PRDM2/RIZ, an H3K9 methyltransferase, is enriched in quiescent muscle cells *in vitro*, where it participates in stalling differentiation and cell cycle programs, while also maintaining genes involved in differentiation and proliferation poised for future activation (Cheedipudi et al., 2015). Cheedipudi and colleagues found that knocking down PRDM2 resulted in a reduction of H3K4me3 and H3K9me2, and higher levels of H3K27me3 at the cyclin A2 promoter in G_0_ MuSCs (Cheedipudi et al., 2015). They conclude that PRDM2 may block the deposition of H3K27me3 silencing marks at the cyclin A2 promoter in G_0_, thereby preserving the gene’s potential for reactivation (Cheedipudi et al., 2015). These studies highlight the combinatorial nature of the histone marks during the transition between proliferation and quiescence.

Thus, taken together, substantial changes in H3K27me3 have been observed in different models of quiescence. H3K27me3 may contribute to transcriptional repression in quiescent cells and may contribute to a poised quiescent state, characterized by an ability to re-enter the cell cycle. However, additional studies that probe the genomic location of H3K27me3 in proliferating and quiescent cells in different model system and their functional importance for quiescence will be needed to fully understand the specific role of H3K27 methylation individually or in combination with other marks in quiescence.

### H3K36 Methylation

H3K36 methylation marks are usually deposited across the entire gene body (Hyun et al., 2017; Jambhekar et al., 2019), and are associated with transcriptional activation, dosage compensation, transcriptional repression, and DNA repair (Wagner and Carpenter, 2012). In the study by Young and colleagues described above, quiescent budding yeast *S. cerevisiae* had similar levels of H3K36me2 and H3K36me3 marks compared to proliferating cells (Young et al., 2017). However, the levels of SET Domain containing 2 (Set2) which deposits all three (mono, di, and tri) methylation marks in budding and fission yeast (Morris et al., 2005; Wagner and Carpenter, 2012), were reduced as the cells entered a quiescent state. These findings indicated that H3K36me3 marks were deposited prior to quiescence entry, concomitant with the activity of RNA Pol II.

While yeast has only one H3K36 methyltransferase, humans have eight, including SETD2, the human ortholog of yeast Set2, that generates the H3K36me3 mark *in vivo*, and enzymes of the Nuclear Receptor-binding Set Domain (NSD) family (NSD1/2/3) that deposit H3K36me1 and H3K36me2 marks (Wagner and Carpenter, 2012) (Figure 2 and Table 4). The trimethylation of H3K36 by SETD2 is highly efficient on an unmethylated H3K36 compared to a H3K36me2 substrate (Husmann and Gozani, 2019). In *Setd2* conditional knockout mice with HSC-specific *Setd2* inactivation, there is a reduction of the number of HSCs in a quiescent state (Zhou et al., 2018). *Setd2* knockout HSCs had a reduced G_0_ fraction and increased G1 and S/G2/M phases of the cell cycle (Zhou et al., 2018). In addition, knockout HSCs also exhibited higher levels of apoptosis, reduced stem cell identity, increased differentiation toward progenitors and reduced multiple-lineage terminal differentiation potential (Zhou et al., 2018). *Setd2* knockout mice had mild bone marrow fibrosis, increased erythroid progenitors, but a decreased population of other bone marrow progenitors. As a result, the HSCs from *Setd2* conditional knockout mice were less able to repopulate the hematopoietic system upon transplantation (Zhou et al., 2018). *Setd2* knockout HSCs showed significant reduction of H3K36me3, increased levels of H3K36me1/me2, and increased Nsd1/2/3 at both transcript and protein levels (Zhou et al., 2018). The levels of H3K4me3, H3K79me2, and phosphorylation of Ser2 residue of RNA pol II, all of which are associated with transcriptional elongation, increased in *Setd2* knockout cells. Based on these studies, the authors proposed a model in which, in the absence of SETD2, NSD proteins promote the phosphorylation and elongation of RNA pol II on specific genes, leading to a loss of quiescence (Zhou et al., 2018). The findings support an important role for SETD2 in maintaining the quiescent state of HSCs (Zhou et al., 2018). It is unclear whether SETD2 plays this role through H3K36me3 or non-enzymatic functions such as by affecting cryptic transcription (Carvalho et al., 2013) or alternative splicing (Bhattacharya et al., 2021).

### H3K79 Methylation

In contrast to the previously discussed histone marks that are located on the histone H3 tail, the H3K79 methylation mark is located in the globular domain of H3 (Farooq et al., 2016). H3K79me2 and H3K79me3 are mainly found in the bodies of active genes and are associated with transcription elongation (Mueller et al., 2007). H3K79me2/3 can also maintain enhancer-promoter interactions at a subset of enhancers (Godfrey et al., 2019). H3K79 methylation has also been implicated in telomere silencing (Singer et al., 1998), recombination, DNA repair and cell cycle progression (Nguyen and Zhang, 2011). Monomethylation and trimethylation of H3K79 has been associated with gene activation and gene repression, respectively, in some studies (Barski et al., 2007). Disruptor of Telomere Silencing—Dot1 in yeast and DOT1L in humans—is the only enzyme responsible for methylation of H3K79 in *S. cerevisiae*, *Drosophila*, and humans as knockout of DOT1 in these organisms results in a loss of H3K79 methylation (van Leeuwen et al., 2002; Lee S. et al., 2018) (Figure 2 and Table 4). Both Dot1 and DOTL1 can catalyze mono-, di- and trimethylation (Frederiks et al., 2008). Dot1 is the only known non-SET domain-containing methyltransferase (Farooq et al., 2016). *S. cerevisiae* Dot1 does not methylate free histones, only histones in chromatin, in contrast to other histone methyltransferases (Lacoste et al., 2002; Lee S. et al., 2018). Yeast Dot1 also has histone chaperone activity and is particularly important for nucleosome dynamics and chromatin accessibility on transcribed regions of long genes (Lee S. et al., 2018).

After diauxic shift in *S. cerevisiae*, despite the general shut down of transcription, both the quiescent and non-quiescent yeast populations contained higher levels of the H3K79me3 mark than proliferating yeast cells (Young et al., 2017). However, the quiescent population had reduced H3K79me1 and H3K79me2 levels compared with the non-quiescent population (Young et al., 2017). Levels of Dot1 decreased by day 3 post-starvation and remained low throughout the time course that ended at day 7 (Young et al., 2017). The elevated levels of H3K79me3 and reduced levels of H3K79me1/2, while Dot1 was reduced could reflect that H3K79me3-containing nucleosomes were not turned over, or that demethylases were activated (Young et al., 2017). H3K79me3 was enriched in gene bodies in transcripts expressed specifically in growing cells or specifically in quiescent cells, with no redistribution to non-canonical locations in genes or intergenic regions (Young et al., 2017). Log and quiescent cells contained similar numbers of gene binding sites for H3K79me3 (Young et al., 2017). These marks were established soon after the shift to diauxic growth and then retained in quiescent cells even as transcription was reduced (Young et al., 2017). There was not a strong correlation between RNA polymerase II occupancy and H3K79me3 marks on specific genes in proliferating or quiescent cells (Young et al., 2017).

Mutant *S. cerevisiae* strains that are no longer able to methylate histone H3K4 or ubiquitinate histone H2B showed a shorter chronological lifespan, indicative of reduced ability to re-enter the cell cycle (Young et al., 2017). In contrast, a higher proportion of yeast entered quiescence upon glucose deprivation in H3K79 mutant, and the yeast with H3K79 mutations showed enhanced ability to re-enter the cell cycle (Young et al., 2017). The findings suggest that the presence of a lysine that can be methylated at H3K79 makes cells less able to re-enter the cell cycle after glucose deprivation, and surprisingly, makes the quiescent yeast less fit (Young et al., 2017).

The H3K79 mark has also been implicated in the quiescence of mouse HSCs. In a study described above, knockout of *Setd2*, a histone methyltransferase involved in the addition of the H3K36me3 mark, led to the loss of bone marrow reconstitution after transplantation, with the mouse HSCs exhibiting loss of quiescence, increased apoptosis, and inability to differentiate into multiple lineages (Zhou et al., 2018). When studying the changes at the histone level, it was found that knockout of *Setd2* led to increased expression of NSD1/2/3, which are also H3K36 methyltransferases that generate H3K36me1 and H3K36me2 marks (Zhou et al., 2018). Loss-of-function in SetD2, combined with gain-of-function NSD1/2/3, led to an increase in the H3K79me2 mark through recruitment of DOTL1, the H3K79 methyltransferase (Zhou et al., 2018). Together with increased recruitment of histone acetylase Brd4, which recruits the Super Elongation Complex (SEC), DOT1L enhanced RNA Polymerase II elongation and expression of target genes that promote apoptosis and differentiation of quiescent cells (Zhou et al., 2018). This resulted in increased expression of genes including Myc, as inhibiting Brd4 or Dot1l reduced markers of transcription elongation and Myc expression (Zhou et al., 2018). The findings support a model where multiple histone-modifying enzymes are co-regulated to affect the choice between quiescence and differentiation (Zhou et al., 2018).

These studies, taken together, suggest H3K79 methylation may play a role in quiescence through mechanisms that involve transcription initiation or elongation, or alternative processes. In yeast, this mark reduces the ability of nutrient-starved cells to proliferate when provided nutrients. In mammalian HSCs, data support H3K79 methylation as part of a complex regulatory code that affects transcription elongation and the fate of quiescent HSCs.

## H4 Methylation With Quiescence

While the N-terminus of H3 tails exit the nucleosome near DNA entry and exit sites, the tails of histone H4 (residues 1-20) stick out from the nucleosome’s face on either side (Ghoneim et al., 2021). DNA breathing dynamics are altered upon removal of either the H3 or H4 tail, suggesting that there is cross-talk between the H3 and H4 tails (Ghoneim et al., 2021). The region encompassing residues 16-23 of H4 contains a high density of arginine and lysine residues that constitutes a basic patch (Ghoneim et al., 2021). In crystal structures, this H4 basic patch interacts with DNA or the cluster of acidic residues called the H2A/H2B acidic patch on the same or nearby nucleosomes (Ghoneim et al., 2021). Histone H4 is mainly methylated at residue K20 in mammalian cells. The methylation of K20 requires the acidic patch, as H4K20 monomethylation is not detected in nucleosomes with a defective acidic patch (Ho et al., 2021). H4K20 methylation is conserved from fission yeast *S. pombe* to humans (Jorgensen et al., 2013). Histone H4K20me1 has been observed in budding yeast *S. cerevisiae* (Edwards et al., 2011).

H4K20me1 can act both as a repressive and an activating mark (Huang et al., 2021). On one hand, H4K20me1 causes chromatin condensation (Lu et al., 2008; Oda et al., 2009), and in mammalian cells, can recruit L3MBTL1, a H4K20me1 reader that can induce nucleosome compaction (Trojer et al., 2007). On the other hand, in the genomes of some mammalian cells, H4K20me1 correlates with gene activation (Barski et al., 2007; Wang et al., 2008; Cui et al., 2009). H4K20me3 has been implicated in heterochromatin maintenance (Kourmouli et al., 2004), and localizes to telomeres (Kourmouli et al., 2004; Schotta et al., 2004; Benetti et al., 2007; Mikkelsen et al., 2007; Marion et al., 2011) and repeated elements, including transposable elements (Martens et al., 2005; Mikkelsen et al., 2007; Montoya-Durango et al., 2009; Rhodes et al., 2016). In addition, H4K20me3 has also been discovered in zinc fingers (Nelson et al., 2016), and promoters of E2F responsive genes (Abbas et al., 2010), histones (Abbas et al., 2010), inflammatory genes (Stender et al., 2012), and ribosomal genes (Bierhoff et al., 2014).

A ChIP-seq study in murine ESCs found that H4K20me3 colocalizes with transcriptionally active marks like H3K4me3 and H3K36me3 (Xu and Kidder, 2018). This bivalent placement of H4K20me3 with activating histone marks suggest a potential combinatorial histone code for a “poised” state. These “poised” states have been suggested to allow for rapid activation of RNA pol II (Bernstein et al., 2006) during cell state transitions. Sims and colleagues report on another combinatorial code involving H4K20, but in conjunction with H3K9 (Sims et al., 2006). They found that H4K20me3 and H3K9me3 were both enriched in pericentric heterochromatin. H4K20me1 and H4K20me2 were found together with H3K9me1 and H3K9me2, respectively, but in different regions on chromosome arms (Sims et al., 2006). The authors further found that H4K20me1 and H3K9me1 were enriched in the same nucleosome (Sims et al., 2006). What role these bivalent marks play in quiescence is not known and requires additional exploration.

H4K20 methylation status is cell cycle dependent (Pesavento et al., 2008; Oda et al., 2009; Abbas et al., 2010; Centore et al., 2010; Adikes et al., 2020), at least in part because PR-Set-7, the enzyme that generates H4K20me1 (Fang et al., 2002; Nishioka et al., 2002b; Beck et al., 2012), is actively targeted for proteasome-mediated degradation in S phase (Rice et al., 2002; Julien and Herr, 2004; Jorgensen et al., 2007; Abbas et al., 2010; Tardat et al., 2010; Wu et al., 2010). Consistent with these previous reports, in primary human fibroblasts, unmodified H4K20 was most abundant in S phase, less abundant in G2/M cells, and present at low levels in G1 and cells that entered quiescence by contact inhibition for 14 days (Evertts et al., 2013a). H4K20me1 was present at low levels in S phase, increased in G2/M, and increased further in G1 (Evertts et al., 2013a). H4K20me3 represented a small fraction of all of the histones, and was strongly induced with quiescence compared with all other cell cycle phases (Evertts et al., 2013a) (Table 3).

Boonsanay and colleagues investigated the levels of different histone marks in quiescent mouse MuSCs, proliferating MuSCs and differentiated myotubes (Boonsanay et al., 2016). Quiescent MuSCs contained a lower level of H4K20me1, but comparable levels of H4K20me2 to that present in proliferating MuSCs. The levels of H4K20me3 were in the order: differentiated myotubes > quiescent MuSCs > proliferating MuSCs (Boonsanay et al., 2016) (Table 3). In contrast, H3K9me3 and H3K27me3 and euchromatin marker H3K9ac did not change between proliferating and quiescent MuSCs (Boonsanay et al., 2016). Without ChIP-seq data, the distribution of histone H4K20 methylation marks, and the extent of overlap with other histone marks in these different cells is not known.

In mammals, H4K20me1 is catalyzed by PR-SET-7 (Nishioka et al., 2002a) and the methyltransferases KMT5B/Suv4-20h1 and KMT5C/Suv4-20h2, and possibly KMT3E/SMYD3, generate H4K20me2 and H4K20me3 (Rice et al., 2002; Schotta et al., 2004, 2008) (Figures 1, 2, Table 4). Loss of both SUV4-20H enzymes leads to strongly elevated levels of H4K20me1 (Schotta et al., 2008). Electron micrographs of MuSC nuclei with inactivation of Suv4-20h1 revealed a reduction in condensed heterochromatin (Boonsanay et al., 2016). Loss of Suv4-20h1 lead to a reduced population of quiescent cells and increased population of differentiated cells expressing *MyoD* (Boonsanay et al., 2016). In Suv4-20h1-abrogated quiescent MuSCs, there was a reduction in H4K20me2 and nucleosome density, and an increase in H3K4me3, at the distal regulatory region (DRR) that controls *MyoD* expression (Boonsanay et al., 2016). This indicates that DRR is more accessible in Suv4-20h1 knockout quiescent cells and this may allow expression of differentiation-related gene, *MyoD*. Thus, Suv4-20h1 reduces expression from the *MyoD* locus, resulting in the maintenance and preservation of stem cells in a quiescent state (Boonsanay et al., 2016). Further research will be required to determine whether this is a role for H4K20 methylation exclusively in MuSCs, or whether similar changes occur in other stem cells as well.

The H4K20 monomethyltransferase Pr-set-7 was discovered to regulate NSC quiescence in *Drosophila* (Huang et al., 2021). Targeted DNA adenine methyltransferase identification (TaDa) was used to determine genomic loci where Pr-set7 binds. This analysis revealed Pr-set-7 binds to the promoter and transcriptional start sites of Wnt pathway coactivator earthbound1 (Ebd1) and cyclin-dependent kinase 1 (Cdk1) (Huang et al., 2021). The mRNA levels of Ebd1 and Cdk1 were depleted in *pr-set7* mutant brains (Huang et al., 2021). Thus, by increasing expression of Cdk1 and Ebd1 in NSCs, Pr-set7 modulates the cell cycle and Wnt signaling, and thereby promotes NSC reactivation from quiescence (Huang et al., 2021). In these studies, The presence or absence of H4K20me1 in *Ebd1* and *Cdk1* promoters was not determined in this study (Huang et al., 2021).

In primary human dermal fibroblasts, mass spectrometry-based analysis of histone tails revealed that the histone lysine that showed the strongest change in methylation levels with quiescence was H4K20 (Evertts et al., 2013a). H4K20me2, and especially H4K20me3, were highly induced in quiescent cells when compared with proliferating fibroblasts as well as the fibroblasts that were specifically in the G1 phase of the cell cycle and contained the same DNA content as quiescent fibroblasts (Evertts et al., 2013a). In contrast, H4K20me1 levels were reduced in quiescent compared with proliferating fibroblasts. Further knockdown of Suv4-20h1 and Suv4-20h2 methyltransferases that catalyze formation of di and tri-methylated H4K20 with both small hairpin RNAs and small interfering RNAs (siRNAs) resulted in less chromatin compaction, consistent with a potential role for H4K20 trimethylation in chromatin conformation (Evertts et al., 2013a). Knockdown of Suv4-20h2 specifically, with siRNAs, resulted in increased proliferation and more cells in S phase (Evertts et al., 2013a). These findings support a role for Suv4-20h2 in both the regulation of the quiescence-proliferation transition and chromatin compaction with quiescence. Whether there is a direct link between these two activities, and whether they are mediated through H4K20 methylation will require additional studies.

Altogether, these studies highlight the critical role of H4K20 methylation, and in particular, H4K20me3, in regulating the transition between quiescence and proliferation. The consistent upregulation of H3K20me3 in multiple different quiescent models indicates it might play a larger role in establishing the functional state of chromatin in quiescence and may regulate specific gene expression changes necessary for quiescence entry, exit, and maintenance. Given the available data, it is possible that the H4K20 methylation marks act in cooperation with other marks, for instance it has been detected as part of bivalent promoters in combination with H3K4me3 and H3K36me3 (Xu and Kidder, 2018), and as part of heterochromatin in combination with H3K9me3 (Schotta et al., 2004). Taken together, the data support the possibility that H4K20me3 represents one part of a combinatorial code that regulates quiescence.

## Histone Acetylation With Quiescence

### General Properties of Histone Acetylation

An array of conserved lysine residues is present in the N-terminal tails of the four histones forming the nucleosome and these positively charged lysines interact with the DNA and the negatively charged patch formed by H2A/H2B residues of the nearby nucleosome. The tail of histone H4 has the strongest effect on chromatin compaction, followed by the tail of histone H3, which is followed by the tails of histones H2A and H2B (Ghoneim et al., 2021). Histone acetylation weakens the interactions of tails with the DNA and the negative patch, thus making the chromatin more accessible to RNA Pol II (Park and Kim, 2020). In addition to affecting the structural state of the chromatin, acetylation can also affect the proteins bound to chromatin. Lysine acetylation marks on histone tails can be recognized by bromodomain-containing reader proteins (such as chromatin remodelers) that are generally associated with transcriptional activation (Agalioti et al., 2002; Barnes et al., 2019). The removal of acetyl groups by histone deacetylases can modulate the chromatin state to make the chromatin more compact. Further, in some cases, an individual lysine residue can be acetylated, methylated or ubiquitinated, and this establishes a potential for competition among these different lysine modifications.

The N-terminal tail of histone H3 is mainly acetylated at residues K9, K14, K18, and K23, while the corresponding region of H4 is acetylated at residues K5, K8, K12, and K16. Acetylation on H4K16 is particularly linked to unpacking of the chromatin and transcriptional activation—mainly due to diminished interactions between the H4 tail and the H2A/H2B acidic patch (Shogren-Knaak et al., 2006). The periodic nature of the amino acid spacing between the acetylatable lysines in the H3 and H4 tails suggests a potential for cooperative behavior among these amino acids (Strahl and Allis, 2000). In particular, this spacing has been noted to be reminiscent of the 3.6 residues per turn of an α-helix, raising the possibility that these acetyl marks may act as part of a combinatorial code that provides information about which proteins should bind to chromatin at a specific genomic region (Strahl and Allis, 2000).

The combined presence of histone acetyl marks and other histone modifications have been shown to result in defined outcomes (Winter et al., 2008; Zippo et al., 2009). As one example of this combinatorial effect, Bromodomain PHD Finger Transcription Factor (BPTF), an ATP-dependent chromatin remodeling protein, has increased affinity for histones with two different marks. The presence of H3K4me3 and H4K16ac together results in a 2-fold stronger BPTF binding affinity than either H3K4me3 or H4K16ac alone (Ruthenburg et al., 2011; Rando, 2012). This effect was not limited to H4K16ac as peptide microarray studies showed an increase in binding of PHD-Bromodomain constructs to histones when H3K4me3 was combined with any of multiple different H3 acetyl states (Fuchs et al., 2011; Rando, 2012). As another example, some proteins bind preferentially when a single epigenetic mark is found by itself, in the absence of another mark (Rando, 2012). This is exemplified by a PHD-Bromo domain chromatin regulator TRIpartite-Motif containing 24 (TRIM24) as this protein binds to H3K23ac to activate estrogen-responsive genes, but this binding is inhibited if H3K4 is also methylated (Tsai et al., 2010; Rando, 2012). Taken together, these reports suggest that a histone acetylation-dependent combinatorial histone code may encode information through the presence of individual histone marks, combinations of marks, their spacing, and the specific readers that recognize the marks. In the sections below, we focus on acetylation of histones H3 and H4 as these have been studied the most in relation to cellular quiescence.

### H3 Acetylation With Quiescence

In budding yeast *S. cerevisiae*, a global decrease in acetylation levels was observed after quiescence entry that parallels the repression of transcriptional activity in quiescence (McKnight et al., 2015). Upon entry into quiescence, the budding yeast cells have lower levels of histone H3 acetylation compared with log phase cells. Immunoblotting for histone H3K9ac, H3K23ac, and pan-acetyl histone H3 revealed a significant reduction in the levels of these marks in quiescent compared with proliferating yeast (McKnight et al., 2015). In a complementary set of studies, using mass spectrometry, Mews et al. showed that when quiescent yeast cells reentered the cell cycle upon nutrient replenishment, there was a burst of histone acetylation, specifically at H3K9 and H3K14, immediately upon cell cycle entry (Mews et al., 2014). In contrast, *de novo* histone methylation occurred at a later cell cycle stage after the acetylation burst. As compared to histone methylation, histone acetylation was more closely correlated with the transcriptional changes that took place soon after the cell cycle entry. Western blot analysis revealed that levels of acetylated histones H4K5ac, H4K8ac, and H3K9ac drop during quiescence and then robustly increase over a 240-min time course after nutrient refeeding. Using ChIP-seq, Mews and colleagues found that when yeast enter stationary phase, H3K9ac levels decreased at genes identified by microarrays as growth genes and increased at genes expressed when yeast initiated quiescence following nutrient exhaustion (Mews et al., 2014). After the nutrients were replenished, acetylation of H3K9 increased at growth genes and decreased at stress response genes (Mews et al., 2014). Histone acetylation rapidly responded to changes in metabolic state, while histone methylation levels were largely constant (Mews et al., 2014). Thus, *S. cerevisiae* undergo a dramatic increase in acetylation, and not methylation, when they re-enter the cell cycle from quiescence as nutrients are re-introduced (Mews et al., 2014; McKnight et al., 2015; Young et al., 2017).

The histone lysine deacetylase Rpd3 has been identified as an important regulator of quiescence entry and maintenance in *S. cerevisiae* in a study by McKnight and colleagues (McKnight et al., 2015). When the yeast entered quiescence, motifs associated with transcription factors inactivated by nutrient exhaustion were associated with more repressive chromatin structure including more nucleosomes positioned over the motif and lower levels of histone acetylation (McKnight et al., 2015). Motifs for transcriptional activators that function in stress conditions had more open chromatin structure in the quiescent cells (McKnight et al., 2015). A large fraction of transcription factors with binding motifs that exhibit changes in chromatin structure with quiescence function by recruiting Rdp3 lysine deacetylase (McKnight et al., 2015). Deleting Rpd3 did not affect growth in the log phase and Rdp3-deleted cells maintained high viability in log phase (McKnight et al., 2015). Rpd3-deficient cells arrest after glucose exhaustion similarly to wild-type cells. However, fewer quiescent cells are formed in an *rpd3*Δ mutant and the quiescent cells that are formed show reduced long-term survival (McKnight et al., 2015). Analysis of chromatin structure at gene promoters revealed that Rpd3 has a significant role in establishing the transcriptional profile of quiescent cells by affecting the density of histone H3 and the acetylation of histone H4 at the promoters of genes regulated with quiescence (McKnight et al., 2015). The findings, taken together, support histone lysine acetylation as an important regulator of gene expression and viability during quiescence in *S. cerevisiae* (McKnight et al., 2015).

Studies in mouse NIH 3T3 embryonic fibroblasts that were serum starved to induce quiescence and then restimulated with serum addition revealed changes in histone acetylation (Knosp et al., 1991). Using electrophoretic and fluorographic techniques, in 1991, Knosp et al. reported that addition of serum resulted in an increase in the acetylation rate of all core histones within 15 min (Knosp et al., 1991). The sharp increase in acetylation rate was followed by a gradual decline in acetylation rate that continued until 8 h post serum stimulation (Knosp et al., 1991). At 10-12 h after serum stimulation, there was a strong increase in the acetylation rate of all core histones, detected based on increased incorporation of tritium into histone H3 from labeled acetate, a methodology that doesn’t distinguish between acetylation of different lysine residues. This increase in acetylation was followed by an increase in histone synthesis (Knosp et al., 1991). The pattern differed among histones: H3 > H2A ∼ H2BB > H4 (Knosp et al., 1991). In response to 24 h of serum withdrawal, there was a decrease in DNA synthesis, histone synthesis and histone acetylation (Knosp et al., 1991).

In a more recent study, proliferating and quiescent primary human fibroblasts, were incubated in ^13^C-labeled acetate and the rate of histone lysine acetylation was monitored using mass spectrometry (Evertts et al., 2013b). The quiescent fibroblasts accumulated labeled acetylated histones more slowly than proliferating fibroblasts (Evertts et al., 2013b) and differential labeling rates were observed for the acetylation of H3K9, H3K14 and histone H4 (Evertts et al., 2013b). For histone H4, mass spectrometry was used to monitor the extent of acetylation of a peptide that contained lysines K5, K8, K12 and K16 was monitored. Levels of unmodified peptide, peptide with one acetyl group and peptides with two acetyl groups were determined (Evertts et al., 2013b). Incorporation of ^13^C in acetyl groups in quiescent cells was approximately half of that for proliferating cells. Even though the rate of acetylation was faster in proliferating fibroblasts, steady state levels of histone acetylation were similar in proliferating and quiescent fibroblasts (Evertts et al., 2013a, b).

### H4 Acetylation With Quiescence

In yeast, H4 acetylation correlates with transcriptional activation and plays an important role in chromatin decompaction (discussed in more detail in the later section). Using immunoblotting, the overall levels of H4K5ac and H4K8ac were found to decrease sharply during quiescence in budding yeast *S. cerevisiae*. This was followed by a rapid increase of these histone marks during cell cycle reentry when nutrients were reintroduced (Mews et al., 2014). Also using immunoblotting, a reduction in histone H4K12ac was observed in quiescent *S. cerevisiae* (McKnight et al., 2015). Similarly, by mass spectrometry and western blot analysis, two separate studies found that during stationary phase in *S. cerevisiae*, histone acetylation dropped dramatically at H4K5, H4K8, H4K12, and H4K16 compared to their levels in exponential growth phase (Sandmeier et al., 2002; Ngubo et al., 2011).

In mammals, reduced histone acetylation has been associated with a transition to quiescence in pluripotent cells (Khoa et al., 2020). Embryonic stem cells with pluripotent potential are characterized by high levels of histone acetylation, high chromatin accessibility and an active pluripotency transcriptional network. Mouse embryonic stem cells (ESCs) can exist in a proliferative, naïve ground state achieved by maintenance with a MAPK/ERK Kinase (MEK) inhibitor and a Glycogen Synthase Kinase 3 (GSK3) inhibitor (Boroviak et al., 2014). Deletion of histone acetyltransferase Males absent On the First (MOF), which acetylates histone H4 lysine 16 (Finley et al., 2018; Atlasi et al., 2019), resulted in quiescence in mouse ESCs (Figures 1, 2, Table 4). Among the > 200 histone post-translational modifications that were monitored in these MOF-deleted cells, only H4K16ac was significantly decreased (Khoa et al., 2020). Comparing RNA-seq gene expression data with global H4K16ac distribution obtained by ChIP-seq revealed that many genes associated with metabolic processes were regulated in a MOF-dependent manner, especially β-oxidation of fatty acids (Khoa et al., 2020). The degradation of fatty acids provided metabolites for oxidative phosphorylation and energy production in the proliferative state (Khoa et al., 2020). Inhibiting fatty acid oxidation was also sufficient to induce the proliferative ESCs into a quiescent state (Khoa et al., 2020). Taken together, these studies highlight an important role for histone H4 acetylation in regulating the proliferation-quiescence transition.

While addition of H4 acetyl groups is associated with the transition from quiescence to proliferation, the removal of H4 acetyl groups by Sitruins, a class of histone deacetylases (HDACs), has also been associated with the proliferation-quiescence transition (Ryall et al., 2015) (Figure 1). When mouse MuSCs exit quiescence to begin proliferating, there is a decrease in NAD^+^ levels and activity of NAD^+^-dependent sirtuin 1 (SIRTS), an HDAC (Michan and Sinclair, 2007). This reduction in SIRT1 activity results in increased H4K16 acetylation, activation of muscle gene transcription, premature myogenic differentiation, reduced myofiber size, impaired muscle regeneration, and derepression of muscle developmental genes (Ryall et al., 2015).

These studies taken together support a role for acetylation in modulating transcriptional activation at important genes in different quiescence models. Loss of acetylation marks at genes critical to cell cycle and cell growth define the functional state of the chromatin during quiescence. In yeast and MuSCs, the alterations in acetylation have been functionally linked to quiescence (McKnight et al., 2015; Ryall et al., 2015).

## RNA-Mediated Addition of Histone Marks

The mechanisms that dictate where histone marks are deposited are not well-understood. There is an emerging literature that suggests that in some instances, long non-coding RNAs (lncRNAs) can help to direct the deposition of histone epigenetic marks (Batista and Chang, 2013; Hanly et al., 2018). LncRNAs can act as “address-codes” by directing the writers of histone marks to specific chromosomal locations (Batista and Chang, 2013; Hanly et al., 2018). The deposited marks can then affect chromatin conformation, nucleosome positioning and transcription at nearby genes (Faghihi and Wahlestedt, 2009; Guttman et al., 2011). The lncRNA Xist, for example, interacts with polycomb repressive complexes PRC1 and PRC2, which are responsible for the addition of the H3K27me3 mark, and recruiting these complexes is one part of the process by which Xist induces transcriptional silencing of the X chromosomes in female mammals (Kohlmaier et al., 2004; Bousard et al., 2019). Another lncRNA termed HOTAIR represses the HoxD gene locus by recruiting PRC2 for H3K27me3 addition (Rinn et al., 2007). LncRNAs have been confirmed to play critical roles in the establishment and maintenance of heterochromatic regions (Deng et al., 2009).

LncRNAs including *H19* are emerging as critical regulators of quiescence and proliferation in HSCs (Venkatraman et al., 2013; Yildirim et al., 2013; Luo et al., 2015). The *H19-Igf2* locus is an imprinted region that affects organism growth (DeChiara et al., 1991). The differentially methylated region (DMR) upstream of *H19* is the imprinting control region that enforces the expression of *H19* from the maternal allele only while *Igf2* is expressed from the paternal allele (Bartolomei, 2009). Deletion of the maternal, but not the paternal, *H19* DMR in adult mouse HSCs resulted in a activation of quiescent HSCs and reduced function of the HSCs (Venkatraman et al., 2013). When maternal *H19* DMR was deleted, the *Igf2-Igf1r* pathway was activated, the FoxO3 transcription factor translocated from the nucleus to the cytoplasm, and quiescent HSCs were activated to proliferate, resulting in their exhaustion (Venkatraman et al., 2013).

**FIGURE 3:**
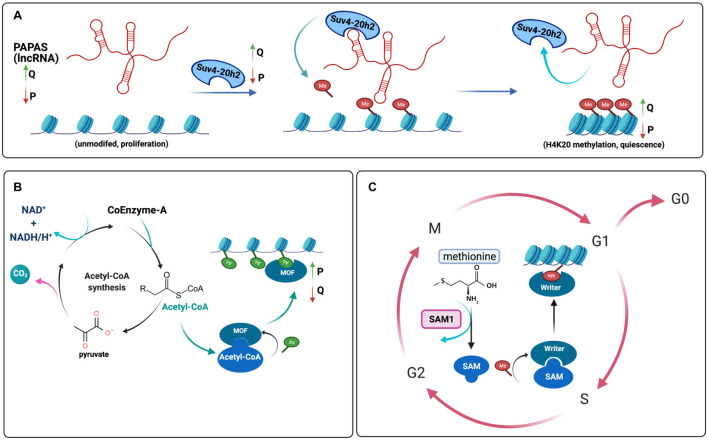
Histone Modifications and Metabolism | **(A)** During quiescence, the writer Suv420h2 adds methyl groups to H4K20 via mediation with the lncRNA PAPAS. PAPAS, Suv4-20h2, and H4K20 methylation levels all increase with quiescence (Q) compared to proliferation (P), as indicated by the arrows (Bierhoff et al., 2014). **(B)** Acetyl-CoA acts as an acetyl group donor for histone acetyltransferases, including MOF, which is responsible for H4K16 acetylation during proliferation (Khoa et al., 2020). **(C)** In fission yeast, synthesis of S-adenosylmethionine (SAM) from methionine with the help of S-Adenosylmethionine synthetase (Sam1) is implicated in histone methylation. SAM acts as a methyl donor for methyltransferases; thus, Sam1 is necessary for proper cell growth and proliferation, as well as quiescence maintenance (Hayashi et al., 2018) (Figure made in BioRender).

PAPAS lncRNAs have been studied in the context of cell quiescence (Figure 3A). PAPAS, or “promoter and pre-rRNA antisense,” is a heterogeneous group of lncRNAs that are generated when ribosomal DNA (rDNA) is transcribed in the antisense direction and localize to the pre-rRNA coding region and the rDNA promoter (Bierhoff et al., 2010). PAPAS interacts with Suv-20h2, the methyltransferase that generates H4K20me3, and mediates deposition of this mark at rDNA during quiescence (Bierhoff et al., 2010). PAPAS and H4K20me3 were found to be upregulated in quiescent breast cancer cell line MCF7 generated with an estrogen receptor antagonist (Bierhoff et al., 2014). PAPAS and H4K20me3 are also induced upon terminal differentiation of human colon cancer cells (Caco-2), and upon quiescence in C2C12 mouse myoblasts, and mouse fibroblast-like 3T3-L1 cells (Bierhoff et al., 2014). Knockdown of endogenous PAPAS with siRNAs in MEFs decreased H4K20me3 levels (Bierhoff et al., 2014). Gain- and loss-of-function experiments, RNA-protein binding assays, and chromatin accessibility analysis revealed that PAPAS guides Suv4-20h2 to nucleolar chromatin, reinforcing quiescence-dependent transcriptional repression of ribosomal RNAs through H4K20me3-dependent chromatin compaction (Bierhoff et al., 2014).

Intracisternal A particle (IAP)-specific lncRNAs were found to be involved in H4K20me3-mediated chromatin compaction at IAP retrotransposons (Bierhoff et al., 2014). At IAP elements, both H4K20me3 and Suv4-20h2 levels increased in serum-starved MEFs, and the IAP chromatin was more compact (Bierhoff et al., 2014). Knockdown of IAP lncRNA with locked nucleic acid technology resulted in upregulation of the IAP major 7 kb transcript, supporting a role for the lncRNA in causing repression of the IAP gene, while transfection of IAP lncRNA sequences that interact with Suv4-20h2 resulted in increased Suv4-20h2 and H4K20me3 at IAPs, with no change in H3K9me3 levels (Bierhoff et al., 2010). These studies suggest that lncRNAs may provide another dimensionality to the histone code by potentially providing an address code that directs histone marks to specific genomic positions.

## Histone Marks and Chromatin Conformation During Quiescence

Multiple studies have reported changes in chromatin compaction with quiescence with most, but not all studies (Kallingappa et al., 2016), reporting that when cells enter quiescence, their chromatin becomes more compact (Chiu and Baserga, 1975; Setterfield et al., 1983; Evertts et al., 2013a; Swygert et al., 2019). In *S. cerevisiae* budding yeast, quiescence induction was found to induce global changes in chromosomal organization (Rutledge et al., 2015) using a global chromosome conformation capture high-throughput sequencing technology called Hi-C. By monitoring the frequency of interloci interactions, the study revealed an increase in long range *cis* interactions and a decrease in short range interactions in quiescent yeast cells (Rutledge et al., 2015). The study further found that inter-centromeric interactions decrease during quiescence, while inter-telomeric interactions increase in quiescence, indicating that quiescence maintenance is associated with substantial topological reorganization (Rutledge et al., 2015). Another study has also shown that when yeast enter the stationary phase, the telomeres hypercluster, that is, they colocalize in a single location (Laporte et al., 2016). Mutant yeast strains that lack linker histone Hho1, or condensin, or contain mutations in histone H4 lysine 16 are unable to form these telomere hyperclusters (Laporte et al., 2016). A contraction of the nucleolus (Wang et al., 2016) has also been observed in quiescent yeast cells as well.

A study in human diploid fibroblasts investigated changes in chromatin compaction with quiescence (Criscione et al., 2016). Using Formaldehyde Assisted Isolation of Regulatory Elements (FAIRE) to investigate global DNAse I sensitivity and chromatin accessibility, quiescent cells were found to be more resistant to DNAse I treatment, indicating more compact chromatin (Criscione et al., 2016). This study used Hi-C to show genes switching between A and B compartments with quiescence (Criscione et al., 2016). The A-type compartment has a more open chromatin structure and is enriched for activating marks such as H3K36me3, H3K79me2, H3K27ac, and H3K4me1 (Lieberman-Aiden et al., 2009; Rao et al., 2014). The B-type compartment, on the other hand, is characterized by more densely packed chromatin and correlates with repressive marks such as H3K27me3, H3K9me3, and H4K20me3 (Lieberman-Aiden et al., 2009; Rao et al., 2014). Genes associated with cell proliferation were enriched in the group of genes switching from A to B compartments as cells entered quiescence (Criscione et al., 2016).

HP1β is a dimeric protein that binds to the H3K9me3 mark in constitutive heterochromatin and can bridge two H3K9me3-containing nucleosomes (Machida et al., 2018). Both HP1β and H3K9me3 localized to constitutive heterochromatin regions in proliferating B lymphocytes, while in quiescent B lymphocytes they did not (Baxter et al., 2004). These findings suggest that the overall structure of DNA may be altered in quiescent B lymphocytes in a way that affects the accessibility of histone writers and readers, especially in constitutive heterochromatin.

One possible mechanism through which the histone code could affect chromatin state during quiescence involves the histone methyltransferase Suv4-20h2 that generates H4K20me3. As mentioned before, H4K20me3 is involved in heterochromatin formation and induces chromatin compaction (Evertts et al., 2013a; Hahn et al., 2013). Suv4-20h2 activity and the H4K20me3 mark are upregulated with quiescence (Evertts et al., 2013a; Bierhoff et al., 2014). A fluorescence *in situ* hybridization (FISH) analysis performed in primary human dermal fibroblasts found that contact-inhibited quiescent fibroblasts had more compact chromatin than their proliferating counterparts (Evertts et al., 2013a). Furthermore, knockdown of Suv20h2 resulted in decreased compaction (Evertts et al., 2013a). As described above, crystal structures of nucleosomes reconstituted with histones containing the H4K20me3 mark had alterations in higher order structure (Lu et al., 2008). Fluorescence Recovery After Photobleaching (FRAP) analysis revealed that Suv4-20h2 binds tightly to heterochromatin (Hahn et al., 2013). Other studies have shown that Suv4-20h2 associates with pericentric heterochromatin through multiple, independent interaction sites on its C-terminal domain that directly bind to multiple heterochromatin protein 1 (HP1) molecules (Schotta et al., 2004, 2008; Watanabe et al., 2018). The HP1 protein family consists of three members: HP1α, HP1β, and HP1γ. HP1α localizes to heterochromatin, HP1β is found in both heterochromatic and euchromatic regions and HP1γ is associated with actively transcribed genes (Vakoc et al., 2005; Lomberk et al., 2006). Dimeric HP1α binds two H3K9me3 marks in adjacent nucleosomes and forms a bridge between them, thereby compacting the chromatin (Watanabe et al., 2018). Consequently, the combinatorial effects of the H3K9me3 and H4K20me3 marks together represent an opportunity for a quiescence combinatorial histone code that affects chromatin conformation and induces chromosomal compaction.

Chromatin-bound Suv4-20h2 also recruits the cohesin complex (Hahn et al., 2013), which is composed of rings of Smc1-Smc3 dimers connected by Scc1/Rad21 (Nasmyth, 2011). The cohesin complex can contribute to establishment of the chromatin loop domains at specific DNA regions, thereby inducing chromatin compaction (Maya-Miles et al., 2019). In *Suv4-20h1/Suv4-20h2* double-knockout cells, cohesin was absent from heterochromatic regions in G0 phase, which showed that Suv4-20h enzymes are required for loading or maintaining cohesin at heterochromatin (Hahn et al., 2013). Reintroducing Suv4-20h2 or only the non-enzymatic clamp domain of Suv4-20h2 rescued the loss of heterochromatin-associated cohesin suggesting the effects of Suv4-20h2 may be mediated independent of its effects on H4K20me3 (Hahn et al., 2013). This ability of Suv4-20h2 to recruit cohesin and compact chromatin may be critical for chromatin compaction with quiescence, as Suv4-20h2-deficient MEFs synchronized in G_0_, contained virtually no cohesin in regions of heterochromatin (Hahn et al., 2013). Additional experiments will be required to understand the functional consequences of a lack of cohesin in heterochromatin in Suv4-20h2-deficient quiescent cells.

## Cross-Talk Between Histone Marks and Metabolism

An important emerging theme in epigenetic regulation is the close association between histone marks and metabolism. In yeast, metabolic signals such as the presence or absence of glucose can determine whether cells proliferate or arrest (Laporte et al., 2011), and therefore, histone marks that signal the relative abundance of glucose can potentially transmit information about nutrient abundance in a locus-specific manner to affect gene regulation. The metabolite acetyl-CoA serves as the substrate for histone acetyltransferase enzymes that generate acetylated histones (Kaelin and McKnight, 2013) (Figure 3B). Acetyl CoA is formed in mitochondria when pyruvate generated by glycolysis is committed to the TCA cycle (Martinez-Reyes and Chandel, 2020). Citrate formed in the mitochondria can be exported and converted in the cytoplasm to acetyl CoA (Martinez-Reyes and Chandel, 2020). In proliferating yeast and mammalian cells, higher levels of glycolysis and increased export of acetyl CoA from the mitochondria have been observed in proliferating compared with quiescent cells (Frauwirth and Thompson, 2004; Vander Heiden et al., 2009; Lemons et al., 2010). Thus, the metabolic profiles of proliferating cells may facilitate the generation of histone acetylation, and the formation of more open chromatin (Frauwirth and Thompson, 2004; Lemons et al., 2010). The role of histone acetylation in *S. pombe* was investigated with a strain that exhibited a temperature-sensitive mutation in the catalytic region of phosphopantothenoylcysteine synthetase (designated Ppc1) (Nakamura et al., 2012), an enzyme in the biosynthetic pathway for acetyl-CoA. *S. pombe* with mutations that inactivate the ability to synthesize acetyl-CoA fail to acetylate histones and are unable to re-enter the cell cycle after initiating G_0_ following nitrogen withdrawal (Nakamura et al., 2012).

In fibroblasts, the oncogene c-MYC has been identified as a transcription factor that can affect global chromatin remodeling (Morrish et al., 2010). In rat fibroblasts, MYC activity increased glucose metabolism and acetyl-CoA production (Morrish et al., 2010). The presence of MYC caused a forty percent increase in ^13^C-labeled acetyl-CoA on H4-K16 during cell cycle entry with serum stimulation during the G_0_ to S transition (Morrish et al., 2010). Metabolic tracing revealed that in MYC-stimulated cells, MYC increases acetylCoA levels (Morrish et al., 2010). Further, the GCN5 histone acetylase enzyme, an enzyme that is important for histone acetylation in response to nutrients in yeast (McMahon et al., 2000; Cai et al., 2011), is a target of MYC (Morrish et al., 2010).

In addition to a connection between metabolism and histone acetylation, there is also a connection between metabolism and histone methylation. S-adenosylmethionine (SAM) is a metabolite that acts as a donor of methyl groups, which are transferred by methyltransferase enzymes to histones (Kaelin and McKnight, 2013) (Figure 3C). In fission yeast, the enzyme responsible for synthesizing SAM, S-adenosylmethionine synthase 1 (Sam1), is required for proper cell growth, proliferation, and quiescence entry and exit (Hayashi et al., 2018). Loss of Sam1 results in reduced levels of H3K4me2 and H3K4me3, and a significant decrease in cell growth and defects in G2 cell cycle arrest (Hayashi et al., 2018). Yeast with mutations in Sam1 cannot survive in quiescence initiated following nitrogen starvation, and once released from G_0_, they are unable to increase in cell size and restart DNA replication in proliferative conditions (Hayashi et al., 2018).

Taken together, these studies exploring the crosstalk between metabolism and histone marks highlight the close association between nutrient uptake, proliferation and histone post-translational modifications. How this crosstalk is established and maintained in quiescence is an important area for further exploration.

## Gaps in the Field and Future Studies

The decision whether to proliferate or exit the proliferative cell cycle requires cells to integrate multiple types of information and execute a complex series of molecular changes that impact many of the cell’s activities. Transitioning between a proliferative and quiescent state is associated with many alterations including changes in the expression and activity of specific genes, changes in the conformation of chromatin, and changes in the subnuclear localization of chromosomes. The molecular mechanisms that cells use to make these decisions are actively being investigated and there are likely levels of regulation that have yet to be uncovered. Here we explored the literature to find evidence that histone post-translational modifications serve as a code that interprets information about the activity of signaling pathways and transmits that information to enable the commitment to a proliferative or quiescent state and the functional changes required for this transition.

So, is there a histone code for cellular quiescence? As highlighted in this review, there are multiple lines of evidence that suggest that specific histone modifications alone and in combination are added or removed as cells transition between proliferation and quiescent in multiple model systems. Histone H3K4me3, for instance, is found at the promoters of genes that are activated when cells become quiescent. H3K36 methylation is important for establishment and maintenance of the quiescent state in HSCs as inactivation of the SETD2 methyltransferase results in depletion of quiescent HSCs (Wagner and Carpenter, 2012). In quiescent mouse MuSCs, a combination of H3K9 and H3K27 marks was found to regulate cyclin A2 expression and proliferation (Cheedipudi et al., 2015). As another example, H4K20me3 levels increase in quiescent cells and knockdown of Suv4-20h2 results in a more rapid cell cycle (Evertts et al., 2013a). Finally, changes in the levels of histone acetylation have been observed with the transition between proliferation and quiescence (Ryall et al., 2015), and the close association between nutrient availability and acetyl CoA levels suggests histone acetylation as a possible link between nutrient availability and proliferation (Cai et al., 2011). For each of the marks described, there is evidence that the mark plays a functional role in some aspect of quiescence: quiescence entry, quiescence exit, quiescence maintenance, or quiescence depth. In some cases, functional studies have shown a causative role for a specific reader, writer or eraser. However, in many studies, and for many of the histone PTMs described, it is not clear whether the role of the histone marks is correlative or causative. Also, the contribution of histone marks seems to be context- and cell type-dependent as indicated by our survey of different quiescence models and conditions. For many of the marks, the direction of change was not consistent in different model systems. For instance, H3K27me3 was induced in some quiescence models and repressed in others (Baxter et al., 2004; Evertts et al., 2013a; Kallingappa et al., 2016; Maki et al., 2021). Other histone marks appear to be regulated with quiescence in some species, but are absent in others, such as H3K27me3 which is absent from yeast (Jamieson et al., 2013). Furthermore, quiescence studies have usually focused on one or a few histone marks at a time. Additional studies will be needed to determine whether a well-defined system-dependent or system-independent combinatorial histone code exists for cellular quiescence and how this histone code varies among species and tissues.

The findings in this review show that certain histone marks, and even combinations of histone marks, are altered with quiescence and some of these changes are consistent among model systems. Future studies will be needed that systematically determine the genomewide deposition of histone marks and combinations of histone marks in proliferating and quiescent cells in multiple model systems. These investigations would cover both traditional and non-traditional histone marks in multiple quiescent states. Beyond just comparing proliferating and quiescent cells, these inquiries would look into histone marks in cells that initiate quiescence in response to different signals in the same cell type and cells that have been quiescent for different durations of time to achieve different depths of quiescence (Rodgers et al., 2014; Kwon et al., 2017). While many of the studies we review have not discovered a clear relationship between changes in individual histone marks and altered gene expression (Liu et al., 2013; Lee et al., 2016), a careful analysis of combinations of marks might determine if the changes in the combinatorial pattern of histone marks is a better indicator of gene expression changes with quiescence.

Many questions remain when studying histone marks in quiescence. What triggers the deposition of these marks? In particular, how do different histone writers and readers coordinate to establish marks at the appropriate time? In some cases, establishment and maintenance of histone marks associated with an open chromatin structure and active gene transcription may be achieved through positive feedback loops (Zhang et al., 2015). These positive feedback loops can be formed when proteins that write histone marks also read the same mark (Zhang et al., 2015). For instance, SETD1 complexes not only catalyze the formation of H3K4me3 marks, but may also recognize the same H3K4me3 mark, bind to it, and continue to generate additional H3K4me3 modifications (Shi et al., 2007; Murton et al., 2010; Zhang et al., 2015). Dot1, the H3K79 methyltransferase in yeast (Guan et al., 2013), recognizes modifications on the histone H2B tail and, in human, also binds phosphorylated forms of RNA polymerase II at the transcription start sites of actively transcribed genes (Kim S.K. et al., 2012). H3K4me3 can recruit histone acetyltransferases that add acetyl groups as well as deacetylases that remove acetyl groups (Zhang et al., 2015), resulting in dynamic turnover of histone acetylation marks when H34me3, but not other marks such as H3K79me3 or H3K36me3, are present (Crump et al., 2011). For repressive chromatin, PRC2 not only generates H3K27me3 but also binds to it, resulting in a positive feedback loop in which local chromatin structure allows H3K27me3 to be deposited over chromatin regions to form domains (Hansen et al., 2008; Zhang et al., 2015). Crosstalk between H3K27me3 and monoubiquitinated H2A on lysine 119, H2AK119ul, has also been proposed as enzyme complexes that deposit each mark may recognize the other mark (Blackledge et al., 2014; Cooper et al., 2014; Kalb et al., 2014), allowing for the reinforcement of heterochromatic regions (Zhang et al., 2015). Negative feedback between histone marks also occurs as activating marks can inhibit the activity of enzymes that place repressive marks, and vice versa (Zhang et al., 2015). For instance, activating marks H3K4me2/3 and H3K36me2/3 can inhibit the activity of PRC2 and prevent the deposition of H3K27me3 repressive marks (Schmitges et al., 2011). Experiments in which the impact of modulating a specific reader and writer on the levels of multiple marks in the context of quiescence models may shed light on this question. In particular, such experiments may shed light on how positive and negative feedback loops are interrupted at proliferation-associated genes and reestablished at quiescence-associated genes.

The development and application of new technologies will facilitate future studies investigating how combinations of histone marks coordinate. One valuable approach will be the ability to visualize proliferation and quiescence decisions in organisms in real time. Toward this end, a recent paper describes the adaptation of a biosensor for CDK activity (Spencer et al., 2013) to monitor cell division in two model organisms, *Caenorhabditis elegans* and zebrafish (Adikes et al., 2020). CDK activity was higher at the end of a cell division in cases in which the cell went on to divide (Adikes et al., 2020). Such biosensors could be used in conjunction with visualization of histone marks to assess whether histone marks individually or in combination can predict whether a cell will proliferate.

We anticipate that CRISPR-Cas9 will prove to be a powerful methodology for understanding the impact of histone modifications. One important challenge in understanding the impact of different histone marks and combinations of histone marks has been developing specific systems to test their functional importance. Many studies to date have focused on investigating the role of specific readers and writers with knockdown and knockout approaches. Using CRISPR-Cas9, further studies will likely allow the inactivation of specific histone modifiers using protein degradation systems that allow the proteins to be degraded in proliferating or quiescent cells with defined timing thus permitting a more detailed dissection of their role (Wu et al., 2020).

Functional dissection of histone readers and writers is sometimes complicated as they have non-histone targets as well (Cornett et al., 2019). In fact, nearly 3,000 human non-histone proteins have been reported to have a lysine that can be methylated (Hornbeck et al., 2015; Cornett et al., 2019). An alternative approach is to test for the functional consequences of modifying the histones themselves. To achieve this, CRISPR-Cas9 has been used in *S. cerevisiae* to generate yeast strains with different combinations of mutations at histone tail lysines for histone H3 and H4, allowing the investigators to assess the effects of loss of different combinations of histone marks (Fu et al., 2021). In *Trypanosoma brucei*, precise editing of genes in multicopy arrays was performed with CRISPR-Cas9, allowing for the replacement of histone H4K4 with H4R4 to mimic the constitutively non-acetylated state. The authors achieved 90% replacement of the 43 histone H4 copies to H4R4 (Vasquez et al., 2018).

CRISPR/Cas9 will also be valuable for its capacity to specifically target chromatin writers and readers to specific genomic regions. As an early example of this technology, “programmable chromatin kinase” dCas9-dMSK1 was generated by fusing nuclease-deficient CRISPR/Cas9 (or dCas9) to a histone H3 kinase (Li et al., 2021). When this protein was targeted to specific promoters with guide RNAs, there was an increase in histone H3 serine 28 phosphorylation at the target genes’ promoters and an increase in expression of the targeted gene. Such studies are likely to be valuable for defining causal connections between histone PTMs, their activity at specific genomic loci, and outcomes such as gene expression and proliferation.

We anticipate that the availability of new technologies that allow us to better visualize the relationships among histone marks and chromosomal organization on a cell-by-cell basis will also benefit studies of the histone code in quiescence. A recent report built upon sequential FISH (seqFISH) and multiplexed FISH methods to target 3,660 loci in individual mouse ES cells (Takei et al., 2021). These studies revealed that nuclear zones were created by combinatorial chromatin patterns (Takei et al., 2021). Repressive histone marks H4K20me3, H3K9me3, and histone variant H2A1 were found together and colocalized with DAPI-rich regions (Takei et al., 2021). A second heterochromatic pattern of H4K20me2, H3K27me3 and H3K27me2 was also observed (Takei et al., 2021). Active histone marks, H3K9ac and H3K27ac, and RNA Polymerase II serine 5 phosphorylation localized to nuclear speckles and were excluded from heterochromatin and nuclear lamina (Takei et al., 2021). Similar studies comparing proliferating and quiescent cells could shed light on the role for a potential histone code in establishing a cell’s proliferative fate. In particular, single cell analyses of proliferating and quiescent cells could reveal patterns that are consistent among cells versus those that are more variable from cell to cell.

We anticipate that with time the role of additional histone PTMs and histone variants and their roles in regulating chromatin structure and gene expression will become clearer. As one example, non-tail globular histone marks, such as H3K36 and H3K122 acetylation marks found in active gene promoters and a subset of enhancers, can contribute to the histone code and expand the possibilities for combinatorial histone PTMs that provide position-specific information to readers (Pradeepa et al., 2016). Non-enzymatic histone modifications, such as glycation, acylation and lipidation, generated by spontaneous chemical reactions also have the potential to alter chromatin structure and regulate genetic processes (Maksimovic and David, 2021). The non-enzymatic modifications, like the histone marks described in this review, can be “erased” by scavenger systems (Maksimovic and David, 2021). Improved mass spectrometry and chemoproteomics will likely provide important new insights into the role of these modifications in cellular decisions including the commitment to proliferation (Maksimovic and David, 2021). As another example, additional studies are likely to reveal that changes in the specific histone variants present at different positions in the chromatin cooperate with histone PTMs to alter chromatin state and reader proteins. Some histone variants such as histone H3.1 and histone H3.3 contain different residues, such as amino acid S31 in histone H3.3 and A31 in histone H3.1 that can alter the properties of the chromatin and its accessibility to the transcription apparatus (Armache et al., 2020). Linker histone H1 variants can also affect folding of nucleosome arrays and nucleosome compaction (Fyodorov et al., 2018).

Combinations of multiple distinct genome-wide, high-throughput analyses performed in models of proliferating and quiescent cells, will also be needed to allow us to dissect the role of histone modifications and other contributors to gene expression and functional changes with quiescence. Studies in which changes in the genome-wide localization of histone marks can be correlated with chromatin accessibility using ATAC-seq, DNA methylation, and Hi-C to assess A and B compartments and topologically associating domain boundaries have the potential to yield greater insight. Combining these datasets and analyzing them with deep learning algorithms, may allow scientists to predict which combinations of marks are associated with changes in chromatin accessibility and gene expression.

## Conclusion

Based on the findings thus far, the specific histone marks discussed in this review, methylation of H3K4, H3K9, H3K27, H3K36, H3K79, H4K20, and acetylation of H3 and H4 have been discovered to be regulated with quiescence in different model systems. Readers, writers and erasers of these marks have been found to functionally contribute to the quiescence-proliferation transition and quiescence maintenance. The model systems employed for these studies include nutrient depletion and spore formation in yeast, cell culture models in which different anti-proliferative signals are employed, and models in which quiescent stem cells are visualized *in situ* or isolated and characterized. One of the key limitations for the field is the fact that knockout or knockdown of readers, writers and erasers can impact not just the histone marks under study, but also the PTMs of other cellular proteins as well. Also, most studies have investigated the effect of a single modification in isolation rather than the impact of several modifications together and the relationships between marks. More research with emerging technologies will be needed to determine whether there is a quiescence histone code and if so, how changes in histones are used to create a complex and context-dependent grammar that incorporates not just levels of histone marks and their readers, but also the chromatin context (in 2D as well as in 3D). Future studies will likely address the causality of histone marks and phenotypic changes that serve as regulators of quiescence and landmarks of quiescence. These studies may define the functional consequences of these marks in terms of gene expression, chromatin conformation, and chromosomal positioning. These studies will also likely reveal the role of traditional and non-traditional histone PTMs and other changes to chromatin in the decision whether to proliferate, the mechanisms that cause cell cycle arrest, the maintenance of cells during quiescence, and the determination of quiescence depth. Altogether, these results indicate that the regulation of histone marks may help to maintain the delicate balance between quiescence, proliferation, differentiation, and cell death, with different model systems and cell types likely using both overlapping and distinct aspects of the information contained in these histone marks.

## Author Contributions

MM conceived the topic and design of the review. KB and KS prepared all the figures and tables. KB, KS, MM, and HC did the literature survey. MM, HC, KB, KS, and KA wrote the manuscript. MM and HC supervised the project. All authors were involved in proof-reading.

## Conflict of Interest

The authors declare that the research was conducted in the absence of any commercial or financial relationships that could be construed as a potential conflict of interest.

## Publisher’s Note

All claims expressed in this article are solely those of the authors and do not necessarily represent those of their affiliated organizations, or those of the publisher, the editors and the reviewers. Any product that may be evaluated in this article, or claim that may be made by its manufacturer, is not guaranteed or endorsed by the publisher.

## References

[B1] Abbas T. Shibata E. Park J. Jha S. Karnani N. Dutta A. (2010). CRL4(Cdt2) regulates cell proliferation and histone gene expression by targeting PR-Set7/Set8 for degradation. *Mol. Cell* 40 9–21. 20932471 10.1016/j.molcel.2010.09.014 PMC2966975

[B2] Acquaviva L. Drogat J. Dehe P. M. de La Roche Saint-Andre C. Geli V. (2013). Spp1 at the crossroads of H3K4me3 regulation and meiotic recombination. *Epigenetics* 8 355–360. 10.4161/epi.24295 23511748 PMC3674044

[B3] Adikes R. C. Kohrman A. Q. Martinez M. A. Q. Palmisano N. J. Smith J. J. Medwig-Kinney T. N. (2020). Visualizing the metazoan proliferation-quiescence decision in vivo. *Elife* 9:e63265. 10.7554/eLife.63265 33350383 PMC7880687

[B4] Agalioti T. Chen G. Thanos D. (2002). Deciphering the transcriptional histone acetylation code for a human gene. *Cell* 111 381–392. 12419248 10.1016/s0092-8674(02)01077-2

[B5] Allen C. Büttner S. Aragon A. D. Thomas J. A. Meirelles O. Jaetao J. E. (2006). Isolation of quiescent and nonquiescent cells from yeast stationary-phase cultures. *J. Cell Biol.* 174 89–100. 16818721 10.1083/jcb.200604072 PMC2064167

[B6] Allfrey V. G. Faulkner R. Mirsky A. E. (1964). Acetylation and methylation of histones and their possible role in the regulation of Rna synthesis. *Proc. Natl. Acad. Sci. U.S.A.* 51 786–794. 14172992 10.1073/pnas.51.5.786 PMC300163

[B7] Allis C. D. Jenuwein T. (2016). The molecular hallmarks of epigenetic control. *Nat. Rev. Genet.* 17 487–500. 27346641 10.1038/nrg.2016.59

[B8] Armache A. Yang S. Martinez de Paz A. Robbins L. E. Durmaz C. Cheong J. Q. (2020). Histone H3.3 phosphorylation amplifies stimulation-induced transcription. *Nature* 583 852–857. 10.1038/s41586-020-2533-0 32699416 PMC7517595

[B9] Atlasi Y. Megchelenbrink W. Peng T. Habibi E. Joshi O. Wang S. Y. (2019). Epigenetic modulation of a hardwired 3D chromatin landscape in two naive states of pluripotency. *Nat. Cell Biol.* 21 568–578. 31036938 10.1038/s41556-019-0310-9

[B10] Bannister A. J. Kouzarides T. (2011). Regulation of chromatin by histone modifications. *Cell Res.* 21 381–395. 21321607 10.1038/cr.2011.22 PMC3193420

[B11] Bannister A. J. Zegerman P. Partridge J. F. Miska E. A. Thomas J. O. Allshire R. C. (2001). Selective recognition of methylated lysine 9 on histone H3 by the HP1 chromo domain. *Nature* 410 120–124. 11242054 10.1038/35065138

[B12] Barbieri M. Xie S. Q. Torlai Triglia E. Chiariello A. M. Bianco S. de Santiago I. (2017). Active and poised promoter states drive folding of the extended HoxB locus in mouse embryonic stem cells. *Nat. Struct. Mol. Biol.* 24 515–524. 10.1038/nsmb.3402 28436944

[B13] Barker N. van Es J. H. Kuipers J. Kujala P. van den Born M. Cozijnsen M. (2007). Identification of stem cells in small intestine and colon by marker gene Lgr5. *Nature* 449 1003–1007. 17934449 10.1038/nature06196

[B14] Barnes C. E. English D. M. Cowley S. M. (2019). Acetylation & Co: an expanding repertoire of histone acylations regulates chromatin and transcription. *Essays Biochem.* 63 97–107. 30940741 10.1042/EBC20180061 PMC6484784

[B15] Barski A. Cuddapah S. Cui K. Roh T. Y. Schones D. E. Wang Z. (2007). High-resolution profiling of histone methylations in the human genome. *Cell* 129 823–837. 17512414 10.1016/j.cell.2007.05.009

[B16] Bartolomei M. S. (2009). Genomic imprinting: employing and avoiding epigenetic processes. *Genes Dev.* 23 2124–2133. 10.1101/gad.1841409 19759261 PMC2751984

[B17] Basak O. Krieger T. G. Muraro M. J. Wiebrands K. Stange D. E. Frias-Aldeguer J. (2018). Troy+ brain stem cells cycle through quiescence and regulate their number by sensing niche occupancy. *Proc. Natl. Acad. Sci. U.S.A.* 115 E610–E619. 10.1073/pnas.1715911114 29311336 PMC5789932

[B18] Batista P. J. Chang H. Y. (2013). Long noncoding RNAs: cellular address codes in development and disease. *Cell* 152 1298–1307. 23498938 10.1016/j.cell.2013.02.012 PMC3651923

[B19] Baxter J. Sauer S. Peters A. John R. Williams R. Caparros M. L. (2004). Histone hypomethylation is an indicator of epigenetic plasticity in quiescent lymphocytes. *EMBO J.* 23 4462–4472. 10.1038/sj.emboj.7600414 15510223 PMC526455

[B20] Beck D. B. Oda H. Shen S. S. Reinberg D. (2012). PR-Set7 and H4K20me1: at the crossroads of genome integrity, cell cycle, chromosome condensation, and transcription. *Genes Dev.* 26 325–337. 10.1101/gad.177444.111 22345514 PMC3289880

[B21] Ben Hassine S. Arcangioli B. (2009). Tdp1 protects against oxidative DNA damage in non-dividing fission yeast. *EMBO J.* 28 632–640. 19197239 10.1038/emboj.2009.9 PMC2666031

[B22] Benetti R. Gonzalo S. Jaco I. Schotta G. Klatt P. Jenuwein T. (2007). Suv4-20h deficiency results in telomere elongation and derepression of telomere recombination. *J. Cell Biol.* 178 925–936. 10.1083/jcb.200703081 17846168 PMC2064618

[B23] Bernhart S. H. Kretzmer H. Holdt L. M. Juhling F. Ammerpohl O. Bergmann A. K. (2016). Changes of bivalent chromatin coincide with increased expression of developmental genes in cancer. *Sci. Rep.* 6:37393. 10.1038/srep37393 27876760 PMC5120258

[B24] Bernstein B. E. Mikkelsen T. S. Xie X. Kamal M. Huebert D. J. Cuff J. (2006). A bivalent chromatin structure marks key developmental genes in embryonic stem cells. *Cell* 125 315–326. 16630819 10.1016/j.cell.2006.02.041

[B25] Bhattacharya S. Levy M. J. Zhang N. Li H. Florens L. Washburn M. P. (2021). The methyltransferase SETD2 couples transcription and splicing by engaging mRNA processing factors through its SHI domain. *Nat. Commun.* 12:1443. 10.1038/s41467-021-21663-w 33664260 PMC7933334

[B26] Bierhoff H. Dammert M. A. Brocks D. Dambacher S. Schotta G. Grummt I. (2014). Quiescence-induced LncRNAs trigger H4K20 trimethylation and transcriptional silencing. *Mol. Cell* 54 675–682. 10.1016/j.molcel.2014.03.032 24768537

[B27] Bierhoff H. Schmitz K. Maass F. Ye J. Grummt I. (2010). Noncoding transcripts in sense and antisense orientation regulate the epigenetic state of ribosomal RNA genes. *Cold Spring Harb. Symp. Quant. Biol.* 75 357–364. 10.1101/sqb.2010.75.060 21502405

[B28] Bjornson C. R. Cheung T. H. Liu L. Tripathi P. V. Steeper K. M. Rando T. A. (2012). Notch signaling is necessary to maintain quiescence in adult muscle stem cells. *Stem Cells* 30 232–242. 22045613 10.1002/stem.773 PMC3384696

[B29] Black J. C. Van Rechem C. Whetstine J. R. (2012). Histone lysine methylation dynamics: establishment, regulation, and biological impact. *Mol. Cell* 48 491–507. 23200123 10.1016/j.molcel.2012.11.006 PMC3861058

[B30] Blackledge N. P. Farcas A. M. Kondo T. King H. W. McGouran J. F. Hanssen L. L. P. (2014). Variant PRC1 complex-dependent H2A ubiquitylation drives PRC2 recruitment and polycomb domain formation. *Cell* 157 1445–1459. 10.1016/j.cell.2014.05.004 24856970 PMC4048464

[B31] Boonsanay V. Zhang T. Georgieva A. Kostin S. Qi H. Yuan X. (2016). Regulation of skeletal muscle stem cell quiescence by Suv4-20h1-dependent facultative heterochromatin formation. *Cell Stem Cell* 18 229–242. 10.1016/j.stem.2015.11.002 26669898

[B32] Boroviak T. Loos R. Bertone P. Smith A. Nichols J. (2014). The ability of inner-cell-mass cells to self-renew as embryonic stem cells is acquired following epiblast specification. *Nat. Cell Biol.* 16 516–528. 10.1038/ncb2965 24859004 PMC4878656

[B33] Bousard A. Raposo A. C. Zylicz J. J. Picard C. Pires V. B. Qi Y. (2019). The role of Xist-mediated Polycomb recruitment in the initiation of X-chromosome inactivation. *EMBO Rep.* 20:e48019. 10.15252/embr.201948019 31456285 PMC6776897

[B34] Bridger J. M. Boyle S. Kill I. R. Bickmore W. A. (2000). Re-modelling of nuclear architecture in quiescent and senescent human fibroblasts. *Curr. Biol.* 10 149–152. 10.1016/s0960-9822(00)00312-2 10679329

[B35] Cai L. Sutter B. M. Li B. Tu B. P. (2011). Acetyl-CoA induces cell growth and proliferation by promoting the acetylation of histones at growth genes. *Mol. Cell* 42 426–437. 10.1016/j.molcel.2011.05.004 21596309 PMC3109073

[B36] Carvalho S. Raposo A. C. Martins F. B. Grosso A. R. Sridhara S. C. Rino J. (2013). Histone methyltransferase SETD2 coordinates FACT recruitment with nucleosome dynamics during transcription. *Nucleic Acids Res.* 41 2881–2893. 10.1093/nar/gks1472 23325844 PMC3597667

[B37] Centore R. C. Havens C. G. Manning A. L. Li J. M. Flynn R. L. Tse A. (2010). CRL4(Cdt2)-mediated destruction of the histone methyltransferase Set8 prevents premature chromatin compaction in S phase. *Mol. Cell* 40 22–33. 10.1016/j.molcel.2010.09.015 20932472 PMC2957874

[B38] Champagne K. S. Kutateladze T. G. (2009). Structural insight into histone recognition by the ING PHD fingers. *Curr. Drug Targets* 10 432–441. 10.2174/138945009788185040 19442115 PMC2740728

[B39] Chang H. Y. Chi J. T. Dudoit S. Bondre C. van de Rijn M. Botstein D. (2002). Diversity, topographic differentiation, and positional memory in human fibroblasts. *Proc. Natl. Acad. Sci. U.S.A.* 99 12877–12882. 12297622 10.1073/pnas.162488599 PMC130553

[B40] Cheedipudi S. Puri D. Saleh A. Gala H. P. Rumman M. Pillai M. S. (2015). A fine balance: epigenetic control of cellular quiescence by the tumor suppressor PRDM2/RIZ at a bivalent domain in the cyclin a gene. *Nucleic Acids Res.* 43 6236–6256. 10.1093/nar/gkv567 26040698 PMC4513853

[B41] Cheung T. H. Rando T. A. (2013). Molecular regulation of stem cell quiescence. *Nat. Rev. Mol. Cell Biol.* 14 329–340. 23698583 10.1038/nrm3591 PMC3808888

[B42] Cheung T. H. Quach N. L. Charville G. W. Liu L. Park L. Edalati A. (2012). Maintenance of muscle stem-cell quiescence by microRNA-489. *Nature* 482 524–528. 10.1038/nature10834 22358842 PMC3292200

[B43] Chi P. Allis C. D. Wang G. G. (2010). Covalent histone modifications–miswritten, misinterpreted and mis-erased in human cancers. *Nat. Rev. Cancer* 10 457–469. 10.1038/nrc2876 20574448 PMC3262678

[B44] Chiu N. Baserga R. (1975). Changes in template activity and structure of nuclei from WI-38 cells in the prereplicative phase. *Biochemistry* 14 3126–3132. 10.1021/bi00685a014 1170886

[B45] Chu D. Barnes D. J. (2016). The lag-phase during diauxic growth is a trade-off between fast adaptation and high growth rate. *Sci. Rep.* 6:25191. 10.1038/srep25191 27125900 PMC4850433

[B46] Coller H. A. (2019a). Regulation of cell cycle entry and exit: a single cell perspective. *Compr. Physiol.* 10 317–344. 10.1002/cphy.c190014 31853969 PMC8208229

[B47] Coller H. A. (2019b). The paradox of metabolism in quiescent stem cells. *FEBS Lett.* 593 2817–2839. 31531979 10.1002/1873-3468.13608 PMC7034665

[B48] Coller H. A. Sang L. Roberts J. M. (2006). A new description of cellular quiescence. *PLoS Biol.* 4:e83. 10.1371/journal.pbio.0040083 16509772 PMC1393757

[B49] Cooper S. Dienstbier M. Hassan R. Schermelleh L. Sharif J. Blackledge N. P. (2014). Targeting polycomb to pericentric heterochromatin in embryonic stem cells reveals a role for H2AK119u1 in PRC2 recruitment. *Cell Rep.* 7 1456–1470. 10.1016/j.celrep.2014.04.012 24857660 PMC4062935

[B50] Coppock D. L. Kopman C. Scandalis S. Gilleran S. (1993). Preferential gene expression in quiescent human lung fibroblasts. *Cell Growth Differ.* 4 483–493. 8396966

[B51] Cornett E. M. Ferry L. Defossez P. A. Rothbart S. B. (2019). Lysine methylation regulators moonlighting outside the epigenome. *Mol. Cell* 75 1092–1101. 10.1016/j.molcel.2019.08.026 31539507 PMC6756181

[B52] Cortini R. (2016). The physics of epigenetics. *Rev. Modern Phys.* 88:025002.

[B53] Corvalan A. Z. Coller H. A. (2021). Methylation of histone 4’s lysine 20: a critical analysis of the state of the field. *Physiol. Genomics* 53 22–32. 10.1152/physiolgenomics.00128.2020 33197229 PMC7847046

[B54] Cosgrove M. S. (2012). Writers and readers: deconvoluting the harmonic complexity of the histone code. *Nat. Struct. Mol. Biol.* 19 739–740. 10.1038/nsmb.2350 22864360

[B55] Cosgrove M. S. Boeke J. D. Wolberger C. (2004). Regulated nucleosome mobility and the histone code. *Nat. Struct. Mol. Biol.* 11 1037–1043. 10.1038/nsmb851 15523479

[B56] Criscione S. W. De Cecco M. Siranosian B. Zhang Y. Kreiling J. A. Sedivy J. M. (2016). Reorganization of chromosome architecture in replicative cellular senescence. *Sci. Adv.* 2:e1500882. 10.1126/sciadv.1500882 PMC4788486 26989773

[B57] Crump N. T. Hazzalin C. A. Bowers E. M. Alani R. M. Cole P. A. Mahadevan L. C. (2011). Dynamic acetylation of all lysine-4 trimethylated histone H3 is evolutionarily conserved and mediated by p300/CBP. *Proc. Natl. Acad. Sci. U.S.A.* 108 7814–7819. 10.1073/pnas.1100099108 21518915 PMC3093510

[B58] Cui K. Zang C. Roh T. Y. Schones D. E. Childs R. W. Peng W. (2009). Chromatin signatures in multipotent human hematopoietic stem cells indicate the fate of bivalent genes during differentiation. *Cell Stem Cell* 4 80–93. 10.1016/j.stem.2008.11.011 19128795 PMC2785912

[B59] Cutter A. R. Hayes J. J. (2015). A brief review of nucleosome structure. *FEBS Lett.* 589 2914–2922. 25980611 10.1016/j.febslet.2015.05.016 PMC4598263

[B60] Dai J. Itahana K. Baskar R. (2015). Quiescence does not affect p53 and stress response by irradiation in human lung fibroblasts. *Biochem. Biophys. Res. Commun.* 458 104–109. 25637534 10.1016/j.bbrc.2015.01.076

[B61] Daniel J. A. Nussenzweig A. (2012). Roles for histone H3K4 methyltransferase activities during immunoglobulin class-switch recombination. *Biochim. Biophys. Acta* 1819 733–738. 22710321 10.1016/j.bbagrm.2012.01.019 PMC3378979

[B62] Dardick I. Sinnott N. M. Hall R. Bajenko-Carr T. A. Setterfield G. (1983). Nuclear morphology and morphometry of B-lymphocyte transformation. Implications for follicular center cell lymphomas. *Am. J. Pathol.* 111 35–49. 6340518 PMC1916206

[B63] De Virgilio C. (2012). The essence of yeast quiescence. *FEMS Microbiol. Rev.* 36 306–339. 21658086 10.1111/j.1574-6976.2011.00287.x

[B64] DeChiara T. M. Robertson E. J. Efstratiadis A. (1991). Parental imprinting of the mouse insulin-like growth factor II gene. *Cell* 64 849–859. 1997210 10.1016/0092-8674(91)90513-x

[B65] Deng Z. Norseen J. Wiedmer A. Riethman H. Lieberman P. M. (2009). TERRA RNA binding to TRF2 facilitates heterochromatin formation and ORC recruitment at telomeres. *Mol. Cell* 35 403–413. 10.1016/j.molcel.2009.06.025 19716786 PMC2749977

[B66] DesJarlais R. Tummino P. J. (2016). Role of histone-modifying enzymes and their complexes in regulation of chromatin biology. *Biochemistry* 55 1584–1599. 26745824 10.1021/acs.biochem.5b01210

[B67] Dhawan J. Laxman S. (2015). Decoding the stem cell quiescence cycle–lessons from yeast for regenerative biology. *J. Cell Sci.* 128 4467–4474. 10.1242/jcs.177758 26672015 PMC5695657

[B68] Dixon J. R. Selvaraj S. Yue F. Kim A. Li Y. Shen Y. (2012). Topological domains in mammalian genomes identified by analysis of chromatin interactions. *Nature* 485 376–380. 22495300 10.1038/nature11082 PMC3356448

[B69] Doil C. Mailand N. Bekker-Jensen S. Menard P. Larsen D. H. Pepperkok R. (2009). RNF168 binds and amplifies ubiquitin conjugates on damaged chromosomes to allow accumulation of repair proteins. *Cell* 136 435–446. 10.1016/j.cell.2008.12.041 19203579

[B70] Duina A. A. Miller M. E. Keeney J. B. (2014). Budding yeast for budding geneticists: a primer on the Saccharomyces cerevisiae model system. *Genetics* 197 33–48. 24807111 10.1534/genetics.114.163188 PMC4012490

[B71] Dumont N. A. Wang Y. X. Rudnicki M. A. (2015). Intrinsic and extrinsic mechanisms regulating satellite cell function. *Development* 142 1572–1581. 25922523 10.1242/dev.114223 PMC4419274

[B72] Dykstra B. Kent D. Bowie M. McCaffrey L. Hamilton M. Lyons K. (2007). Long-term propagation of distinct hematopoietic differentiation programs in vivo. *Cell Stem Cell* 1 218–229. 10.1016/j.stem.2007.05.015 18371352

[B73] Edwards C. R. Dang W. Berger S. L. (2011). Histone H4 lysine 20 of Saccharomyces cerevisiae is monomethylated and functions in subtelomeric silencing. *Biochemistry* 50 10473–10483. 10.1021/bi201120q 21985125 PMC3697087

[B74] Egelhofer T. A. Minoda A. Klugman S. Lee K. Kolasinska-Zwierz P. Alekseyenko A. A. (2011). An assessment of histone-modification antibody quality. *Nat. Struct. Mol. Biol.* 18 91–93. 21131980 10.1038/nsmb.1972 PMC3017233

[B75] Eriksson P. R. Ganguli D. Nagarajavel V. Clark D. J. (2012). Regulation of histone gene expression in budding yeast. *Genetics* 191 7–20. 22555441 10.1534/genetics.112.140145 PMC3338271

[B76] Ernst J. Kellis M. (2010). Discovery and characterization of chromatin states for systematic annotation of the human genome. *Nat. Biotechnol.* 28 817–825. 20657582 10.1038/nbt.1662 PMC2919626

[B77] Evertts A. G. Manning A. L. Wang X. Dyson N. J. Garcia B. A. Coller H. A. (2013a). H4K20 methylation regulates quiescence and chromatin compaction. *Mol. Biol. Cell* 24 3025–3037. 23924899 10.1091/mbc.E12-07-0529 PMC3784377

[B78] Evertts A. G. Zee B. M. Dimaggio P. A. Gonzales-Cope M. Coller H. A. Garcia B. A. (2013b). Quantitative dynamics of the link between cellular metabolism and histone acetylation. *J. Biol. Chem.* 288 12142–12151. 23482559 10.1074/jbc.M112.428318 PMC3636898

[B79] Faghihi M. A. Wahlestedt C. (2009). Regulatory roles of natural antisense transcripts. *Nat. Rev. Mol. Cell Biol.* 10 637–643. 19638999 10.1038/nrm2738 PMC2850559

[B80] Fang J. Feng Q. Ketel C. S. Wang H. Cao R. Xia L. (2002). Purification and functional characterization of SET8, a nucleosomal histone H4-lysine 20-specific methyltransferase. *Curr. Biol.* 12 1086–1099. 10.1016/s0960-9822(02)00924-7 12121615

[B81] Farooq Z. Banday S. Pandita T. K. Altaf M. (2016). The many faces of histone H3K79 methylation. *Mutat. Res. Rev. Mutat. Res.* 768 46–52. 10.1016/j.mrrev.2016.03.005 27234562 PMC4889126

[B82] Farrelly L. A. Maze I. (2019). An emerging perspective on ‘histone code’ mediated regulation of neural plasticity and disease. *Curr. Opin. Neurobiol.* 59 157–163. 10.1016/j.conb.2019.07.001 31382083 PMC6889037

[B83] Farrelly L. A. Thompson R. E. Zhao S. Lepack A. E. Lyu Y. Bhanu N. V. (2019). Histone serotonylation is a permissive modification that enhances TFIID binding to H3K4me3. *Nature* 567 535–539. 10.1038/s41586-019-1024-7 30867594 PMC6557285

[B84] Finley L. W. S. Vardhana S. A. Carey B. W. Alonso-Curbelo D. Koche R. Chen Y. (2018). Pluripotency transcription factors and Tet1/2 maintain Brd4-independent stem cell identity. *Nat. Cell Biol.* 20 565–574. 10.1038/s41556-018-0086-3 29662175 PMC5937285

[B85] Foudi A. Hochedlinger K. Van Buren D. Schindler J. W. Jaenisch R. Carey V. (2009). Analysis of histone 2B-GFP retention reveals slowly cycling hematopoietic stem cells. *Nat. Biotechnol.* 27 84–90. 10.1038/nbt.1517 19060879 PMC2805441

[B86] Frauwirth K. A. Thompson C. B. (2004). Regulation of T lymphocyte metabolism. *J. Immunol.* 172 4661–4665. 15067038 10.4049/jimmunol.172.8.4661

[B87] Frederiks F. Tzouros M. Oudgenoeg G. van Welsem T. Fornerod M. Krijgsveld J. (2008). Nonprocessive methylation by Dot1 leads to functional redundancy of histone H3K79 methylation states. *Nat. Struct. Mol. Biol.* 15 550–557. 10.1038/nsmb.1432 18511943

[B88] Freese E. B. Chu M. I. Freese E. (1982). Initiation of yeast sporulation of partial carbon, nitrogen, or phosphate deprivation. *J. Bacteriol.* 149 840–851. 7037742 10.1128/jb.149.3.840-851.1982 PMC216470

[B89] Fu Y. Zhu Z. Meng G. Zhang R. Zhang Y. (2021). A CRISPR-Cas9 based shuffle system for endogenous histone H3 and H4 combinatorial mutagenesis. *Sci. Rep.* 11:3298. 10.1038/s41598-021-82774-4 33558622 PMC7870972

[B90] Fuchs S. M. Krajewski K. Baker R. W. Miller V. L. Strahl B. D. (2011). Influence of combinatorial histone modifications on antibody and effector protein recognition. *Curr. Biol.* 21 53–58. 10.1016/j.cub.2010.11.058 21167713 PMC3019281

[B91] Fukada S. Uezumi A. Ikemoto M. Masuda S. Segawa M. Tanimura N. (2007). Molecular signature of quiescent satellite cells in adult skeletal muscle. *Stem Cells* 25 2448–2459. 17600112 10.1634/stemcells.2007-0019

[B92] Fyodorov D. V. Zhou B. R. Skoultchi A. I. Bai Y. (2018). Emerging roles of linker histones in regulating chromatin structure and function. *Nat. Rev. Mol. Cell Biol.* 19 192–206. 29018282 10.1038/nrm.2017.94 PMC5897046

[B93] Gaertner B. Johnston J. Chen K. Wallaschek N. Paulson A. Garruss A. S. (2012). Poised RNA polymerase II changes over developmental time and prepares genes for future expression. *Cell Rep.* 2 1670–1683. 10.1016/j.celrep.2012.11.024 23260668 PMC3572839

[B94] Galdieri L. Mehrotra S. Yu S. Vancura A. (2010). Transcriptional regulation in yeast during diauxic shift and stationary phase. *OMICS* 14 629–638. 20863251 10.1089/omi.2010.0069 PMC3133784

[B95] Gangloff S. Achaz G. Francesconi S. Villain A. Miled S. Denis C. (2017). Quiescence unveils a novel mutational force in fission yeast. *Elife* 6:e27469. 10.7554/eLife.27469 29252184 PMC5734874

[B96] Gangloff S. Arcangioli B. (2017). DNA repair and mutations during quiescence in yeast. *FEMS Yeast Res.* 17:fox002. 10.1093/femsyr/fox002 28087675

[B97] Garza L. A. Yang C. C. Zhao T. Blatt H. B. Lee M. He H. (2011). Bald scalp in men with androgenetic alopecia retains hair follicle stem cells but lacks CD200-rich and CD34-positive hair follicle progenitor cells. *J. Clin. Invest.* 121 613–622. 10.1172/JCI44478 21206086 PMC3026732

[B98] Ghoneim M. Fuchs H. A. Musselman C. A. (2021). Histone tail conformations: a fuzzy affair with DNA. *Trends Biochem. Sci.* 46 564–578. 10.1016/j.tibs.2020.12.012 33551235 PMC8195839

[B99] Godfrey L. Crump N. T. Thorne R. Lau I. J. Repapi E. Dimou D. (2019). DOT1L inhibition reveals a distinct subset of enhancers dependent on H3K79 methylation. *Nat. Commun.* 10:2803. 10.1038/s41467-019-10844-3 31243293 PMC6594956

[B100] Godley L. A. Le Beau M. M. (2012). The histone code and treatments for acute myeloid leukemia. *N. Engl. J. Med.* 366 960–961. 22397660 10.1056/NEJMcibr1113401

[B101] Gray J. V. Petsko G. A. Johnston G. C. Ringe D. Singer R. A. Werner-Washburne M. (2004). “Sleeping beauty”: quiescence in *Saccharomyces* cerevisiae. *Microbiol. Mol. Biol. Rev.* 68 187–206. 15187181 10.1128/MMBR.68.2.187-206.2004 PMC419917

[B102] Greer E. L. Shi Y. (2012). Histone methylation: a dynamic mark in health, disease and inheritance. *Nat. Rev. Genet.* 13 343–357. 10.1038/nrg3173 22473383 PMC4073795

[B103] Greig D. (2009). Reproductive isolation in *Saccharomyces*. *Heredity (Edinb)* 102 39–44. 18648383 10.1038/hdy.2008.73

[B104] Grigoryev S. A. Nikitina T. Pehrson J. R. Singh P. B. Woodcock C. L. (2004). Dynamic relocation of epigenetic chromatin markers reveals an active role of constitutive heterochromatin in the transition from proliferation to quiescence. *J. Cell Sci.* 117(Pt 25) 6153–6162. 10.1242/cs.01537 15564378

[B105] Guan X. Rastogi N. Parthun M. R. Freitas M. A. (2013). Discovery of histone modification crosstalk networks by stable isotope labeling of amino acids in cell culture mass spectrometry (SILAC MS). *Mol. Cell. Proteomics* 12 2048–2059. 10.1074/mcp.M112.026716 23592332 PMC3734568

[B106] Guidi M. Ruault M. Marbouty M. Loiodice I. Cournac A. Billaudeau C. (2015). Spatial reorganization of telomeres in long-lived quiescent cells. *Genome Biol.* 16:206. 10.1186/s13059-015-0766-2 26399229 PMC4581094

[B107] Guttman M. Donaghey J. Carey B. W. Garber M. Grenier J. K. Munson G. (2011). lincRNAs act in the circuitry controlling pluripotency and differentiation. *Nature* 477 295–300. 21874018 10.1038/nature10398 PMC3175327

[B108] Hahn M. Dambacher S. Dulev S. Kuznetsova A. Y. Eck S. Wörz S. (2013). Suv4-20h2 mediates chromatin compaction and is important for cohesin recruitment to heterochromatin. *Genes Dev.* 27 859–872. 10.1101/gad.210377.112 23599346 PMC3650224

[B109] Hainer S. J. Fazzio T. G. (2019). High-Resolution chromatin profiling using CUT&RUN. *Curr. Protoc. Mol. Biol.* 126:e85. 10.1002/cpmb.85 PMC6422702 30688406

[B110] Hanly D. J. Esteller M. Berdasco M. (2018). Interplay between long non-coding RNAs and epigenetic machinery: emerging targets in cancer? *Philos. Trans. R. Soc. Lond. B Biol. Sci.* 373:20170074. 10.1098/rstb.2017.0074 29685978 PMC5915718

[B111] Hansen K. H. Bracken A. P. Pasini D. Dietrich N. Gehani S. S. Monrad A. (2008). A model for transmission of the H3K27me3 epigenetic mark. *Nat. Cell Biol.* 10 1291–1300. 10.1038/ncb1787 18931660

[B112] Hayashi T. Teruya T. Chaleckis R. Morigasaki S. Yanagida M. (2018). S-Adenosylmethionine synthetase is required for cell growth, maintenance of G0 phase, and termination of quiescence in fission yeast. *iScience* 5 38–51. 10.1016/j.isci.2018.06.011 30240645 PMC6123894

[B113] Hayashi-Takanaka Y. Kina Y. Nakamura F. Becking L. E. Nakao Y. Nagase T. (2020). Histone modification dynamics as revealed by multicolor immunofluorescence-based single-cell analysis. *J. Cell Sci.* 133 10.1242/jcs.243444 32576661 PMC7390643

[B114] Henikoff S. Shilatifard A. (2011). Histone modification: cause or cog? *Trends Genet.* 27 389–396. 21764166 10.1016/j.tig.2011.06.006

[B115] Higuchi-Sanabria R. Pernice W. M. Vevea J. D. Alessi Wolken D. M. Boldogh I. R. Pon L. A. (2014). Role of asymmetric cell division in lifespan control in *Saccharomyces cerevisiae*. *FEMS Yeast Res.* 14 1133–1146. 25263578 10.1111/1567-1364.12216 PMC4270926

[B116] Ho C. H. Takizawa Y. Kobayashi W. Arimura Y. Kimura H. Kurumizaka H. (2021). Structural basis of nucleosomal histone H4 lysine 20 methylation by SET8 methyltransferase. *Life Sci. Alliance* 4:e202000919. 10.26508/lsa.202000919 PMC7893823 33574035

[B117] Ho J. W. Jung Y. L. Liu T. Alver B. H. Lee S. Ikegami K. (2014). Comparative analysis of metazoan chromatin organization. *Nature* 512 449–452. 25164756 10.1038/nature13415 PMC4227084

[B118] Hornbeck P. V. Zhang B. Murray B. Kornhauser J. M. Latham V. Skrzypek E. (2015). PhosphoSitePlus, 2014: mutations, PTMs and recalibrations. *Nucleic Acids Res.* 43 D512–D520. 10.1093/nar/gku1267 25514926 PMC4383998

[B119] Huang J. Gujar M. R. Deng Q. Sook Y. C. Li S. Tan P. (2021). Histone lysine methyltransferase Pr-set7/SETD8 promotes neural stem cell reactivation. *EMBO Rep.* 2021:e50994. 10.15252/embr.202050994 33565211 PMC8024890

[B120] Huen M. S. Grant R. Manke I. Minn K. Yu X. Yaffe M. B. (2007). RNF8 transduces the DNA-damage signal via histone ubiquitylation and checkpoint protein assembly. *Cell* 131 901–914. 10.1016/j.cell.2007.09.041 18001825 PMC2149842

[B121] Husmann D. Gozani O. (2019). Histone lysine methyltransferases in biology and disease. *Nat. Struct. Mol. Biol.* 26 880–889. 31582846 10.1038/s41594-019-0298-7 PMC6951022

[B122] Hyun K. Jeon J. Park K. Kim J. (2017). Writing, erasing and reading histone lysine methylations. *Exp. Mol. Med.* 49:e324. 10.1038/emm.2017.11 PMC6130214 28450737

[B123] Jambhekar A. Dhall A. Shi Y. (2019). Roles and regulation of histone methylation in animal development. *Nat. Rev. Mol. Cell Biol.* 20 625–641. 31267065 10.1038/s41580-019-0151-1 PMC6774358

[B124] Jamieson K. Rountree M. R. Lewis Z. A. Stajich J. E. Selker E. U. (2013). Regional control of histone H3 lysine 27 methylation in Neurospora. *Proc. Natl. Acad. Sci. U.S.A.* 110 6027–6032. 23530226 10.1073/pnas.1303750110 PMC3625340

[B125] Jenuwein T. Allis C. D. (2001). Translating the histone code. *Science* 293 1074–1080. 11498575 10.1126/science.1063127

[B126] Joh R. I. Khanduja J. S. Calvo I. A. Mistry M. Palmieri C. M. Savol A. J. (2016). Survival in quiescence requires the euchromatic deployment of Clr4/SUV39H by argonaute-associated small RNAs. *Mol. Cell* 64 1088–1101. 10.1016/j.molcel.2016.11.020 27984744 PMC5180613

[B127] Johnson E. L. Robinson D. G. Coller H. A. (2017). Widespread changes in mRNA stability contribute to quiescence-specific gene expression patterns in a fibroblast model of quiescence. *BMC Genomics* 18:123. 10.1186/s12864-017-3521-0 28143407 PMC5286691

[B128] Jorgensen S. Elvers I. Trelle M. B. Menzel T. Eskildsen M. Jensen O. N. (2007). The histone methyltransferase SET8 is required for S-phase progression. *J. Cell Biol.* 179 1337–1345. 10.1083/jcb.200706150 18166648 PMC2373509

[B129] Jorgensen S. Schotta G. Sorensen C. S. (2013). Histone H4 lysine 20 methylation: key player in epigenetic regulation of genomic integrity. *Nucleic Acids Res.* 41 2797–2806. 10.1093/nar/gkt012 23345616 PMC3597678

[B130] Julien E. Herr W. (2004). A switch in mitotic histone H4 lysine 20 methylation status is linked to M phase defects upon loss of HCF-1. *Mol. Cell* 14 713–725. 10.1016/j.molcel.2004.06.008 15200950

[B131] Kaelin W. G. Jr. McKnight S. L. (2013). Influence of metabolism on epigenetics and disease. *Cell* 153 56–69. 23540690 10.1016/j.cell.2013.03.004 PMC3775362

[B132] Kalakonda N. Fischle W. Boccuni P. Gurvich N. Hoya-Arias R. Zhao X. (2008). Histone H4 lysine 20 monomethylation promotes transcriptional repression by L3MBTL1. *Oncogene* 27 4293–4304. 10.1038/onc.2008.67 18408754 PMC2742506

[B133] Kalamakis G. Brune D. Ravichandran S. Bolz J. Fan W. Ziebell F. (2019). Quiescence modulates stem cell maintenance and regenerative capacity in the aging brain. *Cell* 176 1407–19.e14. 10.1016/j.cell.2019.01.040 30827680

[B134] Kalb R. Latwiel S. Baymaz H. I. Jansen P. W. Muller C. W. Vermeulen M. (2014). Histone H2A monoubiquitination promotes histone H3 methylation in Polycomb repression. *Nat. Struct. Mol. Biol.* 21 569–571. 24837194 10.1038/nsmb.2833

[B135] Kallingappa P. K. Turner P. M. Eichenlaub M. P. Green A. L. Oback F. C. Chibnall A. M. (2016). Quiescence loosens epigenetic constraints in bovine somatic cells and improves their reprogramming into totipotency. *Biol. Reprod.* 95:16. 10.1095/biolreprod.115.137109 27281704

[B136] Kang S. Long K. Wang S. Sada A. Tumbar T. (2020). Histone H3 K4/9/27 trimethylation levels affect wound healing and stem cell dynamics in adult skin. *Stem Cell Rep.* 14 34–48. 10.1016/j.stemcr.2019.11.007 31866458 PMC6962642

[B137] Kantidakis T. Saponaro M. Mitter R. Horswell S. Kranz A. Boeing S. (2016). Mutation of cancer driver MLL2 results in transcription stress and genome instability. *Genes Dev.* 30 408–420. 10.1101/gad.275453.115 26883360 PMC4762426

[B138] Kaya-Okur H. S. Wu S. J. Codomo C. A. Pledger E. S. Bryson T. D. Henikoff J. G. (2019). CUT&Tag for efficient epigenomic profiling of small samples and single cells. *Nat. Commun.* 10:1930. 10.1038/s41467-019-09982-5 PMC6488672 31036827

[B139] Kent D. Dykstra B. Eaves C. (2007). Isolation and assessment of long-term reconstituting hematopoietic stem cells from adult mouse bone marrow. *Curr. Protoc. Stem Cell Biol.* Chapter 2:Unit 2A.4. 10.1002/9780470151808.sc02a04s3 18785176

[B140] Kharchenko P. V. Alekseyenko A. A. Schwartz Y. B. Minoda A. Riddle N. C. Ernst J. (2011). Comprehensive analysis of the chromatin landscape in *Drosophila* melanogaster. *Nature* 471 480–485. 21179089 10.1038/nature09725 PMC3109908

[B141] Khoa L. T. P. Tsan Y. C. Mao F. Kremer D. M. Sajjakulnukit P. Zhang L. (2020). Histone acetyltransferase MOF blocks acquisition of quiescence in ground-state ESCs through activating fatty acid oxidation. *Cell Stem Cell* 27 441–458.e10. 10.1016/j.stem.2020.06.005 32610040 PMC7758074

[B142] Kim S. K. Jung I. Lee H. Kang K. Kim M. Jeong K. (2012). Human histone H3K79 methyltransferase DOT1L protein [corrected] binds actively transcribing RNA polymerase II to regulate gene expression. *J. Biol. Chem.* 287 39698–39709. 10.1074/jbc.M112.384057 23012353 PMC3501035

[B143] Kim T. D. Shin S. Berry W. L. Oh S. Janknecht R. (2012). The JMJD2A demethylase regulates apoptosis and proliferation in colon cancer cells. *J. Cell. Biochem.* 113 1368–1376. 10.1002/jcb.24009 22134899

[B144] Klose R. J. Yan Q. Tothova Z. Yamane K. Erdjument-Bromage H. Tempst P. (2007). The retinoblastoma binding protein RBP2 is an H3K4 demethylase. *Cell* 128 889–900. 17320163 10.1016/j.cell.2007.02.013

[B145] Knosp O. Talasz H. Puschendorf B. (1991). Histone acetylation and histone synthesis in mouse fibroblasts during quiescence and restimulation into S-phase. *Mol. Cell. Biochem.* 101 51–58. 10.1007/BF00238437 2011118

[B146] Kohlmaier A. Savarese F. Lachner M. Martens J. Jenuwein T. Wutz A. (2004). A chromosomal memory triggered by Xist regulates histone methylation in X inactivation. *PLoS Biol.* 2:E171. 10.1371/journal.pbio.0020171 15252442 PMC449785

[B147] Kolas N. K. Chapman J. R. Nakada S. Ylanko J. Chahwan R. Sweeney F. D. (2007). Orchestration of the DNA-damage response by the RNF8 ubiquitin ligase. *Science* 318 1637–1640. 18006705 10.1126/science.1150034 PMC2430610

[B148] Kornberg R. D. Lorch Y. (2020). Primary role of the nucleosome. *Mol. Cell* 79 371–375. 32763226 10.1016/j.molcel.2020.07.020

[B149] Kourmouli N. Jeppesen P. Mahadevhaiah S. Burgoyne P. Wu R. Gilbert D. M. (2004). Heterochromatin and tri-methylated lysine 20 of histone H4 in animals. *J. Cell Sci.* 117(Pt 12) 2491–2501. 15128874 10.1242/jcs.01238

[B150] Kurumizaka H. Kujirai T. Takizawa Y. (2021). Contributions of histone variants in nucleosome structure and function. *J. Mol. Biol.* 433:166678. 10.1016/j.jmb.2020.10.012 33065110

[B151] Kwon J. S. Everetts N. J. Wang X. Wang W. Della Croce K. Xing J. (2017). Controlling depth of cellular quiescence by an Rb-E2F network switch. *Cell Rep.* 20 3223–3235. 10.1016/j.celrep.2017.09.007 28954237 PMC6571029

[B152] Lachner M. O’Carroll D. Rea S. Mechtler K. Jenuwein T. (2001). Methylation of histone H3 lysine 9 creates a binding site for HP1 proteins. *Nature* 410 116–120. 11242053 10.1038/35065132

[B153] Lacoste N. Utley R. T. Hunter J. M. Poirier G. G. Cote J. (2002). Disruptor of telomeric silencing-1 is a chromatin-specific histone H3 methyltransferase. *J. Biol. Chem.* 277 30421–30424. 10.1074/jbc.C200366200 12097318

[B154] Laporte D. Courtout F. Tollis S. Sagot I. (2016). Quiescent *Saccharomyces cerevisiae* forms telomere hyperclusters at the nuclear membrane vicinity through a multifaceted mechanism involving Esc1, the Sir complex, and chromatin condensation. *Mol. Biol. Cell* 27 1875–1884. 10.1091/mbc.E16-01-0069 27122604 PMC4907721

[B155] Laporte D. Lebaudy A. Sahin A. Pinson B. Ceschin J. Daignan-Fornier B. (2011). Metabolic status rather than cell cycle signals control quiescence entry and exit. *J. Cell Biol.* 192 949–957. 10.1083/jcb.201009028 21402786 PMC3063145

[B156] Lauberth S. M. Nakayama T. Wu X. Ferris A. L. Tang Z. Hughes S. H. (2013). H3K4me3 interactions with TAF3 regulate preinitiation complex assembly and selective gene activation. *Cell* 152 1021–1036. 10.1016/j.cell.2013.01.052 23452851 PMC3588593

[B157] Lavarone E. Barbieri C. M. Pasini D. (2019). Dissecting the role of H3K27 acetylation and methylation in PRC2 mediated control of cellular identity. *Nat. Commun.* 10:1679. 10.1038/s41467-019-09624-w 30976011 PMC6459869

[B158] Lee H. N. Mitra M. Bosompra O. Corney D. C. Johnson E. L. Rashed N. (2018). RECK isoforms have opposing effects on cell migration. *Mol. Biol. Cell* 29 1825–1838. 29874120 10.1091/mbc.E17-12-0708 PMC6085827

[B159] Lee J. Kang S. Lilja K. C. Colletier K. J. Scheitz C. J. Zhang Y. V. (2016). Signalling couples hair follicle stem cell quiescence with reduced histone H3 K4/K9/K27me3 for proper tissue homeostasis. *Nat. Commun.* 7:11278. 10.1038/ncomms11278 27080563 PMC4835553

[B160] Lee S. Oh S. Jeong K. Jo H. Choi Y. Seo H. D. (2018). Dot1 regulates nucleosome dynamics by its inherent histone chaperone activity in yeast. *Nat. Commun.* 9:240. 10.1038/s41467-017-02759-8 29339748 PMC5770421

[B161] Legesse-Miller A. Raitman I. Haley E. M. Liao A. Sun L. L. Wang D. J. (2012). Quiescent fibroblasts are protected from proteasome inhibition-mediated toxicity. *Mol. Biol. Cell* 23 3566–3581. 10.1091/mbc.E12-03-0192 22875985 PMC3442405

[B162] Lemons J. M. Feng X. J. Bennett B. D. Legesse-Miller A. Johnson E. L. Raitman I. (2010). Quiescent fibroblasts exhibit high metabolic activity. *PLoS Biol.* 8:e1000514. 10.1371/journal.pbio.1000514 21049082 PMC2958657

[B163] Lesch B. J. Dokshin G. A. Young R. A. McCarrey J. R. Page D. C. (2013). A set of genes critical to development is epigenetically poised in mouse germ cells from fetal stages through completion of meiosis. *Proc. Natl. Acad. Sci. U.S.A.* 110 16061–16066. 10.1073/pnas.1315204110 24043772 PMC3791702

[B164] Li J. Mahata B. Escobar M. Goell J. Wang K. Khemka P. (2021). Programmable human histone phosphorylation and gene activation using a CRISPR/Cas9-based chromatin kinase. *Nat. Commun.* 12:896. 10.1038/s41467-021-21188-2 33563994 PMC7873277

[B165] Li L. Bhatia R. (2011). Stem cell quiescence. *Clin. Cancer Res.* 17 4936–4941. 21593194 10.1158/1078-0432.CCR-10-1499 PMC3410675

[B166] Li L. Clevers H. (2010). Coexistence of quiescent and active adult stem cells in mammals. *Science* 327 542–545. 20110496 10.1126/science.1180794 PMC4105182

[B167] Lieberman-Aiden E. van Berkum N. L. Williams L. Imakaev M. Ragoczy T. Telling A. (2009). Comprehensive mapping of long-range interactions reveals folding principles of the human genome. *Science* 326 289–293. 10.1126/science.1181369 19815776 PMC2858594

[B168] Lien W. H. Guo X. Polak L. Lawton L. N. Young R. A. Zheng D. (2011). Genome-wide maps of histone modifications unwind in vivo chromatin states of the hair follicle lineage. *Cell Stem Cell* 9 219–232. 10.1016/j.stem.2011.07.015 21885018 PMC3166618

[B169] Liokatis S. Stutzer A. Elsasser S. J. Theillet F. X. Klingberg R. van Rossum B. (2012). Phosphorylation of histone H3 Ser10 establishes a hierarchy for subsequent intramolecular modification events. *Nat. Struct. Mol. Biol.* 19 819–823. 10.1038/nsmb.2310 22796964

[B170] Liu L. Cheung T. H. Charville G. W. Hurgo B. M. Leavitt T. Shih J. (2013). Chromatin modifications as determinants of muscle stem cell quiescence and chronological aging. *Cell Rep.* 4 189–204. 10.1016/j.celrep.2013.05.043 23810552 PMC4103025

[B171] Liu Y. Chen S. Wang S. Soares F. Fischer M. Meng F. (2017). Transcriptional landscape of the human cell cycle. *Proc. Natl. Acad. Sci. U.S.A.* 114 3473–3478. 28289232 10.1073/pnas.1617636114 PMC5380023

[B172] Lomberk G. Bensi D. Fernandez-Zapico M. E. Urrutia R. (2006). Evidence for the existence of an HP1-mediated subcode within the histone code. *Nat. Cell Biol.* 8 407–415. 10.1038/ncb1383 16531993

[B173] Lu X. Simon M. D. Chodaparambil J. V. Hansen J. C. Shokat K. M. Luger K. (2008). The effect of H3K79 dimethylation and H4K20 trimethylation on nucleosome and chromatin structure. *Nat. Struct. Mol. Biol.* 15 1122–1124. 10.1038/nsmb.1489 18794842 PMC2648974

[B174] Luo M. Jeong M. Sun D. Park H. J. Rodriguez B. A. Xia Z. (2015). Long non-coding RNAs control hematopoietic stem cell function. *Cell Stem Cell* 16 426–438. 25772072 10.1016/j.stem.2015.02.002 PMC4388783

[B175] Lynch M. D. Watt F. M. (2018). Fibroblast heterogeneity: implications for human disease. *J. Clin. Invest.* 128 26–35. 29293096 10.1172/JCI93555 PMC5749540

[B176] Ma D. K. Bonaguidi M. A. Ming G. L. Song H. (2009). Adult neural stem cells in the mammalian central nervous system. *Cell Res.* 19 672–682. 19436263 10.1038/cr.2009.56 PMC2738865

[B177] Machida S. Takizawa Y. Ishimaru M. Sugita Y. Sekine S. Nakayama J. I. (2018). Structural basis of heterochromatin formation by human HP1. *Mol. Cell* 69 385–397.e8. 29336876 10.1016/j.molcel.2017.12.011

[B178] Mailand N. Bekker-Jensen S. Faustrup H. Melander F. Bartek J. Lukas C. (2007). RNF8 ubiquitylates histones at DNA double-strand breaks and promotes assembly of repair proteins. *Cell* 131 887–900. 18001824 10.1016/j.cell.2007.09.040

[B179] Maki K. Nava M. M. Villeneuve C. Chang M. Furukawa K. S. Ushida T. (2021). Hydrostatic pressure prevents chondrocyte differentiation through heterochromatin remodeling. *J. Cell Sci.* 134:jcs247643. 10.1242/jcs.247643 PMC7860130 33310912

[B180] Maksimovic I. David Y. (2021). Non-enzymatic covalent modifications as a new chapter in the histone code. *Trends Biochem. Sci.* 46 718–730. 33965314 10.1016/j.tibs.2021.04.004 PMC8364488

[B181] Marescal O. Cheeseman I. M. (2020). Cellular mechanisms and regulation of quiescence. *Dev. Cell* 55 259–271. 33171109 10.1016/j.devcel.2020.09.029 PMC7665062

[B182] Margaritis T. Holstege F. C. (2008). Poised RNA polymerase II gives pause for thought. *Cell* 133 581–584. 10.1016/j.cell.2008.04.027 18485867

[B183] Marguerat S. Schmidt A. Codlin S. Chen W. Aebersold R. Bahler J. (2012). Quantitative analysis of fission yeast transcriptomes and proteomes in proliferating and quiescent cells. *Cell* 151 671–683. 10.1016/j.cell.2012.09.019 23101633 PMC3482660

[B184] Margueron R. Reinberg D. (2011). The Polycomb complex PRC2 and its mark in life. *Nature* 469 343–349. 21248841 10.1038/nature09784 PMC3760771

[B185] Margueron R. Justin N. Ohno K. Sharpe M. L. Son J. Drury W. J. III (2009). Role of the polycomb protein EED in the propagation of repressive histone marks. *Nature* 461 762–767. 10.1038/nature08398 19767730 PMC3772642

[B186] Margueron R. Li G. Sarma K. Blais A. Zavadil J. Woodcock C. L. (2008). Ezh1 and Ezh2 maintain repressive chromatin through different mechanisms. *Mol. Cell* 32 503–518. 19026781 10.1016/j.molcel.2008.11.004 PMC3641558

[B187] Marion R. M. Schotta G. Ortega S. Blasco M. A. (2011). Suv4-20h abrogation enhances telomere elongation during reprogramming and confers a higher tumorigenic potential to iPS cells. *PLoS One* 6:e25680. 10.1371/journal.pone.0025680 22022429 PMC3192133

[B188] Martens J. H. O’Sullivan R. J. Braunschweig U. Opravil S. Radolf M. Steinlein P. (2005). The profile of repeat-associated histone lysine methylation states in the mouse epigenome. *EMBO J.* 24 800–812. 10.1038/sj.emboj.7600545 15678104 PMC549616

[B189] Martinez M. J. Roy S. Archuletta A. B. Wentzell P. D. Anna-Arriola S. S. Rodriguez A. L. (2004). Genomic analysis of stationary-phase and exit in Saccharomyces cerevisiae: gene expression and identification of novel essential genes. *Mol. Biol. Cell* 15 5295–5305. 10.1091/mbc.e03-11-0856 15456898 PMC532011

[B190] Martinez-Reyes I. Chandel N. S. (2020). Mitochondrial TCA cycle metabolites control physiology and disease. *Nat. Commun.* 11:102. 10.1038/s41467-019-13668-3 PMC6941980 31900386

[B191] Martynoga B. Mateo J. L. Zhou B. Andersen J. Achimastou A. Urban N. (2013). Epigenomic enhancer annotation reveals a key role for NFIX in neural stem cell quiescence. *Genes Dev.* 27 1769–1786. 10.1101/gad.216804.113 23964093 PMC3759694

[B192] Maurer-Stroh S. Dickens N. J. Hughes-Davies L. Kouzarides T. Eisenhaber F. Ponting C. P. (2003). The Tudor domain ‘royal family’: Tudor, plant Agenet, chromo, PWWP and MBT domains. *Trends Biochem. Sci.* 28 69–74. 10.1016/S0968-0004(03)00004-5 12575993

[B193] Maya-Miles D. Andujar E. Perez-Alegre M. Murillo-Pineda M. Barrientos-Moreno M. Cabello-Lobato M. J. (2019). Crosstalk between chromatin structure, cohesin activity and transcription. *Epigenet. Chromatin* 12 47. 10.1186/s13072-019-0293-6 31331360 PMC6647288

[B194] McKnight J. N. Boerma J. W. Breeden L. L. Tsukiyama T. (2015). Global promoter targeting of a conserved lysine deacetylase for transcriptional shutoff during quiescence entry. *Mol. Cell* 59 732–743. 10.1016/j.molcel.2015.07.014 26300265 PMC4560983

[B195] McMahon S. B. Wood M. A. Cole M. D. (2000). The essential cofactor TRRAP recruits the histone acetyltransferase hGCN5 to c-Myc. *Mol. Cell. Biol.* 20 556–562. 10.1128/MCB.20.2.556-562.2000 10611234 PMC85131

[B196] Mehta I. S. Amira M. Harvey A. J. Bridger J. M. (2010). Rapid chromosome territory relocation by nuclear motor activity in response to serum removal in primary human fibroblasts. *Genome Biol.* 11:R5. 10.1186/gb-2010-11-1-r5 20070886 PMC2847717

[B197] Meng F. Stamms K. Bennewitz R. Green A. Oback F. Turner P. (2020). Targeted histone demethylation improves somatic cell reprogramming into cloned blastocysts but not postimplantation bovine concepti†. *Biol. Reprod.* 103 114–125. 10.1093/biolre/ioaa053 32318688

[B198] Mews P. Zee B. M. Liu S. Donahue G. Garcia B. A. Berger S. L. (2014). Histone methylation has dynamics distinct from those of histone acetylation in cell cycle reentry from quiescence. *Mol. Cell. Biol.* 34 3968–3980. 10.1128/MCB.00763-14 25154414 PMC4386454

[B199] Michan S. Sinclair D. (2007). Sirtuins in mammals: insights into their biological function. *Biochem. J.* 404 1–13. 17447894 10.1042/BJ20070140 PMC2753453

[B200] Mikkelsen T. S. Ku M. Jaffe D. B. Issac B. Lieberman E. Giannoukos G. (2007). Genome-wide maps of chromatin state in pluripotent and lineage-committed cells. *Nature* 448 553–560. 10.1038/nature06008 17603471 PMC2921165

[B201] Miles S. Bradley G. T. Breeden L. L. (2021). The budding yeast transition to quiescence. *Yeast* 38 30–38. 33350501 10.1002/yea.3546

[B202] Miles S. Li L. H. Melville Z. Breeden L. L. (2019). Ssd1 and the cell wall integrity pathway promote entry, maintenance, and recovery from quiescence in budding yeast. *Mol. Biol. Cell.* 30 2205–2217. 10.1091/mbc.E19-04-0190 31141453 PMC6743469

[B203] Milne T. A. Zhao K. Hess J. L. (2009). Chromatin immunoprecipitation (ChIP) for analysis of histone modifications and chromatin-associated proteins. *Methods Mol. Biol.* 538 409–423. 19277579 10.1007/978-1-59745-418-6_21 PMC4157307

[B204] Mitra M. Ho L. D. Coller H. A. (2018a). An in vitro model of cellular quiescence in primary human dermal fibroblasts. *Methods Mol. Biol.* 1686 27–47. 10.1007/978-1-4939-7371-2_2 29030810 PMC5718883

[B205] Mitra M. Johnson E. L. Swamy V. S. Nersesian L. E. Corney D. C. Robinson D. G. (2018b). Alternative polyadenylation factors link cell cycle to migration. *Genome Biol.* 19:176. 10.1186/s13059-018-1551-9 30360761 PMC6203201

[B206] Mochida S. Yanagida M. (2006). Distinct modes of DNA damage response in S. pombe G0 and vegetative cells. *Genes Cells* 11 13–27. 10.1111/j.1365-2443.2005.00917.x 16371129

[B207] Montoya-Durango D. E. Liu Y. Teneng I. Kalbfleisch T. Lacy M. E. Steffen M. C. (2009). Epigenetic control of mammalian LINE-1 retrotransposon by retinoblastoma proteins. *Mutat. Res.* 665 20–28. 10.1016/j.mrfmmm.2009.02.011 19427507 PMC3418809

[B208] Morgan M. A. J. Shilatifard A. (2020). Reevaluating the roles of histone-modifying enzymes and their associated chromatin modifications in transcriptional regulation. *Nat. Genet.* 52 1271–1281. 10.1038/s41588-020-00736-4 33257899

[B209] Morris S. A. Shibata Y. Noma K. Tsukamoto Y. Warren E. Temple B. (2005). Histone H3 K36 methylation is associated with transcription elongation in *Schizosaccharomyces* pombe. *Eukaryot. Cell* 4 1446–1454. 10.1128/EC.4.8.1446-1454.2005 16087749 PMC1214526

[B210] Morrish F. Noonan J. Perez-Olsen C. Gafken P. R. Fitzgibbon M. Kelleher J. (2010). Myc-dependent mitochondrial generation of acetyl-CoA contributes to fatty acid biosynthesis and histone acetylation during cell cycle entry. *J. Biol. Chem.* 285 36267–36274. 10.1074/jbc.M110.141606 20813845 PMC2978554

[B211] Mueller D. Bach C. Zeisig D. Garcia-Cuellar M. P. Monroe S. Sreekumar A. (2007). A role for the MLL fusion partner ENL in transcriptional elongation and chromatin modification. *Blood* 110 4445–4454. 17855633 10.1182/blood-2007-05-090514 PMC2234781

[B212] Muhl L. Genove G. Leptidis S. Liu J. He L. Mocci G. (2020). Single-cell analysis uncovers fibroblast heterogeneity and criteria for fibroblast and mural cell identification and discrimination. *Nat. Commun.* 11:3953. 10.1038/s41467-020-17740-1 PMC7414220 32769974

[B213] Murton B. L. Chin W. L. Ponting C. P. Itzhaki L. S. (2010). Characterising the binding specificities of the subunits associated with the KMT2/Set1 histone lysine methyltransferase. *J. Mol. Biol.* 398 481–488. 10.1016/j.jmb.2010.03.036 20347846

[B214] Nakamura T. Pluskal T. Nakaseko Y. Yanagida M. (2012). Impaired coenzyme A synthesis in fission yeast causes defective mitosis, quiescence-exit failure, histone hypoacetylation and fragile DNA. *Open Biol.* 2:120117. 10.1098/rsob.120117 23091701 PMC3472395

[B215] Nakamura-Ishizu A. Takizawa H. Suda T. (2014). The analysis, roles and regulation of quiescence in hematopoietic stem cells. *Development* 141 4656–4666. 25468935 10.1242/dev.106575

[B216] Nasmyth K. (2011). Cohesin: a catenase with separate entry and exit gates? *Nat. Cell Biol.* 13 1170–1177. 10.1038/ncb2349 21968990

[B217] Neiman A. M. (2011). Sporulation in the budding yeast *Saccharomyces cerevisiae*. *Genetics* 189 737–765. 22084423 10.1534/genetics.111.127126 PMC3213374

[B218] Nelson D. M. Jaber-Hijazi F. Cole J. J. Robertson N. A. Pawlikowski J. S. Norris K. T. (2016). Mapping H4K20me3 onto the chromatin landscape of senescent cells indicates a function in control of cell senescence and tumor suppression through preservation of genetic and epigenetic stability. *Genome Biol.* 17:158. 10.1186/s13059-016-1017-x 27457071 PMC4960804

[B219] Ngubo M. Kemp G. Patterton H. G. (2011). Nano-electrospray tandem mass spectrometric analysis of the acetylation state of histones H3 and H4 in stationary phase in *Saccharomyces cerevisiae*. *BMC Biochem.* 12:34. 10.1186/1471-2091-12-34 21726436 PMC3136420

[B220] Nguyen A. T. Zhang Y. (2011). The diverse functions of Dot1 and H3K79 methylation. *Genes Dev.* 25 1345–1358. 21724828 10.1101/gad.2057811 PMC3134078

[B221] Ninova M. Fejes Toth K. Aravin A. A. (2019). The control of gene expression and cell identity by H3K9 trimethylation. *Development* 146:dev181180. 10.1242/dev.181180 PMC6803365 31540910

[B222] Nishioka K. Rice J. C. Sarma K. Erdjument-Bromage H. Werner J. Wang Y. (2002b). PR-Set7 is a nucleosome-specific methyltransferase that modifies lysine 20 of histone H4 and is associated with silent chromatin. *Mol. Cell* 9 1201–1213. 10.1016/s1097-2765(02)00548-8 12086618

[B223] Nishioka K. Chuikov S. Sarma K. Erdjument-Bromage H. Allis C. D. Tempst P. (2002a). Set9, a novel histone H3 methyltransferase that facilitates transcription by precluding histone tail modifications required for heterochromatin formation. *Genes Dev.* 16 479–489. 10.1101/gad.967202 11850410 PMC155346

[B224] Norton V. G. Imai B. S. Yau P. Bradbury E. M. (1989). Histone acetylation reduces nucleosome core particle linking number change. *Cell* 57 449–457. 2541913 10.1016/0092-8674(89)90920-3

[B225] Nowak J. A. Polak L. Pasolli H. A. Fuchs E. (2008). Hair follicle stem cells are specified and function in early skin morphogenesis. *Cell Stem Cell* 3 33–43. 10.1016/j.stem.2008.05.009 18593557 PMC2877596

[B226] Nurse P. Bissett Y. (1981). Gene required in G1 for commitment to cell cycle and in G2 for control of mitosis in fission yeast. *Nature* 292 558–560. 7254352 10.1038/292558a0

[B227] Obernier K. Cebrian-Silla A. Thomson M. Parraguez J. I. Anderson R. Guinto C. (2018). Adult neurogenesis is sustained by symmetric self-renewal and differentiation. *Cell Stem Cell* 22 221–234.e8. 29395056 10.1016/j.stem.2018.01.003 PMC5802882

[B228] Oda H. Okamoto I. Murphy N. Chu J. Price S. M. Shen M. M. (2009). Monomethylation of histone H4-lysine 20 is involved in chromosome structure and stability and is essential for mouse development. *Mol. Cell. Biol.* 29 2278–2295. 10.1128/MCB.01768-08 19223465 PMC2663305

[B229] O’Geen H. Echipare L. Farnham P. J. (2011). Using ChIP-seq technology to generate high-resolution profiles of histone modifications. *Methods Mol. Biol.* 791 265–286. 21913086 10.1007/978-1-61779-316-5_20 PMC4151291

[B230] Oya E. Durand-Dubief M. Cohen A. Maksimov V. Schurra C. Nakayama J. I. (2019). Leo1 is essential for the dynamic regulation of heterochromatin and gene expression during cellular quiescence. *Epigenet. Chromatin* 12:45. 10.1186/s13072-019-0292-7 31315658 PMC6636030

[B231] Park S. Y. Kim J. S. (2020). A short guide to histone deacetylases including recent progress on class II enzymes. *Exp. Mol. Med.* 52 204–212. 10.1038/s12276-020-0382-4 32071378 PMC7062823

[B232] Pesavento J. J. Yang H. Kelleher N. L. Mizzen C. A. (2008). Certain and progressive methylation of histone H4 at lysine 20 during the cell cycle. *Mol. Cell. Biol.* 28 468–486. 10.1128/MCB.01517-07 17967882 PMC2223297

[B233] Pradeepa M. M. Grimes G. R. Kumar Y. Olley G. Taylor G. C. Schneider R. (2016). Histone H3 globular domain acetylation identifies a new class of enhancers. *Nat. Genet.* 48 681–686. 10.1038/ng.3550 27089178 PMC4886833

[B234] Prakash K. Fournier D. (2018). Evidence for the implication of the histone code in building the genome structure. *Biosystems* 164 49–59. 10.1016/j.biosystems.2017.11.005 29158132

[B235] Radonjic M. Andrau J. C. Lijnzaad P. Kemmeren P. Kockelkorn T. T. van Leenen D. (2005). Genome-wide analyses reveal RNA polymerase II located upstream of genes poised for rapid response upon S. cerevisiae stationary phase exit. *Mol. Cell* 18 171–183. 10.1016/j.molcel.2005.03.010 15837421

[B236] Rando O. J. (2012). Combinatorial complexity in chromatin structure and function: revisiting the histone code. *Curr. Opin. Genet. Dev.* 22 148–155. 10.1016/j.gde.2012.02.013 22440480 PMC3345062

[B237] Rao S. S. Huntley M. H. Durand N. C. Stamenova E. K. Bochkov I. D. Robinson J. T. (2014). A 3D map of the human genome at kilobase resolution reveals principles of chromatin looping. *Cell* 159 1665–1680. 25497547 10.1016/j.cell.2014.11.021 PMC5635824

[B238] Rawlings J. S. Gatzka M. Thomas P. G. Ihle J. N. (2011). Chromatin condensation via the condensin II complex is required for peripheral T-cell quiescence. *EMBO J.* 30 263–276. 10.1038/emboj.2010.314 21169989 PMC3025460

[B239] Rhodes C. T. Sandstrom R. S. Huang S. A. Wang Y. Schotta G. Berger M. S. (2016). Cross-species analyses unravel the complexity of H3K27me3 and H4K20me3 in the context of neural stem progenitor cells. *Neuroepigenetics* 6 10–25. 10.1016/j.nepig.2016.04.001 27429906 PMC4941106

[B240] Rice J. C. Nishioka K. Sarma K. Steward R. Reinberg D. Allis C. D. (2002). Mitotic-specific methylation of histone H4 Lys 20 follows increased PR-Set7 expression and its localization to mitotic chromosomes. *Genes Dev.* 16 2225–2230. 10.1101/gad.1014902 12208845 PMC186671

[B241] Rickels R. Herz H. M. Sze C. C. Cao K. Morgan M. A. Collings C. K. (2017). Histone H3K4 monomethylation catalyzed by Trr and mammalian COMPASS-like proteins at enhancers is dispensable for development and viability. *Nat. Genet.* 49 1647–1653. 10.1038/ng.3965 28967912 PMC5663216

[B242] Riddle N. C. Minoda A. Kharchenko P. V. Alekseyenko A. A. Schwartz Y. B. Tolstorukov M. Y. (2011). Plasticity in patterns of histone modifications and chromosomal proteins in *Drosophila* heterochromatin. *Genome Res.* 21 147–163. 10.1101/gr.110098.110 21177972 PMC3032919

[B243] Rinn J. L. Kertesz M. Wang J. K. Squazzo S. L. Xu X. Brugmann S. A. (2007). Functional demarcation of active and silent chromatin domains in human HOX loci by noncoding RNAs. *Cell* 129 1311–1323. 10.1016/j.cell.2007.05.022 17604720 PMC2084369

[B244] Rittershaus E. S. Baek S. H. Sassetti C. M. (2013). The normalcy of dormancy: common themes in microbial quiescence. *Cell Host Microbe* 13 643–651. 10.1016/j.chom.2013.05.012 23768489 PMC3743100

[B245] Rodgers J. T. King K. Y. Brett J. O. Cromie M. J. Charville G. W. Maguire K. K. (2014). mTORC1 controls the adaptive transition of quiescent stem cells from G0 to G(Alert). *Nature* 510 393–396. 10.1038/nature13255 24870234 PMC4065227

[B246] Rodriguez C. N. Nguyen H. (2018). Identifying quiescent stem cells in hair follicles. *Methods Mol. Biol.* 1686 137–147. 29030818 10.1007/978-1-4939-7371-2_10 PMC5697140

[B247] Rogakou E. P. Pilch D. R. Orr A. H. Ivanova V. S. Bonner W. M. (1998). DNA double-stranded breaks induce histone H2AX phosphorylation on serine 139. *J. Biol. Chem.* 273 5858–5868. 9488723 10.1074/jbc.273.10.5858

[B248] Roudier F. Ahmed I. Berard C. Sarazin A. Mary-Huard T. Cortijo S. (2011). Integrative epigenomic mapping defines four main chromatin states in *Arabidopsis*. *EMBO J.* 30 1928–1938. 10.1038/emboj.2011.103 21487388 PMC3098477

[B249] Ruthenburg A. J. Li H. Milne T. A. Dewell S. McGinty R. K. Yuen M. (2011). Recognition of a mononucleosomal histone modification pattern by BPTF via multivalent interactions. *Cell* 145 692–706. 10.1016/j.cell.2011.03.053 21596426 PMC3135172

[B250] Rutledge M. T. Russo M. Belton J. M. Dekker J. Broach J. R. (2015). The yeast genome undergoes significant topological reorganization in quiescence. *Nucleic Acids Res.* 43 8299–8313. 10.1093/nar/gkv723 26202961 PMC4787801

[B251] Ryall J. G. Dell’Orso S. Derfoul A. Juan A. Zare H. Feng X. (2015). The NAD(+)-dependent SIRT1 deacetylase translates a metabolic switch into regulatory epigenetics in skeletal muscle stem cells. *Cell Stem Cell* 16 171–183. 10.1016/j.stem.2014.12.004 25600643 PMC4320668

[B252] Sagot I. Laporte D. (2019a). Quiescence, an individual journey. *Curr. Genet.* 65 695–699. 30649583 10.1007/s00294-018-00928-w

[B253] Sagot I. Laporte D. (2019b). The cell biology of quiescent yeast - a diversity of individual scenarios. *J. Cell Sci.* 132:jcs213025. 10.1242/jcs.213025 30602574

[B254] Sajiki K. Hatanaka M. Nakamura T. Takeda K. Shimanuki M. Yoshida T. (2009). Genetic control of cellular quiescence in S. pombe. *J. Cell Sci.* 122(Pt 9) 1418–1429. 10.1242/jcs.046466 19366728

[B255] Saksouk N. Simboeck E. Dejardin J. (2015). Constitutive heterochromatin formation and transcription in mammals. *Epigenet. Chromatin* 8:3. 10.1186/1756-8935-8-3 PMC4363358 25788984

[B256] Sandmeier J. J. French S. Osheim Y. Cheung W. L. Gallo C. M. Beyer A. L. (2002). RPD3 is required for the inactivation of yeast ribosomal DNA genes in stationary phase. *EMBO J.* 21 4959–4968. 10.1093/emboj/cdf498 12234935 PMC126294

[B257] Sang L. Coller H. A. (2009). Fear of commitment: Hes1 protects quiescent fibroblasts from irreversible cellular fates. *Cell Cycle* 8 2161–2167. 10.4161/cc.8.14.9104 19587546

[B258] Sang L. Roberts J. M. Coller H. A. (2010). Hijacking HES1: how tumors co-opt the anti-differentiation strategies of quiescent cells. *Trends Mol. Med.* 16 17–26. 10.1016/j.molmed.2009.11.001 20022559 PMC2864914

[B259] Schafer G. McEvoy C. R. Patterton H. G. (2008). The *Saccharomyces cerevisiae* linker histone Hho1p is essential for chromatin compaction in stationary phase and is displaced by transcription. *Proc. Natl. Acad. Sci. U.S.A.* 105 14838–14843. 10.1073/pnas.0806337105 18799740 PMC2567454

[B260] Schmitges F. W. Prusty A. B. Faty M. Stutzer A. Lingaraju G. M. Aiwazian J. (2011). Histone methylation by PRC2 is inhibited by active chromatin marks. *Mol. Cell* 42 330–341. 21549310 10.1016/j.molcel.2011.03.025

[B261] Schneider C. King R. M. Philipson L. (1988). Genes specifically expressed at growth arrest of mammalian cells. *Cell* 54 787–793. 3409319 10.1016/s0092-8674(88)91065-3

[B262] Schotta G. Lachner M. Sarma K. Ebert A. Sengupta R. Reuter G. (2004). A silencing pathway to induce H3-K9 and H4-K20 trimethylation at constitutive heterochromatin. *Genes Dev.* 18 1251–1262. 10.1101/gad.300704 15145825 PMC420351

[B263] Schotta G. Sengupta R. Kubicek S. Malin S. Kauer M. Callen E. (2008). A chromatin-wide transition to H4K20 monomethylation impairs genome integrity and programmed DNA rearrangements in the mouse. *Genes Dev.* 22 2048–2061. 10.1101/gad.476008 18676810 PMC2492754

[B264] Setterfield G. Hall R. Bladon T. Little J. Kaplan J. G. (1983). Changes in structure and composition of lymphocyte nuclei during mitogenic stimulation. *J. Ultrastruct. Res.* 82 264–282. 6188858 10.1016/s0022-5320(83)80014-8

[B265] Shi X. Kachirskaia I. Walter K. L. Kuo J. H. Lake A. Davrazou F. (2007). Proteome-wide analysis in *Saccharomyces cerevisiae* identifies several PHD fingers as novel direct and selective binding modules of histone H3 methylated at either lysine 4 or lysine 36. *J. Biol. Chem.* 282 2450–2455. 17142463 10.1074/jbc.C600286200 PMC2735445

[B266] Shimada M. Niida H. Zineldeen D. H. Tagami H. Tanaka M. Saito H. (2008). Chk1 is a histone H3 threonine 11 kinase that regulates DNA damage-induced transcriptional repression. *Cell* 132 221–232. 18243098 10.1016/j.cell.2007.12.013

[B267] Shogren-Knaak M. Ishii H. Sun J. M. Pazin M. J. Davie J. R. Peterson C. L. (2006). Histone H4-K16 acetylation controls chromatin structure and protein interactions. *Science* 311 844–847. 10.1126/science.1124000 16469925

[B268] Sieburg H. B. Cho R. H. Dykstra B. Uchida N. Eaves C. J. Muller-Sieburg C. E. (2006). The hematopoietic stem compartment consists of a limited number of discrete stem cell subsets. *Blood* 107 2311–2316. 10.1182/blood-2005-07-2970 16291588 PMC1456063

[B269] Sims J. K. Houston S. I. Magazinnik T. Rice J. C. (2006). A trans-tail histone code defined by monomethylated H4 Lys-20 and H3 Lys-9 demarcates distinct regions of silent chromatin. *J. Biol. Chem.* 281 12760–12766. 10.1074/jbc.M513462200 16517599

[B270] Singer M. S. Kahana A. Wolf A. J. Meisinger L. L. Peterson S. E. Goggin C. (1998). Identification of high-copy disruptors of telomeric silencing in *Saccharomyces cerevisiae*. *Genetics* 150 613–632. 10.1093/genetics/150.2.613 9755194 PMC1460361

[B271] Smeenk G. Mailand N. (2016). Writers, readers, and erasers of histone ubiquitylation in DNA double-strand break repair. *Front. Genet.* 7:122. 10.3389/fgene.2016.00122 27446204 PMC4923129

[B272] So W. K. Cheung T. H. (2018). Molecular regulation of cellular quiescence: a perspective from adult stem cells and its niches. *Methods Mol. Biol.* 1686 1–25. 10.1007/978-1-4939-7371-2_1 29030809

[B273] Sorrell J. M. Caplan A. I. (2004). Fibroblast heterogeneity: more than skin deep. *J. Cell Sci.* 117(Pt 5) 667–675. 14754903 10.1242/jcs.01005

[B274] Soshnev A. A. Josefowicz S. Z. Allis C. D. (2016). Greater than the sum of parts: complexity of the dynamic epigenome. *Mol. Cell* 62 681–694. 27259201 10.1016/j.molcel.2016.05.004 PMC4898265

[B275] Spain M. M. Swygert S. G. Tsukiyama T. (2018). Preparation and analysis of *Saccharomyces cerevisiae* quiescent cells. *Methods Mol. Biol.* 1686 125–135. 29030817 10.1007/978-1-4939-7371-2_9

[B276] Spencer S. L. Cappell S. D. Tsai F. C. Overton K. W. Wang C. L. Meyer T. (2013). The proliferation-quiescence decision is controlled by a bifurcation in CDK2 activity at mitotic exit. *Cell* 155 369–383. 10.1016/j.cell.2013.08.062 24075009 PMC4001917

[B277] Srivastava S. Gala H. P. Mishra R. K. Dhawan J. (2018). Distinguishing states of arrest: genome-wide descriptions of cellular quiescence using ChIP-Seq and RNA-Seq analysis. *Methods Mol. Biol.* 1686 215–239. 10.1007/978-1-4939-7371-2_16 29030824

[B278] Steele-Perkins G. Fang W. Yang X. H. Van Gele M. Carling T. Gu J. (2001). Tumor formation and inactivation of RIZ1, an Rb-binding member of a nuclear protein-methyltransferase superfamily. *Genes Dev.* 15 2250–2262. 10.1101/gad.870101 11544182 PMC312773

[B279] Stender J. D. Pascual G. Liu W. Kaikkonen M. U. Do K. Spann N. J. (2012). Control of proinflammatory gene programs by regulated trimethylation and demethylation of histone H4K20. *Mol. Cell* 48 28–38. 10.1016/j.molcel.2012.07.020 22921934 PMC3472359

[B280] Strahl B. D. Allis C. D. (2000). The language of covalent histone modifications. *Nature* 403 41–45. 10638745 10.1038/47412

[B281] Stucki M. Clapperton J. A. Mohammad D. Yaffe M. B. Smerdon S. J. Jackson S. P. (2005). MDC1 directly binds phosphorylated histone H2AX to regulate cellular responses to DNA double-strand breaks. *Cell* 123 1213–1226. 16377563 10.1016/j.cell.2005.09.038

[B282] Su S. S. Tanaka Y. Samejima I. Tanaka K. Yanagida M. (1996). A nitrogen starvation-induced dormant G0 state in fission yeast: the establishment from uncommitted G1 state and its delay for return to proliferation. *J. Cell Sci.* 109(Pt 6) 1347–1357. 8799823 10.1242/jcs.109.6.1347

[B283] Suh E. J. Remillard M. Y. Legesse-Miller A. Johnson E. L. Lemons J. M. Chapman T. R. (2012). A microRNA network regulates proliferative timing and extracellular matrix synthesis during cellular quiescence in fibroblasts. *Genome Biol.* 13:R121. 10.1186/gb-2012-13-12-r121 23259597 PMC3924601

[B284] Sun D. Buttitta L. (2017). States of G(0) and the proliferation-quiescence decision in cells, tissues and during development. *Int. J. Dev. Biol.* 61 357–366. 10.1387/ijdb.160343LB 28695955

[B285] Svensson V. Vento-Tormo R. Teichmann S. A. (2018). Exponential scaling of single-cell RNA-seq in the past decade. *Nat. Protoc.* 13 599–604. 10.1038/nprot.2017.149 29494575

[B286] Swygert S. G. Kim S. Wu X. Fu T. Hsieh T. H. Rando O. J. (2019). Condensin-Dependent chromatin compaction represses transcription globally during quiescence. *Mol. Cell* 73 533–546.e4. 30595435 10.1016/j.molcel.2018.11.020 PMC6368455

[B287] Swygert S. G. Lin D. Portillo-Ledesma S. Lin P.-Y. Hunt D. R. Kao C.-F. (2021). Chromatin fiber folding represses transcription and loop extrusion in quiescent cells. *bioRxiv* [preprint] 10.1101/2020.11.24.396713 PMC8598167 34734806

[B288] Takeda K. Yanagida M. (2010). In quiescence of fission yeast, autophagy and the proteasome collaborate for mitochondrial maintenance and longevity. *Autophagy* 6 564–565. 20418666 10.4161/auto.6.4.11948

[B289] Takei Y. Yun J. Zheng S. Ollikainen N. Pierson N. White J. (2021). Integrated spatial genomics reveals global architecture of single nuclei. *Nature* 590 344–350. 10.1038/s41586-020-03126-2 33505024 PMC7878433

[B290] Tang G. B. Zeng Y. Q. Liu P. P. Mi T. W. Zhang S. F. Dai S. K. (2017). The histone H3K27 demethylase UTX regulates synaptic plasticity and cognitive behaviors in mice. *Front. Mol. Neurosci.* 10:267. 10.3389/fnmol.2017.00267 28970783 PMC5609596

[B291] Tardat M. Brustel J. Kirsh O. Lefevbre C. Callanan M. Sardet C. (2010). The histone H4 Lys 20 methyltransferase PR-Set7 regulates replication origins in mammalian cells. *Nat. Cell Biol.* 12 1086–1093. 10.1038/ncb2113 20953199

[B292] Terzi M. Y. Izmirli M. Gogebakan B. (2016). The cell fate: senescence or quiescence. *Mol. Biol. Rep.* 43 1213–1220. 27558094 10.1007/s11033-016-4065-0

[B293] Tesio M. Tang Y. Mudder K. Saini M. von Paleske L. Macintyre E. (2015). Hematopoietic stem cell quiescence and function are controlled by the CYLD-TRAF2-p38MAPK pathway. *J. Exp. Med.* 212 525–538. 10.1084/jem.20141438 25824820 PMC4387289

[B294] Tessarz P. Kouzarides T. (2014). Histone core modifications regulating nucleosome structure and dynamics. *Nat. Rev. Mol. Cell Biol.* 15 703–708. 25315270 10.1038/nrm3890

[B295] Tie G. Yan J. Khair L. Tutto A. Messina L. M. (2020). Hypercholesterolemia accelerates the aging phenotypes of hematopoietic stem cells by a Tet1-dependent pathway. *Sci. Rep.* 10:3567. 10.1038/s41598-020-60403-w 32107419 PMC7046636

[B296] Tokuyasu K. Madden S. C. Zeldis L. J. (1968). Fine structural alterations of interphase nuclei of lymphocytes stimulated to grwoth activity in vitro. *J. Cell Biol.* 39 630–660. 10.1083/jcb.39.3.630 5699935 PMC2107542

[B297] Torres I. O. Kuchenbecker K. M. Nnadi C. I. Fletterick R. J. Kelly M. J. Fujimori D. G. (2015). Histone demethylase KDM5A is regulated by its reader domain through a positive-feedback mechanism. *Nat. Commun.* 6:6204. 10.1038/ncomms7204 25686748 PMC5080983

[B298] Tripputi P. Emanuel B. S. Croce C. M. Green L. G. Stein G. S. Stein J. L. (1986). Human histone genes map to multiple chromosomes. *Proc. Natl. Acad. Sci. U.S.A.* 83 3185–3188. 3458175 10.1073/pnas.83.10.3185 PMC323477

[B299] Trojer P. Reinberg D. (2007). Facultative heterochromatin: is there a distinctive molecular signature? *Mol. Cell* 28 1–13. 10.1016/j.molcel.2007.09.011 17936700

[B300] Trojer P. Li G. Sims R. J. III Vaquero A. Kalakonda N. Boccuni P. (2007). L3MBTL1, a histone-methylation-dependent chromatin lock. *Cell* 129 915–928. 10.1016/j.cell.2007.03.048 17540172

[B301] Tsai W. W. Wang Z. Yiu T. T. Akdemir K. C. Xia W. Winter S. (2010). TRIM24 links a non-canonical histone signature to breast cancer. *Nature* 468 927–932. 10.1038/nature09542 21164480 PMC3058826

[B302] Tumpel S. Rudolph K. L. (2019). Quiescence: good and bad of stem cell aging. *Trends Cell Biol.* 29 672–685. 10.1016/j.tcb.2019.05.002 31248787

[B303] Turner B. M. (2002). Cellular memory and the histone code. *Cell* 111 285–291. 12419240 10.1016/s0092-8674(02)01080-2

[B304] Urbán N. Cheung T. H. (2021). Stem cell quiescence: the challenging path to activation. *Development* 148 10.1242/dev.165084 33558315 PMC7888710

[B305] Vakoc C. R. Mandat S. A. Olenchock B. A. Blobel G. A. (2005). Histone H3 lysine 9 methylation and HP1gamma are associated with transcription elongation through mammalian chromatin. *Mol. Cell* 19 381–391. 16061184 10.1016/j.molcel.2005.06.011

[B306] Valcourt J. R. Lemons J. M. Haley E. M. Kojima M. Demuren O. O. Coller H. A. (2012). Staying alive: metabolic adaptations to quiescence. *Cell Cycle* 11 1680–1696. 10.4161/cc.19879 22510571 PMC3372394

[B307] van Leeuwen F. Gafken P. R. Gottschling D. E. (2002). Dot1p modulates silencing in yeast by methylation of the nucleosome core. *Cell* 109 745–756. 10.1016/s0092-8674(02)00759-6 12086673

[B308] van Velthoven C. T. J. de Morree A. Egner I. M. Brett J. O. Rando T. A. (2017). Transcriptional profiling of quiescent muscle stem cells in vivo. *Cell Rep.* 21 1994–2004. 29141228 10.1016/j.celrep.2017.10.037 PMC5711481

[B309] Vander Heiden M. G. Cantley L. C. Thompson C. B. (2009). Understanding the Warburg effect: the metabolic requirements of cell proliferation. *Science* 324 1029–1033. 19460998 10.1126/science.1160809 PMC2849637

[B310] Vasquez J. J. Wedel C. Cosentino R. O. Siegel T. N. (2018). Exploiting CRISPR-Cas9 technology to investigate individual histone modifications. *Nucleic Acids Res.* 46:e106. 10.1093/nar/gky517 29912461 PMC6182134

[B311] Venezia T. A. Merchant A. A. Ramos C. A. Whitehouse N. L. Young A. S. Shaw C. A. (2004). Molecular signatures of proliferation and quiescence in hematopoietic stem cells. *PLoS Biol.* 2:e301. 10.1371/journal.pbio.0020301 15459755 PMC520599

[B312] Venkatraman A. He X. C. Thorvaldsen J. L. Sugimura R. Perry J. M. Tao F. (2013). Maternal imprinting at the H19-Igf2 locus maintains adult haematopoietic stem cell quiescence. *Nature* 500 345–349. 10.1038/nature12303 23863936 PMC3896866

[B313] Vizán P. Gutiérrez A. Espejo I. García-Montolio M. Lange M. Carretero A. (2020). The Polycomb-associated factor PHF19 controls hematopoietic stem cell state and differentiation. *Sci. Adv.* 6:eabb2745. 10.1126/sciadv.abb2745 32821835 PMC7406347

[B314] Voigt P. Tee W. W. Reinberg D. (2013). A double take on bivalent promoters. *Genes Dev.* 27 1318–1338. 10.1101/gad.219626.113 23788621 PMC3701188

[B315] Volker-Albert M. C. Schmidt A. Forne I. Imhof A. (2018). Analysis of histone modifications by mass spectrometry. *Curr. Protoc. Protein Sci.* 92:e54. 10.1002/cpps.54 30040183

[B316] Wagner E. J. Carpenter P. B. (2012). Understanding the language of Lys36 methylation at histone H3. *Nat Rev. Mol. Cell Biol.* 13 115–126. 10.1038/nrm3274 22266761 PMC3969746

[B317] Walter D. Matter A. Fahrenkrog B. (2014). Loss of histone H3 methylation at lysine 4 triggers apoptosis in *Saccharomyces cerevisiae*. *PLoS Genet.* 10:e1004095. 10.1371/journal.pgen.1004095 24497836 PMC3907299

[B318] Wang D. Mansisidor A. Prabhakar G. Hochwagen A. (2016). Condensin and Hmo1 mediate a starvation-induced transcriptional position effect within the ribosomal DNA array. *Cell Rep.* 17:624. 10.1016/j.celrep.2016.09.057 27705806

[B319] Wang G. G. Allis C. D. (2009). “Misinterpretation” of a histone mark is linked to aberrant stem cells and cancer development. *Cell Cycle* 8 1982–1983. 19550157

[B320] Wang Z. Gerstein M. Snyder M. (2009). RNA-Seq: a revolutionary tool for transcriptomics. *Nat. Rev. Genet.* 10 57–63. 19015660 10.1038/nrg2484 PMC2949280

[B321] Wang Z. Zang C. Rosenfeld J. A. Schones D. E. Barski A. Cuddapah S. (2008). Combinatorial patterns of histone acetylations and methylations in the human genome. *Nat. Genet.* 40 897–903. 18552846 10.1038/ng.154 PMC2769248

[B322] Watanabe S. Mishima Y. Shimizu M. Suetake I. Takada S. (2018). Interactions of HP1 bound to H3K9me3 dinucleosome by molecular simulations and biochemical assays. *Biophys. J.* 114 2336–2351. 10.1016/j.bpj.2018.03.025 29685391 PMC6129468

[B323] Wei Y. Yu L. Bowen J. Gorovsky M. A. Allis C. D. (1999). Phosphorylation of histone H3 is required for proper chromosome condensation and segregation. *Cell* 97 99–109. 10199406 10.1016/s0092-8674(00)80718-7

[B324] Werner-Washburne M. Braun E. L. Crawford M. E. Peck V. M. (1996). Stationary phase in *Saccharomyces cerevisiae*. *Mol. Microbiol.* 19 1159–1166. 8730858 10.1111/j.1365-2958.1996.tb02461.x

[B325] Wiles E. T. Selker E. U. (2017). H3K27 methylation: a promiscuous repressive chromatin mark. *Curr. Opin. Genet. Dev.* 43 31–37. 10.1016/j.gde.2016.11.001 27940208 PMC5447479

[B326] Wilson A. Laurenti E. Oser G. van der Wath R. C. Blanco-Bose W. Jaworski M. (2008). Hematopoietic stem cells reversibly switch from dormancy to self-renewal during homeostasis and repair. *Cell* 135 1118–1129. 10.1016/j.cell.2008.10.048 19062086

[B327] Winter S. Simboeck E. Fischle W. Zupkovitz G. Dohnal I. Mechtler K. (2008). 14-3-3 proteins recognize a histone code at histone H3 and are required for transcriptional activation. *EMBO J.* 27 88–99. 10.1038/sj.emboj.7601954 18059471 PMC2206135

[B328] Wu S. Wang W. Kong X. Congdon L. M. Yokomori K. Kirschner M. W. (2010). Dynamic regulation of the PR-Set7 histone methyltransferase is required for normal cell cycle progression. *Genes Dev.* 24 2531–2542. 10.1101/gad.1984210 20966048 PMC2975929

[B329] Wu T. Yoon H. Xiong Y. Dixon-Clarke S. E. Nowak R. P. Fischer E. S. (2020). Targeted protein degradation as a powerful research tool in basic biology and drug target discovery. *Nat. Struct. Mol. Biol.* 27 605–614. 32541897 10.1038/s41594-020-0438-0 PMC7923177

[B330] Xu J. Kidder B. L. (2018). H4K20me3 co-localizes with activating histone modifications at transcriptionally dynamic regions in embryonic stem cells. *BMC Genomics* 19:514. 10.1186/s12864-018-4886-4 29969988 PMC6029396

[B331] Xu J. Ma H. Jin J. Uttam S. Fu R. Huang Y. (2018). Super-Resolution imaging of higher-order chromatin structures at different epigenomic states in single mammalian cells. *Cell Rep.* 24 873–882. 30044984 10.1016/j.celrep.2018.06.085 PMC6154382

[B332] Xu M. Soloveychik M. Ranger M. Schertzberg M. Shah Z. Raisner R. (2012). Timing of transcriptional quiescence during gametogenesis is controlled by global histone H3K4 demethylation. *Dev. Cell* 23 1059–1071. 10.1016/j.devcel.2012.10.005 23123093 PMC3806484

[B333] Yan H. Evans J. Kalmbach M. Moore R. Middha S. Luban S. (2014). HiChIP: a high-throughput pipeline for integrative analysis of ChIP-Seq data. *BMC Bioinformatics* 15:280. 10.1186/1471-2105-15-280 25128017 PMC4152589

[B334] Yang K. Chi H. (2018). Investigating cellular quiescence of T lymphocytes and antigen-induced exit from quiescence. *Methods Mol. Biol.* 1686 161–172. 10.1007/978-1-4939-7371-2_12 29030820

[B335] Yildirim E. Kirby J. E. Brown D. E. Mercier F. E. Sadreyev R. I. Scadden D. T. (2013). Xist RNA is a potent suppressor of hematologic cancer in mice. *Cell* 152 727–742. 10.1016/j.cell.2013.01.034 23415223 PMC3875356

[B336] Yin H. Price F. Rudnicki M. A. (2013). Satellite cells and the muscle stem cell niche. *Physiol. Rev.* 93 23–67. 23303905 10.1152/physrev.00043.2011 PMC4073943

[B337] Young C. P. Hillyer C. Hokamp K. Fitzpatrick D. J. Konstantinov N. K. Welty J. S. (2017). Distinct histone methylation and transcription profiles are established during the development of cellular quiescence in yeast. *BMC Genomics* 18:107. 10.1186/s12864-017-3509-9 28122508 PMC5267397

[B338] Yuan W. Xu M. Huang C. Liu N. Chen S. Zhu B. (2011). H3K36 methylation antagonizes PRC2-mediated H3K27 methylation. *J. Biol. Chem.* 286 7983–7989. 21239496 10.1074/jbc.M110.194027 PMC3048685

[B339] Zahedi Y. Durand-Dubief M. Ekwall K. (2020). High-Throughput flow cytometry combined with genetic analysis brings new insights into the understanding of chromatin regulation of cellular quiescence. *Int J Mol Sci.* 21:9022. 10.3390/ijms21239022 33260998 PMC7729564

[B340] Zhang T. Cooper S. Brockdorff N. (2015). The interplay of histone modifications - writers that read. *EMBO Rep.* 16 1467–1481. 26474904 10.15252/embr.201540945 PMC4641500

[B341] Zhou K. Gaullier G. Luger K. (2019). Nucleosome structure and dynamics are coming of age. *Nat. Struct. Mol. Biol.* 26 3–13. 30532059 10.1038/s41594-018-0166-x PMC7386248

[B342] Zhou Y. Yan X. Feng X. Bu J. Dong Y. Lin P. (2018). Setd2 regulates quiescence and differentiation of adult hematopoietic stem cells by restricting RNA polymerase II elongation. *Haematologica* 103 1110–1123. 10.3324/haematol.2018.187708 29650642 PMC6029524

[B343] Zhu C. Zhang Y. Li Y. E. Lucero J. Behrens M. M. Ren B. (2021). Joint profiling of histone modifications and transcriptome in single cells from mouse brain. *Nat. Methods* 18 283–292. 10.1038/s41592-021-01060-3 33589836 PMC7954905

[B344] Zippo A. Serafini R. Rocchigiani M. Pennacchini S. Krepelova A. Oliviero S. (2009). Histone crosstalk between H3S10ph and H4K16ac generates a histone code that mediates transcription elongation. *Cell* 138 1122–1136. 10.1016/j.cell.2009.07.031 19766566

